# Semi-supervised machine learning approaches for predicting the chronology of archaeological sites: A case study of temples from medieval Angkor, Cambodia

**DOI:** 10.1371/journal.pone.0205649

**Published:** 2018-11-05

**Authors:** Sarah Klassen, Jonathan Weed, Damian Evans

**Affiliations:** 1 Arizona State University, School of Social Change and Human Evolution, Tempe, AZ, United States of America; 2 Massachusetts Institute of Technology, Department of Mathematics, Cambridge, MA, United States of America; 3 École française d'Extrême-Orient, Paris, France; Max Planck Institute for the Science of Human History, GERMANY

## Abstract

Archaeologists often need to date and group artifact types to discern typologies, chronologies, and classifications. For over a century, statisticians have been using classification and clustering techniques to infer patterns in data that can be defined by algorithms. In the case of archaeology, linear regression algorithms are often used to chronologically date features and sites, and pattern recognition is used to develop typologies and classifications. However, archaeological data is often expensive to collect, and analyses are often limited by poor sample sizes and datasets. Here we show that recent advances in computation allow archaeologists to use machine learning based on much of the same statistical theory to address more complex problems using increased computing power and larger and incomplete datasets. This paper approaches the problem of predicting the chronology of archaeological sites through a case study of medieval temples in Angkor, Cambodia. For this study, we have a large dataset of temples with known architectural elements and artifacts; however, less than ten percent of the sample of temples have known dates, and much of the attribute data is incomplete. Our results suggest that the algorithms can predict dates for temples from 821–1150 CE with a 49-66-year average absolute error. We find that this method surpasses traditional supervised and unsupervised statistical approaches for under-specified portions of the dataset and is a promising new method for anthropological inquiry.

## Introduction

Archaeologists often rely on statistical methods to infer the chronology of and group archaeological sites, artifact types, and architecture. However, this can be limited by incomplete datasets. It can be relatively easy to create large archaeological datasets, with excavations producing thousands of ceramic sherds and lithic assemblages. Similarly, hundreds of archaeological sites can be identified on the landscape using remote sensing at relatively little cost. However, determining the chronology of the sites using excavation and C14 dating methods or assigning ceramics to group using INAA analyses is comparatively expensive and time consuming. As such, archaeologists often have large inventories of artifacts and sites, but the majority of the data points are underspecified because the chronology and group classifications are unknown and expensive to obtain using traditional methods. In this paper, we introduce the use of semi-supervised machine learning algorithms for archaeological inquiry. Machine learning mimics human pattern recognition and learning processes through a series of complex mathematical computations to find structure and define algorithms for large datasets [1]. In this scenario, algorithms refer to the equation, rules, or set of steps and pattern recognition necessary to transform the data (input) into the categories (output) [2]. Pattern recognition is the process of finding structure in data that can be used to divide the data into discrete categories [1].

**Fig 1:**
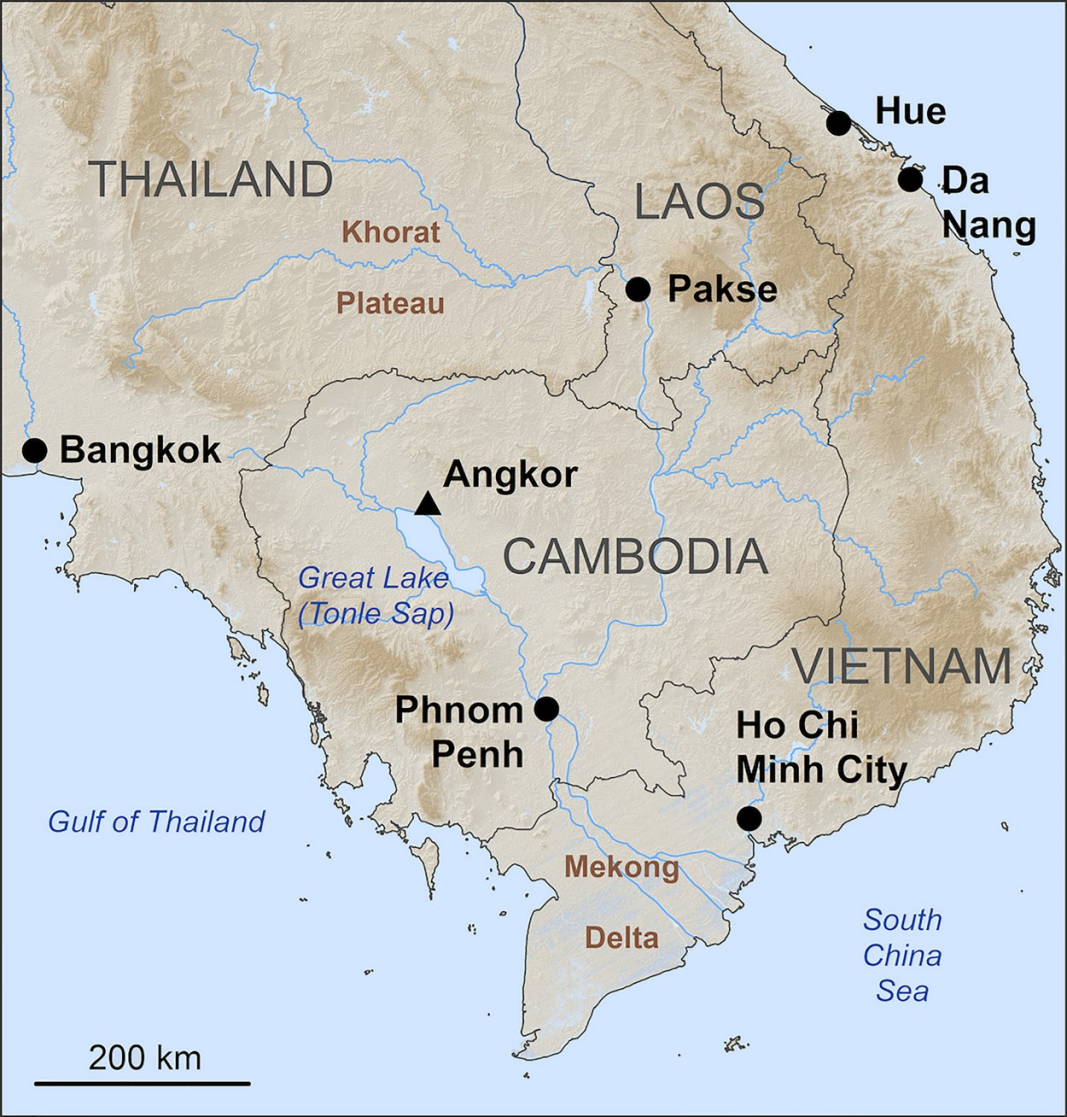
Location of Angkor in Cambodia. This figure includes SRTM data, which has been released and distributed without restrictions.

Our case study, Angkor, was the political center of the Khmer Empire (9^th^– 15^th^ centuries CE) in present-day Cambodia for over five hundred years (Fig 1). During this time, over 1400 temples were constructed in the greater Angkor region that were economic and religious centers of residential hamlets. Several mapping projects have shown the relationship between temples and other urban features, like occupation mounds and reservoirs [1–3]. We argue that by dating the temples, we can also date associated urban features to create historical models of urban morphology, which will allow us to conduct more sophisticated analyses of the development of the urban center over time. Ideally, we would like to create historical models for each century for future studies evaluating changes in the landscape, water management system, and agricultural system over time. Given that Angkor lasted as a political center for over five hundred years, being able to create historical models of the urban morphology for each century provides us with five time slices for diachronic comparative analyses.

In this paper, we first introduce statistical learning paradigms and our archaeological case study and dataset. We then explore four classical mathematical approaches to find statistically significant predictors for temple dates. We find that k-means clustering, discriminant function analysis, and principal component analysis cannot accurately predict temple dates to within 100-year time periods. Multiple linear regression can predict temples with a low absolute average error. However, it only works on well-specified data-points and cannot predict dates for approximately half of the temples. We then introduce semi-supervised machine learning as a potential method to address some of the inadequacies of supervised and unsupervised statistical paradigms. Our results indicate that graph-based semi-supervised machine learning, unlike multiple linear regression, can predict dates for all the temples in the dataset. When combined with the results of the multiple linear regression for more-specified data, we can create a historical model of urban development in terms of temple dates at Angkor for temples constructed between 821–1149 CE with an absolute average error (AAE) of 49–66 years.

## Statistical paradigms

### Statistical paradigms: Supervised, unsupervised, and semi-supervised

The degree of completeness of a given dataset defines the type of statistical learning paradigms possible [4]. As in traditional statistical analyses, the goal of machine learning algorithms is to infer information on the basis of incomplete data. One prototypical problem is to classify data points by assigning each data point a “label” reflecting a quantity of interest. For example, we are interested in dating temples; temples with known dates are considered labeled data and temples without known dates are considered unlabeled data. In general, there are three types of learning paradigms: supervised (all data are labeled), unsupervised (no data are labeled), and semi-supervised (a portion of the data are labeled).

In the following sections, we discuss the differences between supervised, unsupervised, and semi-supervised machine learning. Note that supervised and unsupervised paradigms also apply to non-machine learning statistical analyses. The analyses we performed in this paper encompassed both supervised and unsupervised paradigms.

#### Supervised machine learning

Analyses that use labeled data are “supervised” because we know the correct output, which allows us to correct errors in the algorithm [5]. Some examples of machine learning applications that use supervised paradigms are associations, classification, and regression. Machine learning *associations* identify conditional probability in sets of data among input variables and between input variables and outputs [6]. For example, machine learning can associate products customers typically buy together, like cereal and milk. The association of cereal and milk can be used by companies to cross-sell and advertise milk to customers purchasing cereal.

Supervised machine learning can also *classify* data into discrete classes. Insurance companies use a wide assortment of data about insurance applicants (e.g., age, income, sex, history) to classify them into high and low-risk groups. This machine learning method relies on previously collected data about individuals including their attributes (e.g., age, income, sex, history) and their insurance claims. By classifying new customers into low or high-risk groups, the insurance provider can determine which types of insurance to offer and determine premium rates. Classification algorithms are created with pre-existing data, but they can be adjusted as future data become available to improve accuracy. Other examples of machine learning classification include image and text recognition [6].

*Regression* is distinguished from classification because the output is continuous as opposed to discrete. For example, a machine learning regression can predict the price of houses based on a training set of houses’ attributes (e.g., type of countertop, square feet, neighborhood) and known sale prices. Machine learning optimizes the algorithm, so the approximate error of the value is as minimal as possible based on the known prices of houses in the training set [6].

#### Unsupervised machine learning

For unsupervised learning, all the data are unlabeled [5]. Unsupervised learning works best to identify underlying patterns or structures in data [6, 7]. While unsupervised learning is fundamentally used for estimating density, it can also be used for quantile estimation, clustering, outlier detection, and dimensionality reduction [7]. For example, companies can use unsupervised learning paradigms to group customers based on demographic information and purchasing habits. The companies can then target different groups for marketing and outliers can be identified as niche markets.

#### Semi-supervised machine learning

Semi-supervised learning (SSL) lies between supervised and unsupervised learning paradigms by incorporating both labeled and unlabeled data. This approach is often used when labeled data points are few because they are time consuming or expensive to obtain. In many cases, a fully labeled dataset may be infeasible, whereas non-labeled data points may be easily obtained [7, 8]. The internet, for example, has provided an avenue to easily and inexpensively obtaining unlabeled data through web crawlers. Web crawlers can scrape millions of photographs from the internet. However, to label these images would require much human effort to identify and record the content of each image by hand. SSL works to minimize the number of labels needed by learning from unlabeled data, thereby reducing the necessary human effort. One of the first SSL algorithms was developed to classify web pages [9].

**Fig 2:**
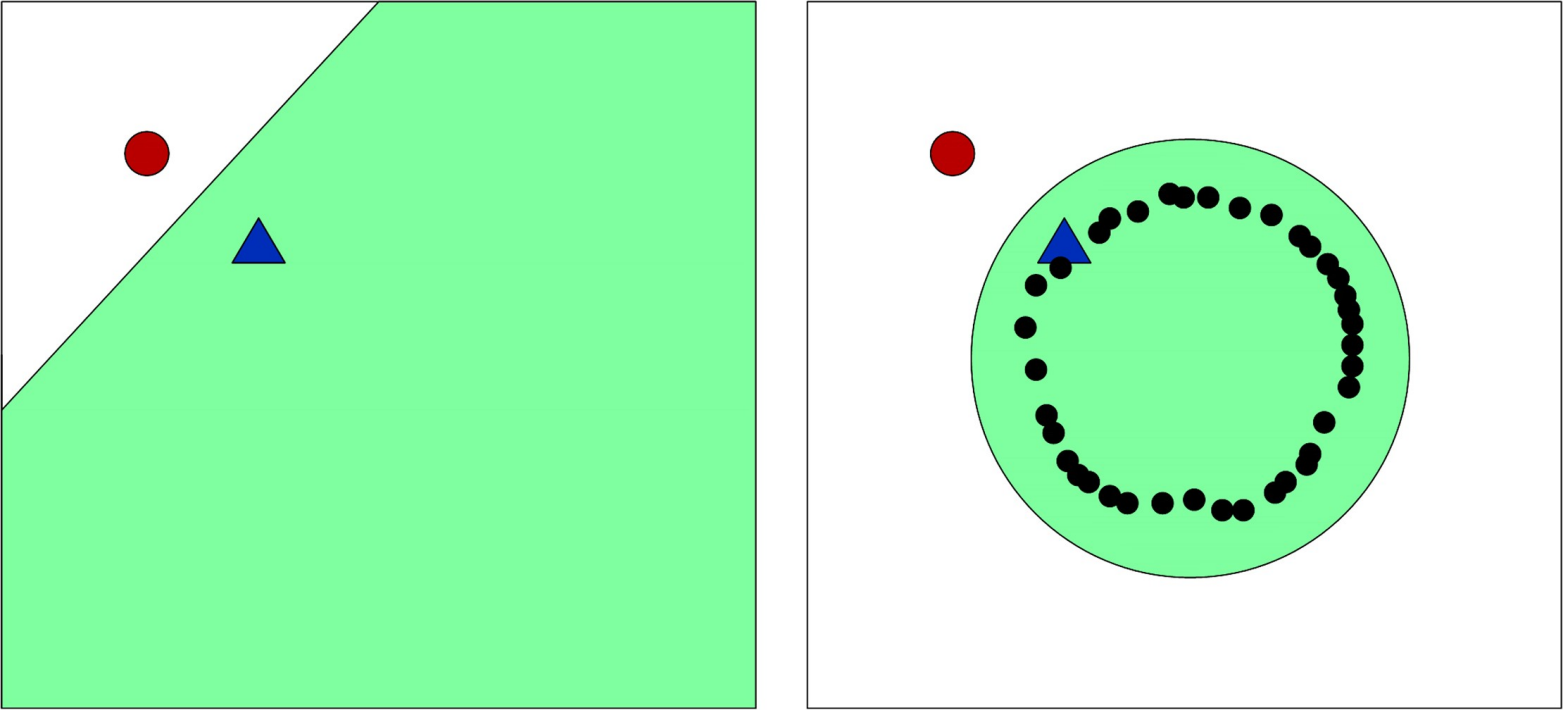
Labeled data with prior classification (left) compared with altered classification as a result of the addition of unlabeled data. Example drawn and discussed further in Belkin 2006.

SSL creates algorithms that use unlabeled data to improve the supervised learning algorithms [9]. It may seem counterintuitive to suggest that one can use unlabeled data to learn the labels of other data, but the distribution of unlabeled data in relation to labeled data can reveal a great deal of information. Fig 2 illustrates how unlabeled data can be used with labeled data to infer underlying patterns. In this example, there are two labeled data points, a circle and a diamond. Many statistical methods (e.g., Bayesian paradigms, regularization, minimum description length) would linearly divide the space as shown on the left. However, if we introduce unlabeled data, a geometric structure emerges that contradicts the linear divide. Instead, a circular classifier is preferred [10].

Indeed, much of natural human learning occurs in SSL paradigms [10]. Take, for example, how children learn to classify objects. They are exposed to some labeled data, their parent pointing to a gray fluffy animal, “cat.” However, they also observe many animals that are not explicitly labeled. Over time, children combine both the labeled and unlabeled data as they learn to classify animals [11].

If the data are unlabeled, how do we know if SSL works? In some cases, it is possible to identify isolated errors. For example, the number of labeled data points for image recognition SSL is limited by the relatively expensive human component of hand labeling. In these cases, the labels are not truly unknown, only in the context of the training dataset used by the learning algorithm. As such, humans can easily verify the results by scanning through the classification of images and recognizing mistakes. Mistakes can then be rectified to improve the overall accuracy of the model. A classic example is the individuals incorrectly classified as gorillas by Google’s image classifier in 2015. The individuals brought the error to the attention of Google engineers, who quickly rectified the mistake [12]. When the labels are truly unknown, the standard way to evaluate machine learning algorithms and estimate prediction error is through cross-validation [13].

## Background: Case study and data

**Fig 3:**
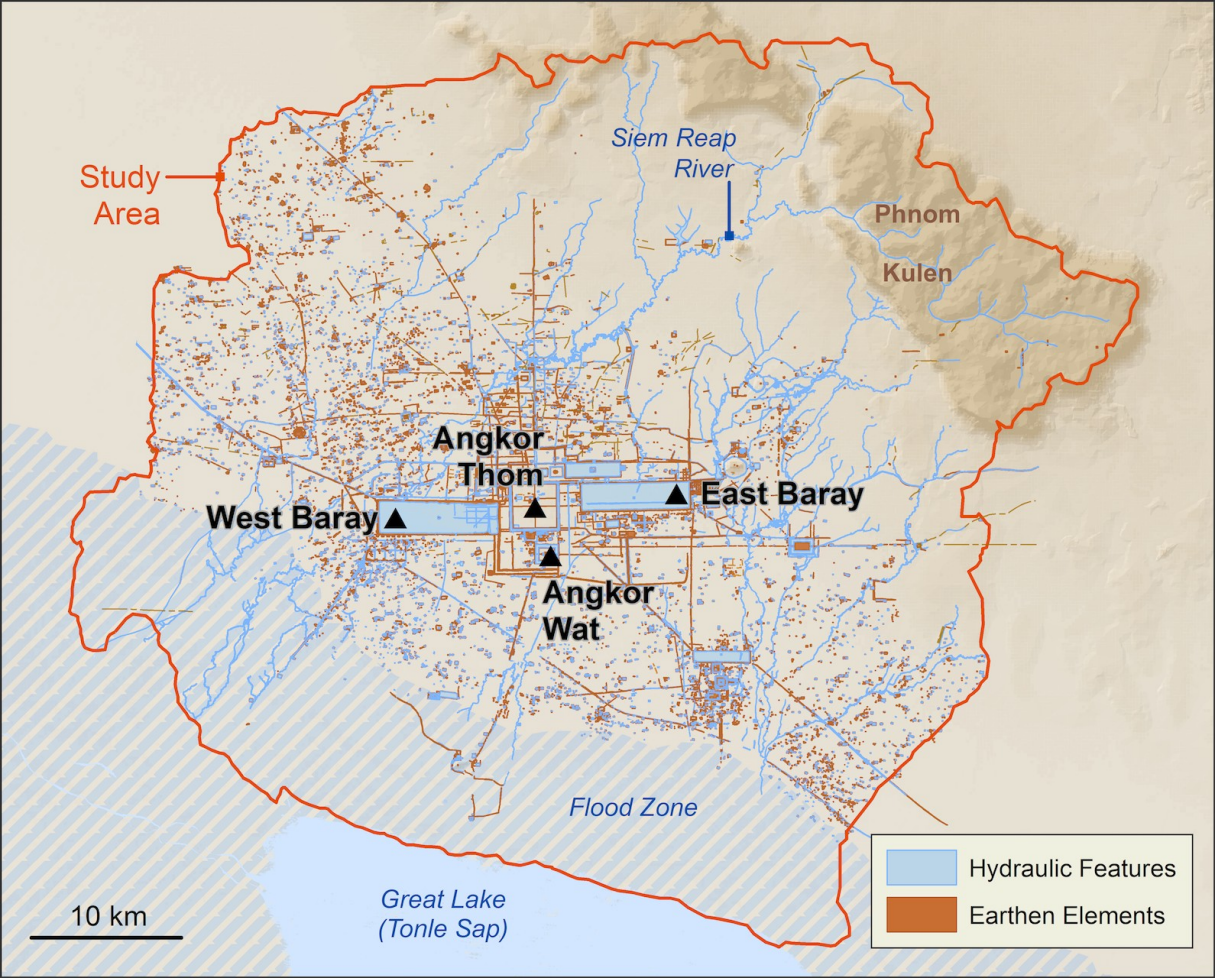
Map of Angkor created by Evans and Pottier. This figure includes SRTM data, which has been released and distributed without restrictions.

Angkor is a sprawling low-density urban complex with hundreds of temples and occupation mounds connected through a network of hydraulic infrastructure [14]. Until recently, the full extent of the settlement was only partially understood. Much of the habitational space was constructed in non-durable organic materials that have since disintegrated. Decades of aerial mapping and other remote sensing, however, have revealed traces of archaeological features including ponds, occupation mounds, embankments, and channels on the landscape [1, 15]. Evans and Pottier mapped much of the hinterlands and identified over 1400 temples (**Fig 3**). In this paper, we are interested in identifying the construction sequence of these temples so that we can date other urban features by proxy and create historical models of the urban development of the city.

The archaeological record at Angkor is a palimpsest of thousands of years of human habitation, with early urban forms emerging in the Bronze and Iron Ages and developing, in the first millennium CE, into dispersed, low-density settlement complexes punctuated by high-density epicenters and nodes [16]. While much work has been done using inscriptions and art historical inference to dates temples, the sheer scale and intensity of human transformation of the landscape, combined with persistent occupation and renovation of settlements over millennia, makes understanding the chronology of Angkor difficult.

In the following sections, we describe prior work that has been done to date temples at Angkor, primarily through Lustig’s interpretation of temple inscriptions with listed dates and dates derived from Polkinghorne’s dating of lintels [17, 18]. In total, there are 1437 temples in Cambodia. Of these, 105 of the temples have known dates from Lustig and Polkinghorne (Appendix 1). Our goal is to identify dates for the remaining 1332 undated temples using statistical methods to analyze a variety of attribute data of temples that, prior to this study, have not been found statistically useful for determining chronological patterns.

A further complication is that a single temple may have had multiple periods of occupation. Some were used for 100 years, then abandoned, and then re-purposed 300 years later. For example, one temple, *Kapilapura*, has inscriptions dating to 968 CE and 1200–1399 CE, suggesting at least two periods of occupation. Others were built over and obscured from our current record. Given the nature of the archaeological record, in most cases, it is easiest to determine when the temples were initially built, or *terminus post quem*. We do not expect to be able to identify multiple phases of occupation unless there are multiple art historical periods, inscriptions, or extensive excavation. When a temple went out of use, or *terminus ante quem*, is also difficult to determine. However, Greater Angkor Project (GAP) III ceramic data and excavations of temple sites by Pierre Bâty suggest degrees of longevity [19]. As a result, we treat the temple dates as cumulative, meaning that once built, a temple is in continued use unless we have specific spatiotemporal data to suggest it went into disuse.

### Data

**Fig 4:**
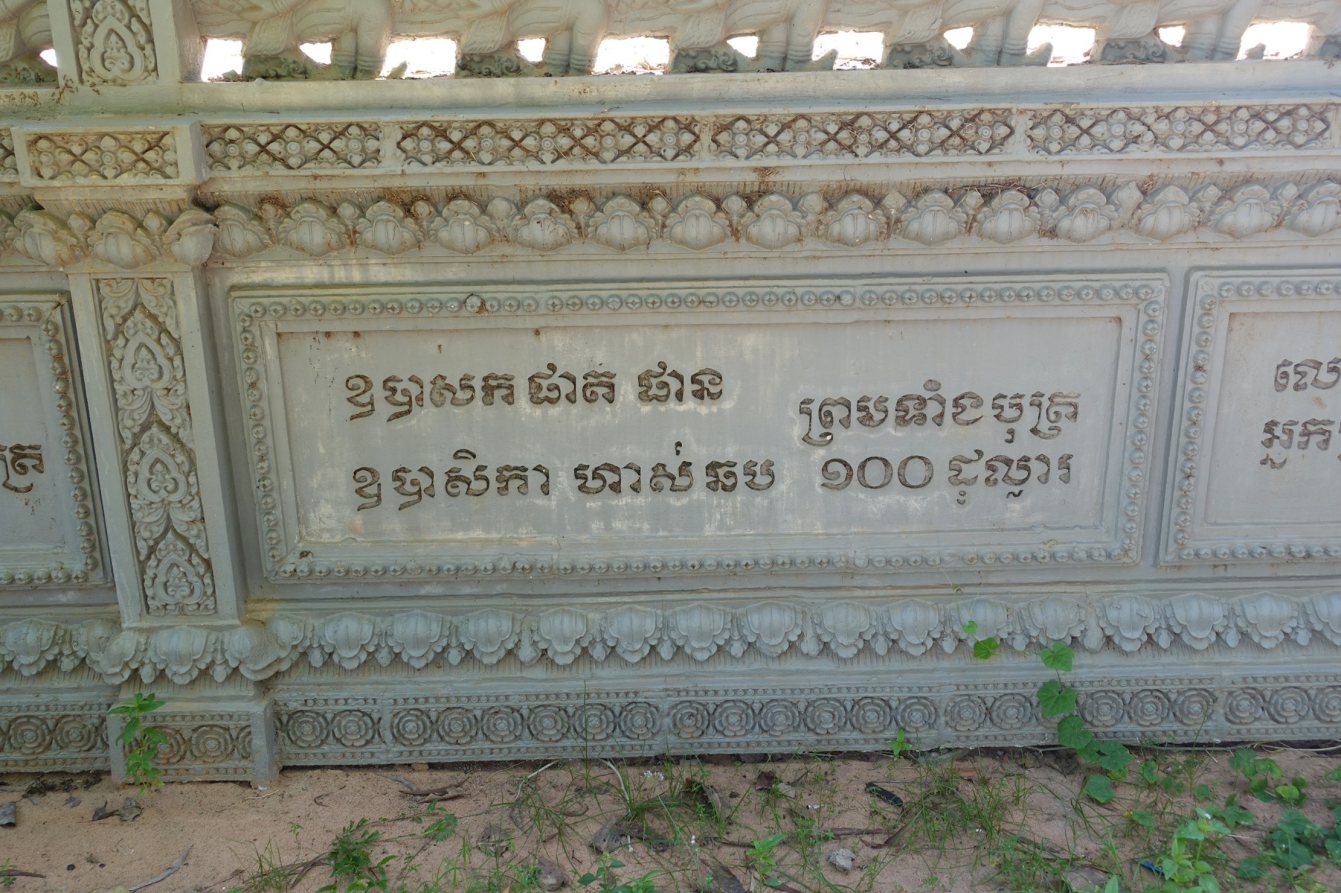
Contemporary Khmer pagodas list individuals who donated to the construction of the temple. This photograph was taken by Sarah Klassen.

Angkorian inscriptions were inscribed on stone in Khmer, Sanskrit, or both and often refer to temple foundations, including their establishment, administration, and support [20, 21]. Meaning that when temples were established the date of their construction and the name of the sponsor were inscribed on the temple walls. Similar inscriptions on contemporary pagodas indicate individual contributions to Pagoda foundations (**Fig 4**). As such, temples with inscriptions can be dated to a high degree of accuracy. However, inscriptions were expensive and only a small proportion (roughly 10%) of temples in the greater Angkor region have inscriptions. Similarly, the temples that do have inscriptions tend to be larger in size. Where specific foundation dates were listed, Lustig converted the śaka dates to CE by adding 78 years. Saka dates are years in the Indian calendar, which begin at the start of the Saka Era (March 22^nd^ of year 79 in the Gregorian calendar). Temples with śaka dates are considered “certain.” Where inscriptions were undated or a century or even two centuries are suggested, Lustig converted these to the approximate CE centuries [22]. For example, she converted 9^th^ century śaka (800–899 śaka or 878–977 CE) to 10^th^ century CE (900–999 CE). She further narrowed date ranges to specific reigns mentioned in the inscription. For example, if a king was mentioned by name, the date range was adjusted to the known dates of that king’s reign. If a king’s posthumous name (the name the king was referred to after death) was given, Lustig determined that inscription must have been written after his death and she adjusted the date range accordingly. Lustig considered dates with ranges “uncertain.”

For the statistical analysis, we are interested in identifying the date most consistent with the current attributes of the temple. For example, it is possible that a sandstone temple from the 11^th^ century CE was built on top of a small shrine dating to a much earlier period. If our attribute data for that temple represents the construction in the 11^th^ century CE, we are interested in associating the temple with the 11^th^ century CE date for the statistical analysis, regardless of whether there was an earlier foundation. We acknowledge that not being able to identify earlier foundations is a limitation in our study; however, at the present time the only way to identify earlier foundations is through extensive excavations at each temple site. Which would be prohibitively expensive and time consuming. Multiple periods of occupation are added to the model of urban development after we have conducted the statistical analysis. Where the date listed for each inscription or lintel was a range, we opted to use the median of the range.

The second source of temple dates are lintels. Many smaller temples, that did not have inscriptions, had carved lintels. Lintels depict scenes from mythology and decorative motifs. The styles of the lintels changed over time and Polkinghorne was able to compare these styles with styles from temples with known dates to determine a chronology of lintels. Polkinghorne also used the designation of “certain” vs. “uncertain” for lintel dates based on multiple lines of evidence, including the inscriptional data [17] (S1 Table)

Temples with multiple inscriptions and lintel dates were dated as follows with “certain” dates always prioritized over “uncertain” dates:

- If there was only one lintel or inscription date, we used the date.
- If there are multiple inscription and/or lintel dates that were within 65 years of each other, we used the median of the dates. 65 years is somewhat arbitrary, but we argue that it accounts for variability in the original data, especially when dates were assigned based on the reigns of kings.
- If there are no inscription or lintel dates, we use the dates found through literature searches.
- When there are conflicting dates from the inscriptions and lintels where literature searches did not help, we prioritized the dates in the following order: lintels (certain), inscriptions (certain), lintels (uncertain), and inscriptions (uncertain). These have multiple periods of occupation.

**Fig 5:**
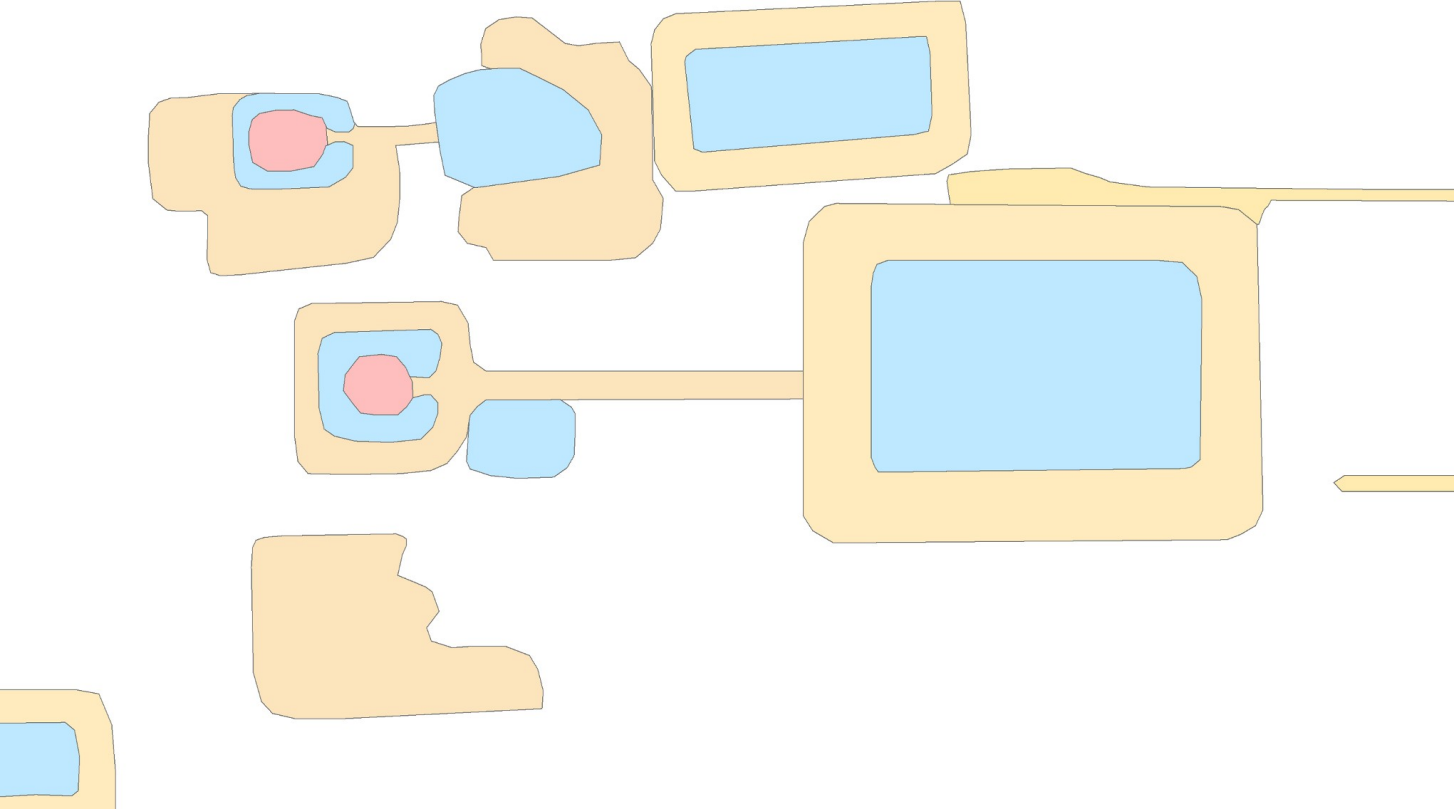
A temple (red) with a primary reservoir.

**Fig 6:**
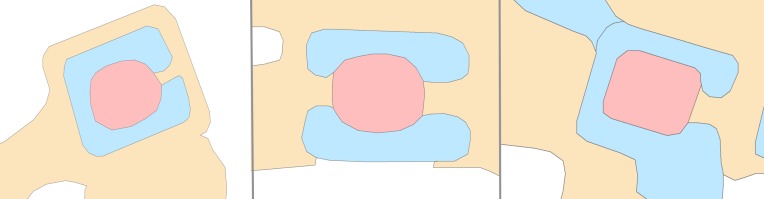
65, 90, and 115 degree examples of temple (red) azimuths.

**Fig 7:**
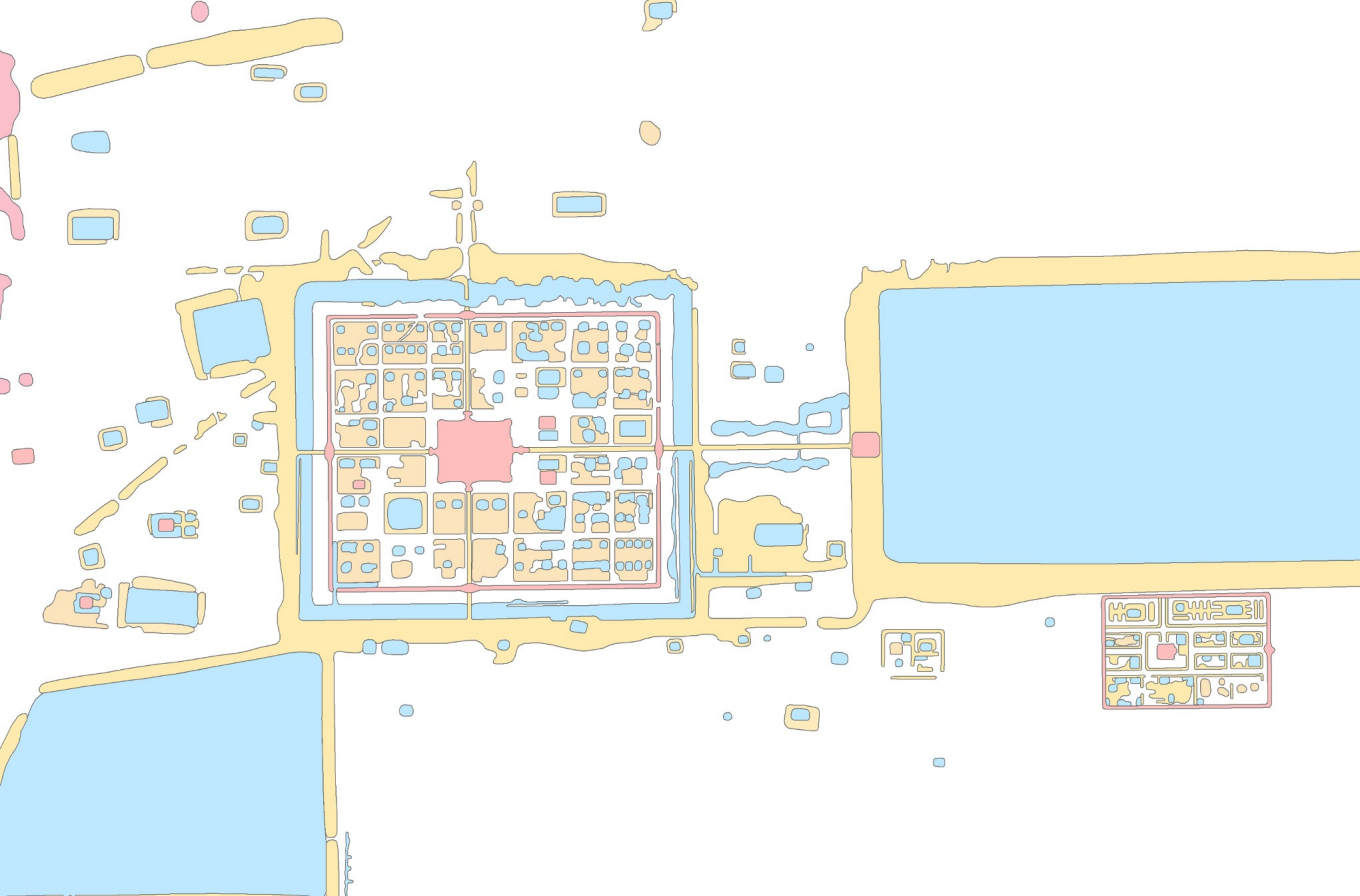
Example of temple (red) area. Note the large temple in the middle with small temples to the south and west indicated in red.

**Fig 8:**
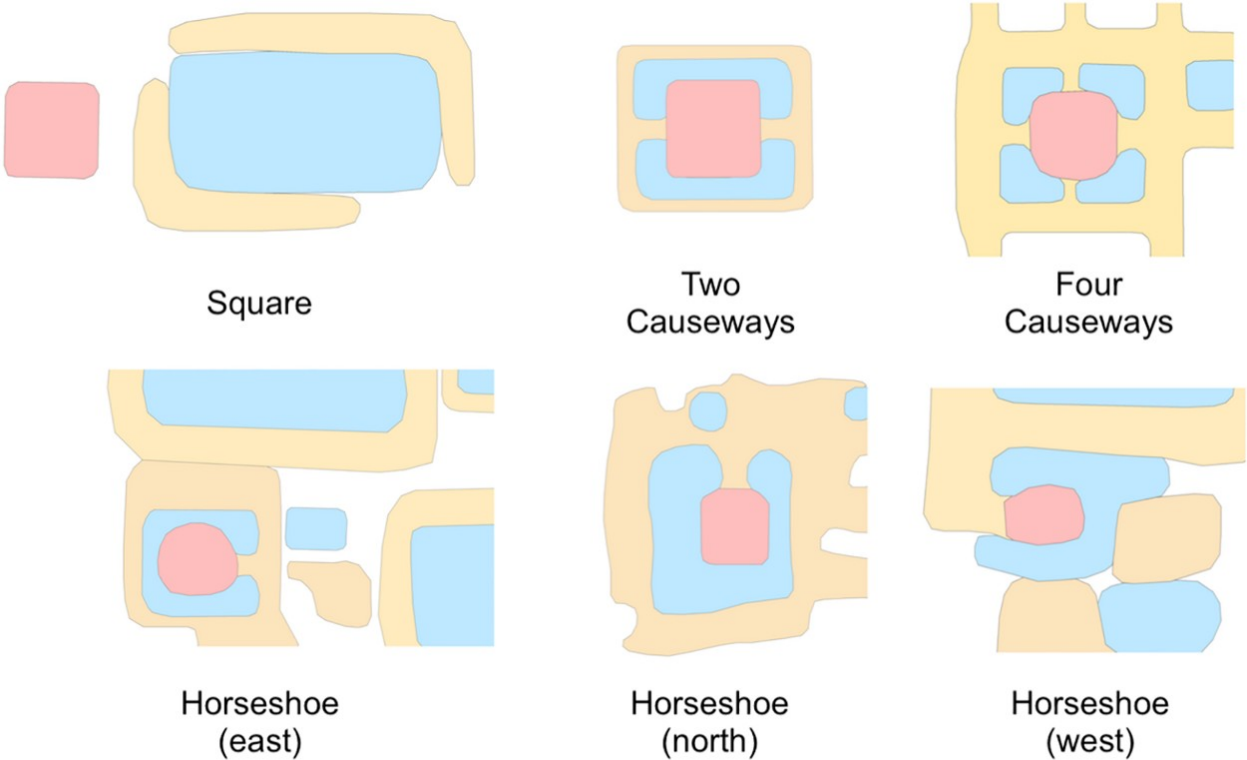
Examples of square, two causeway, four causeway, horseshoe (west), horseshoe (east), and horseshoe (north) temple (red) mound morphology.

**Fig 9:**
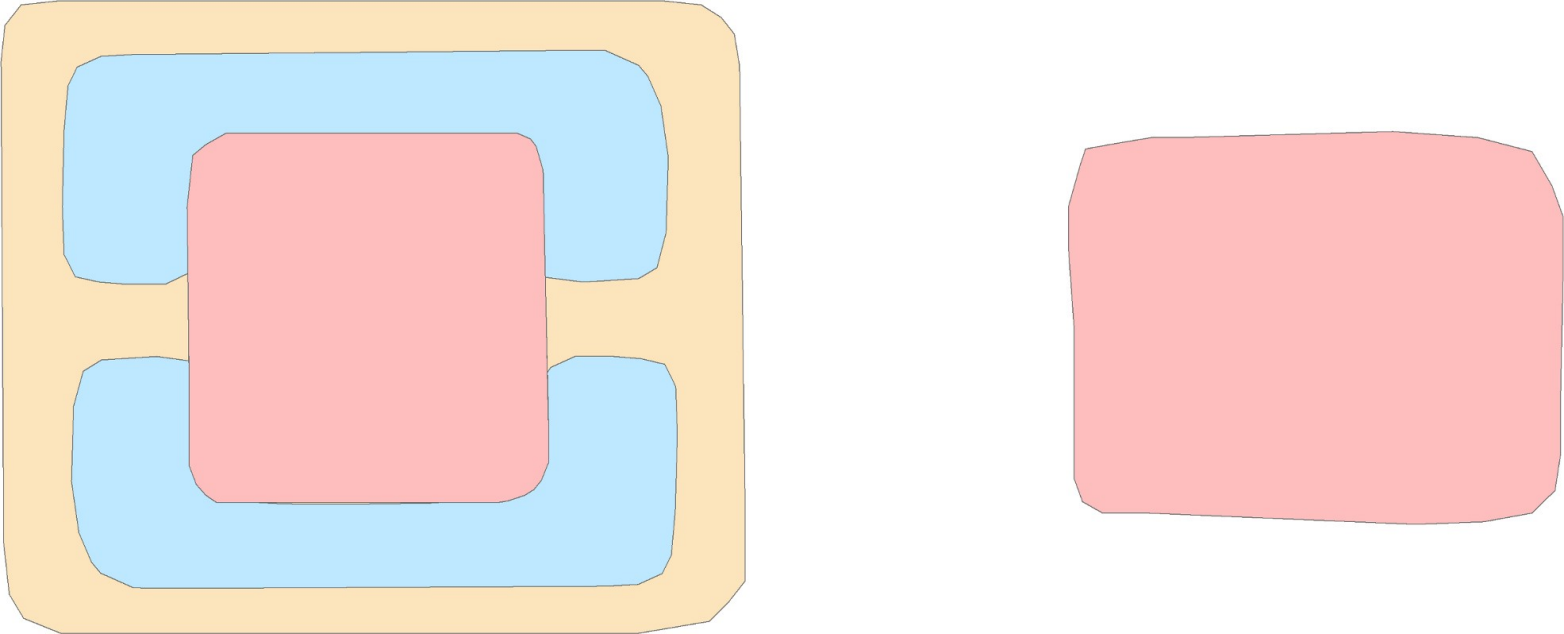
Examples of temples (red) with and without moats.

In addition to the temple dates, there are six measures of similarity, or attributes, for each temple: 1) presence or absence of a primary reservoir (coded by Klassen) (**Fig 5**); 2) Building Materials (sandstone, pink sandstone, laterite, brick, thmaphom or mountain stone) (from database created by Evans); 3) azimuth (calculated by Klassen) (**Fig 6**); 4) area (calculated by Klassen) (**Fig 7**); 5) mound morphology (square, horseshoe (east), horseshoe (west), horseshoe (northern), two causeways, four causeways, blob, and undetermined) (coded by Klassen) (**Fig 8**); 6) presence or absence of a moat (coded by Klassen) (**Fig 9**) (S2 Table). These attributes represent the current data we have on the temples. In total, there are 11 attributes because we record the presence or absence of each building material. Building materials have been recorded by dozens of archaeological surveys over the last century. The surveys were conducted for different reasons and focused on collecting different types of data; however, one of the consistent elements that was recorded was the building material of the temple. We compiled this data into a consistent framework. We then contributed to the data set by using geographic information systems analyses and remote sensing data to quantify attributes of the temples, like their size, orientation, and morphology. This was a very cost effective method of amassing great deal of information on the temples without the need for fieldwork. We reason that things like the style of temple morphology (including morphology, presence or absense of moat and primary reservoir, etc.) may reflect changes in style over time. However, while statistical analyses have been done with each feature in isolation, no chronological patterns had independently emerged from these datasets. We did not use geographic coordinates or relative spatial data as metrics of similarity in this study. Meaning, we are not auto-correlating temples based on their geographic proximity to other temples.

### Methods

To determine the foundation date of otherwise undated temples, we conducted k-means clustering, discriminant function analysis, multiple linear regression analysis, principal component analysis regression, and graph-based semi-supervised machine learning to determine if any morphological or architectural features were strong predictors of the temple dates. These analyses incorporate a variety of methods that produce either continuous-change (regression) or phase models (classification). Continuous values can be grouped into historical periods so that either technique will suffice for our purposes. Because we are interested in dividing the temples by century, each modeling approach was assessed on its ability to accurately predict the correct time period for temples with known dates. Classifications were considered satisfactory if they could successfully group temples with known dates with other temples from the same 100-year range and regressions were considered ideal if they could predict dates for temples with an AAE of 50 years or less and successful if they could predict dates for temples with an AAE of 75 years or less. For these analyses, we introduced dummy variables to represent categorical data (all attributes except azimuth). Dummy variables are independent variables that represent categorical or nominal variables and are coded to allow for statistical analyses [23].

We used a variety of initial methods including k-means clustering, discriminant function analysis and principal component analysis. These methods, however, did not yield results that allowed us to date the temples accurately (see S1 File).

#### Multiple regression analysis

Multiple linear regression analysis determines the relationship between a single dependent variable (temple date) and multiple independent variables. Linear regression is designed to perform well when there aer linear or nearly linear relationships in the data. This assumption is common in statistical modeling and holds approximately in a wide variety of applied situations [24]. Linear regression does not work well when data are grouped in clusters or when there is no clear linear relationship. Multiple linear regression is often used to identify constituent components in archaeological collections. For example, the technique has been used to determine periods of occupation from ceramic assemblages [25].

We fitted a multiple linear regression model with the all of the temple attributes. One limitation of multiple linear regression is that it cannot process temples with missing pieces of data. For example, if there is no known azimuth for a temple, the temple cannot be included in the analysis. Removing temples with missing data reduces the number of temples with known dates and complete datasets to 16. If we remove the pedestal type from the analysis, the number of temples included in the analysis increases to 73. As such, we chose to remove pedestal types from the analysis. The results from the linear model including all temple data except pedestals was statistically significant (R squared = 0.5892, adjusted R squared = 0.4883, F = 5.84, p = 0.00). The AAE in the predicted values from a leave-one-out cross evaluation is 60 years. Unfortunately, this method requires complete datasets. Much of our data is incomplete in the elements that were recorded during pedestrian survey or mapped using remotely sensed data. As such, we could only use the model to predict dates for approximately half of the sample (755 of 1437 temples).

#### Graph-based SSL (GSSL)

Graph-based SSL (GSSL) constructs a graph from training data to understand the underlying structure and relationships in the data [11]. A graph is a collection of mathematical objects with vertices connected by edges. In GSSL models, each vertex is a labeled or unlabeled data point in the training dataset. The number of vertices in the graph is determined by the total number of data points, and the number of edges is at most the square of the number of data points. The weights of the edges are determined by the amount of similarity between the two data points. In general, graph-based approaches do not extend predictions to data beyond the sample used in the graph [10].

GSSL works best when the labels between data points vary smoothly across the graph and when data points with large edge weight have the same or similar labels [11] and have the same distribution [26]. Similarly, GSSL is expected to underperform for data at either end of the range because the procedure attempts to intelligently “average” the known labels in the dataset. As a result, the procedure will never assign a date outside the range of the dates present in the original labeled set. Hence, if we remove the earliest or latest temple from the sample, it is impossible for it to be assigned the correct label in a k-fold hold out procedure (for further information, see S1 File).

## Results: Combining the results of multiple linear regression and Graph-based semi-supervised machine learning (GSSL)

In this paper, we explore several statistical approaches that fall under supervised or unsupervised paradigms. In the case-study, there are 1332 undated temples (non-labeled data points) and 105 dated temples (labeled data points). Seriation like k-means clustering is unsupervised and uses data from all the temples but does not incorporate the known dates in the analysis. In contrast, MLR is supervised and uses the known dates to determine the algorithm, but is limited to approximately 10% of the dataset and could only predict dates for approximately half of the dataset [6]. As a result, none of the analyses took full advantage of the dataset using information from both the labeled and unlabeled data to improve the algorithms. Since collecting data for all the undated temples, using excavation and traditional dating methods, would be prohibitively costly and time-consuming, a semi-supervised paradigm was a natural approach for our analysis to predict dates for the remaining temples that could not be dated using multiple linear regression. However, the GSSL model had a higher AAE than the multiple linear regression. As a result, we decided to merge the results from the GSSL and the MLR to combine the benefits of both approaches and determine estimated errors for different types of temples.

We expect GSSL performance to be worse for temples with incomplete data and for temples very dissimilar from all other temples. To test this hypothesis, we classified temples as either “well-specified” or “under-specified.” Any temple with more than five null attributes was classified as “under-specified.” A temple was also called “under-specified” if there was no other temple with which it had a similarity of at least 10. For GSSL, “well-specified” temples had a 65-year AAE, and “under-specified” temples had a 92-year AAE. This analysis demonstrates the importance of complete datasets. We expect that the results can be improved in the future with a more-complete dataset. For the MLR, “well-specified” temples had a 60-year AAE, and “under-specified” temples had a 55-year AAE; however, dates were only predicted for 34 “under-specified” temples.

GSSL is also expected to underperform in predicting dates at either end of the range. In our sample, temples with known dates from 690–820 CE had an AAE of 137 years later than their true date from the GSSL predictions and 129 for the MLR predictions. Temples with known dates from 1150–1308 CE had an AAE of 132 years before their true date from the GSSL predictions and 92 for the MLR predictions. Temples with known dates from 821–1149 CE had an AAE of 56 years from the GSSL predictions and an AAE of 50 for the MLR predictions. In all scenarios, the MLR has lower AAE than the results of the GSSL. As a result, we chose to use the MLR predictions, where possible, and use the results from the GSSL for the remainder of the analysis (S3 Table).

**Fig 10:**
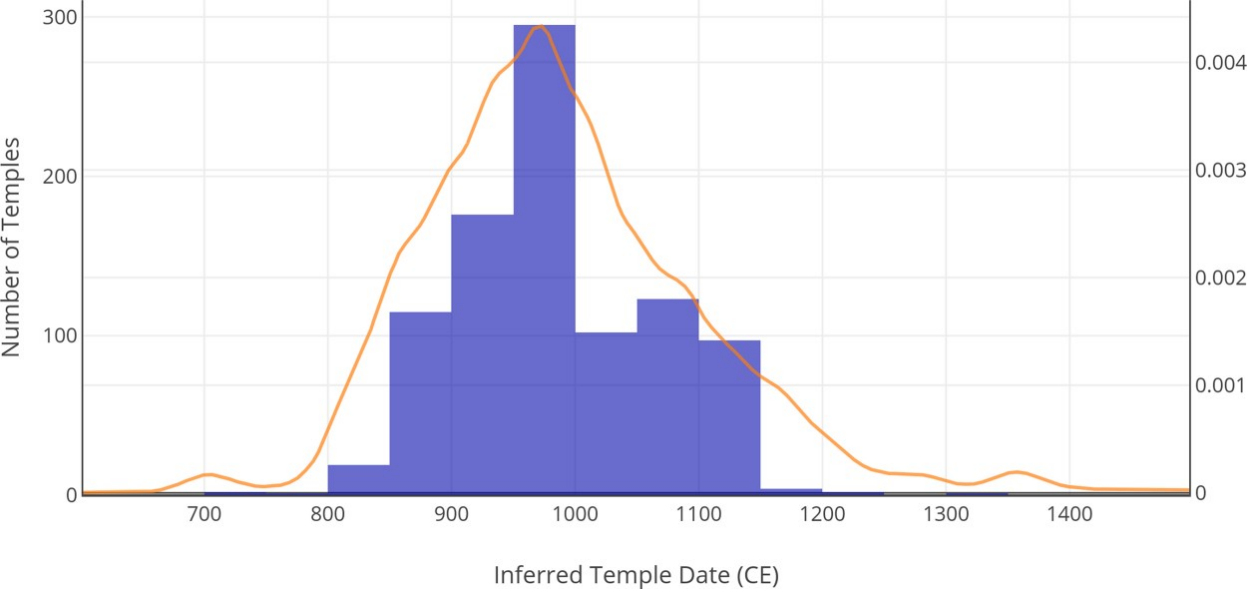
Calibrated curve assuming a normal distribution, using bchron in R, and histogram of temple dates. By calibrating the dates, we are able to increase the chance of having the correct values in our dates, but it decreases the precision of the dates. The aggregate is represented here.

In Fig 10, we plotted the results from the analysis using the bchron tool in R used for calibrating radiocarbon dates using a normal calibration curve to account for the estimated absolute error. We plotted the results as follows for the GSSL dates: “well-specified” temples between 821–1149 CE, 49 years AAE; “well-specified” temples before 820 or after 1150 CE, 130 years AAE; “under-specified” temples between 821–1149 CE, 66 years AAE; “under-specified” temples before 820 or after 1150 CE, 139 years AAE. We plotted the results as follows for the MLR dates: “well-specified” temples between 821–1149 CE, 49 years AAE; “well-specified” temples before 820 or after 1150 CE, 107 years AAE; “under-specified” temples between 821–1149 CE, 57 years AAE; “under-specified” temples before 820 or after 1150 CE, 50 years AAE. Notably, the GSSL and MLR have the same AAE for “well-specified” temples between 821–1149 CE. For temples with known dates, we used the true date, rather than the inferred date, and included multiple occupation periods if there were separated by at least 20 years.

The results of the analysis suggest an increase in the number of temples founded until 11^th^ century CE. After this, there is a decline in the number of new constructions through the 12^th^ century CE and very few subsequent temple foundations. This pattern is noted with the disclaimer that GSSL methods tend to replicate the distribution that exists in the originally labeled dataset since it replicates the distribution of the original dataset to propagate known labels to the unknown set. It is possible that we are underestimating the number of temples in the first and last periods if the original set of labels also underestimated the proportion of temples from those time periods. We argue it is unlikely we are underestimating the number of temples with inscriptions for each period in our labeled dataset. We base this argument on the assumption that most of the inscriptions from Khmer temples have been identified and inventoried, representing an accurate distribution. Within our dataset, there were 12 temples with inscriptions from multiple periods. In six of these instances, there was one date from the period 821–1149 CE that was not used in favor of an earlier or later date. Only two dates before 821 CE were not used in the model in favor of a later date and only three dates after 1149 CE were not used in the model in favor of an earlier date. One limitation of our study is that Polkinghorne’s database is constrained to lintels dating from before 1100 CE. As such, it is possible that temples that were dated by their lintels do not represent the entire distribution of temples across the landscape because of sampling bias in the original study.

## Discussion and conclusion

In the absence of detailed chronological models, the working assumption has always been that essentially all of the temples we see on the landscape were operational at the pinnacle of Angkor’s development in the eleventh century, and the lack of chronological resolution has been a persistent obstacle to complex diachronic studies of social and environmental processes. By combining the results of GSSL and MLR, we were able to predict dates for otherwise undated temples from 821–1149 CE with a 49-66-year AAE. These data can be used to create historical models of urban development at Angkor by assigning dates to temples and other landscape features that are associated with the temples. These maps can then be used in future for diachronic analyses of human-environmental and urban dynamics in the Khmer world.

SSL is becoming a large research field yet is scarcely utilized by archaeologists. Archaeologists have begun using supervised and unsupervised machine learning approaches to classify archaeological soils [27], classify artifacts [28–31], and identify archaeological features from remotely sensed data [32], but there are few examples of archaeologists using the semi-supervised paradigm. There are frequently disciplinary, cultural or knowledge-based barriers to the timely uptake of quantitative methods in archaeology, particularly when these involve some degree of automation in statistical analyses of massive datasets. For example, in the mid-1990s, Hare and Smith lamented archaeologists’ reluctant uptake of quantitative seriation methods since the introduction of computers in the 1960s [33].

The natural application of SSL to archaeological datasets has been recognized by those in machine learning communities [34, 35]. For example, archaeologists are highly interested in dates; however, C14 and OSL samples are expensive to collect and test. In contrast, sites can be identified and mapped through aerial imagery with much less effort and financial support than is required for excavation and survey. In one study [35], SSL was used to classify a collection of over 51, 000 administrative documents from the Dynasty of Ur in the 21^st^ century BCE to determine which documents related to the water transport system. The authors used identified words relating to water transport (ship, boat, haul, river, and barge) and sorted the documents using a 2-way SSL clustering algorithm. The authors then dated the documents using a supervised learning Support Vector Machine (SVM) classifier based on kingdom era. Through this study, the authors determined which kingdom eras had the most documents related to water transportation. The authors conducted the study without collaboration with domain experts. In their conclusion, the authors highlight the value of data mining and machine learning in historical document analysis.

This analysis demonstrates the utility of GSSL for anthropological inquiry and allows archaeologists to streamline data collection methods and infer information using entire datasets, including labeled and unlabeled data, as well as make predictions for underspecified data. This analysis is also an extensible base for further input of new data; as we continue to contribute new data to the complex relational databases of archaeological features, the model will continue to improve in accuracy.

Given the nature of archaeological data, it is often difficult or expensive to get “labels,” for things like artifact typologies and site chronologies. While labeled datasets can be hard to obtain new methods of data collection and the very large scale of archaeological features are now often prohibitively large to rely on subjective manual classifications and traditional archaeological methods. Similarly, it is not realistic to excavate the tens of thousands of ponds, occupation mounds, and temples that we have identified using remotely sensed data in the greater Angkor region. Given these limitations of archaeological data and inquiry, we endeavor here to make a contribution to the growing body of literature which explores the potential of semi-supervised routines and statistical inferences for archaeological inquiry.

## Supporting information

S1 File Additional details on the methodology and results for the various statistical techniques. (DOCX)

S1 Table Known dates for temples. (PDF)

S2 Table Attribute data for temples. (PDF)

S3 Table Predicted dates for temples. (PDF)

## References

[1] Evans D., et al, A comprehensive archaeological map of the world's largest preindustrial settlement complex at Angkor, Cambodia. Proceedings of the National Academy of Sciences of the United States of America, 2007 104(36): p. 14277–14282. 10.1073/pnas.0702525104 17717084 PMC1964867

[2] Evans D., Airborne laser scanning as a method for exploring long-term socio-ecological dynamics in Cambodia. Journal of Archaeological Science, 2016 74: p. 164–175.

[3] Evans D., et al, Uncovering archaeological landscapes at Angkor using lidar. Proc Natl Acad Sci U S A, 2013 110(31): p. 12595–600. 10.1073/pnas.1306539110 23847206 PMC3732978

[4] Salazar A., On Statistical Pattern Recognition in Independent Component Analysis Mixture Modelling Vol. Volume 4 of Springer Theses. 2012: Springer Science & Business Media.

[5] de Sa V. *Learning classification with unlabeled data*. in *Advances in Neural Information Processing Systems (NIPS)*. 1993.

[6] Alpaydin E., Introduction to Machine Learning 3 ed, ed. Alpaydin E. 2014, Cambridge, Mass: MIT Press.

[7] Chapelle O., Schölkopf B., and Zien A., Semi-Supervised Learning, ed. Chapelle O., Schölkopf B., and Zien A. 2010, Cambridge, Mass: MIT Press.

[8] Zhu, X., Z. Ghahramani, and J. Lafferty. Semi-Supervised Learning Using Gaussian Fields and Harmonic Functions. in Twentieth International Conference on Machine Learning. 2003. Washington, DC.

[9] Blum, A. and T. Mitchell, Combining Labeled and unlabeled data with co-training, in COLT: Proceedings of the Workshop on Computational Learning Theory, M.A. Fulk, Editor. 1998.

[10] Belkin M., Niyogi P., and Sindhwani V., Manifold regularization: A geometric framework for learning from labeled and unlabeled examples. Journal of Machine Learning Research, 2006 7: p. 2399–2434.

[11] Zhu X. and Goldberg A.B., Introduction to Semi-Supervised Learning, Zhu X. and Goldberg A.B., Editors. 2009, Morgan & Claypool.

[12] Dougherty C., *Google Photos Mistakenly Labels Black People 'Gorillas'* in *The New York Times*. 2015: New York.

[13] Hastie T., Tibshirani R., and Friedman J., The Elements of Statistical Learning: Data Mining, Inference, and Prediction 2nd ed Springer Series in Statistics. 2009: Springer.

[14] Evans D. and Fletcher R., The landscape of Angkor Wat redefined. Antiquity, 2015 89(348): p. 1402–1419.

[15] Pottier C., *Carte Archéologique de la Région d'Angkor*. *Zone Sud*, in *UFR Orient et Monde Arabe* 1999, Université Paris III—Sorbonne Nouvelle.

[16] Pottier C. and Bolle A., Le Prasat Trapeang Phong à Hariharâlaya: histoire d’un temple et archéologie d’un site. Aséanie, 2009 24: p. 61–90.

[17] Polkinghorne M., Makers and Models: Decorative Lintels of Khmer Temples, 7th to 11th Centuries, in Department of Art History and Theory Department of Archaeology 2007, University of Sydney: Sydney.

[18] Lustig E., Power and Pragmatism in the Political Economy of Angkor, in Department of Archaeology 2009, University of Sydney: Sydney.

[19] Bâty P., *Extension de l'aéroport de Siem Reap 2004: Rapport de fouille archéologique* 2005, APSARA—INRAP: Siem Reap—Paris.

[20] Lustig E., Evans D., and Richards N., Words across Space and Time: An Analysis of Lexical Items in Khmer Inscriptions, Sixth–Fourteenth Centuries CE. Journal of Southeast Asian Studies, 2007 38(1): p. 1–26.

[21] Coedès, G., Inscriptions du Cambodge. Collection de textes et documents sur l'Indochine. E. d. Boccard. Vol. I-VIII. 1937–1966, Paris: École française d'Extrême-Orient.

[22] Coedès G., Inscriptions du Cambodge. Collection de textes et documents sur l'Indochine (Vol. VIII) 1966, Paris: École française d'Extrême-Orient.

[23] Hardy M.A., Regression with dummy variables 1993: Newbury Park: Sage Publications.

[24] Shennan S., Quantifying Archaeology 2 ed 1997, Edinburgh, Scotland: Edinburgh University Press.

[25] Kohler T.A. and Blinman E., Solving Mixture Problems in Archaeology: Analysis of Ceramic Materials for Dating and Demographic Reconstruction. Journal of Anthropological Archaeology, 1987 6(1): p. 1–28.

[26] Tian Q., et al, A new analysis of the value of unlabeled data in semi-supervised learning for image retrieval. Proceedings of IEEE international conference on multimedia and Expo (ICME), 2004: p. 1019–1022.

[27] Oonk S. and Spijker J., A supervised machine-learning approach towards geochemical predictive modelling in archaeology. Journal of Archaeological Science, 2015 59: p. 80–88.

[28] van der Maaten L., et al, Computer vision and machine learning for archaeology. Proceedings of Computer Applications and Quantitative Methods in Archaeology, 2006: p. 112–130.

[29] Gansell A.R., et al, Stylistic clusters and the Syrian/South Syrian tradition of first-millennium BCE Levantine ivory carving: a machine learning approach. Journal of Archaeological Science, 2014 44: p. 194–205.

[30] Hörr C., Lindinger E., and Brunnett G., Machine learning based typology development in archaeology. Journal on Computing and Cultural Heritage (JOCCH), 2014 7(1): p. 2.

[31] Hörr C., Lindinger E., and Brunnett G., New paradigms for automated classification of pottery. 2009: Fak. für Informatik, TU.

[32] Traviglia A., Cowley D., and Lambers K., Finding common ground: human and computer vision in archaeological prospection. AARGnews, 2016 53(September): p. 11–24.

[33] Hare T.S. and Smith M.E., A New Postclassic Chronology for Yautepec, Morelos. Ancient Mesoamerica 1996 7: p. 281–297.

[34] Guyon, I., et al. Design and Analysis of the WCCI 2010 Active Learning Challenge. in The 2010 International Joint Conference on Neural Networks (IJCNN). 2010.

[35] Mavroeidis D., Diamantis D., and Vazirgiannis M. Using semi-supervised learning for mining sumerian administrative documents in the kingdom of the iii dynasty of ur. in *ECML/PKDD2007 Discovery Challenge*. 2007.

