# Supplementary material for: Semi-supervised machine learning approaches for predicting the chronology of archaeological sites: A case study of temples from medieval Angkor, Cambodia
Source: PLoS One. 2018 Nov 5;13(11):e0205649. doi: 10.1371/journal.pone.0205649 (PMC6218026; doi:10.1371/journal.pone.0205649)
Supplement: S1 File — (DOCX) [file pone.0205649.s001.docx]

SUPPORTING INFORMATION

#### K-means clustering

We performed the analysis with a varying number of clusters (2-6) but did not find any clusters that were chronologically distinct. For each of the five analyses (clusters 2-6), one cluster dominated the sample set with many of the temples with known dates. The k-means analysis for clusters (2-4) did not have any temples with dates outside of the dominant cluster. We compared the clusters against the known dates of temples, and the dates between clusters (5 and 6) did not differ significantly (ANOVA ps corrected > 0.7 and 0.98) (S1 Fig).

#### Discriminant function analysis

We next attempted seriation using discriminant analysis to group data into discrete classes. Seriation using discriminant function analysis is differentiated from k-means clustering because of the presupposition in seriation that there is a fixed number of groups based on one criterion. For this analysis, data are allocated into the most appropriate groups based the criterion and then assessed to determine if another independent criterion in the set of variables is also effective in predicting group membership [33]. This method has been used in association with Bayesian analysis to develop chronologies for ceramic assemblages [34].

Unlike most archaeological samples that have multiple lines of chronological evidence, the only known chronological information for our analysis are the dates of a select group of temples. Using known dates, we defined three clusters: before 889 CE, 889-1164 CE, and after 1165 CE. These three-time periods were chosen based on three notable kings of Angkor: before Yasovarman, before Suryavarman II, and after Suryavarman II. The model was then fitted with all the temples with known dates to predict the clusters of the rest using Latent Dirichlet allocation (LDA).

Some archaeological studies, with additional chronological information like provenienced C14 dates, have used Bayesian modeling to cross-validate and assign absolute dates to resulting clusters. Bayesian modeling is well suited to archaeological studies of chronology because they can incorporate known factors, probability curves, and contextual information into a single probability curve [34]. Because we used the known temple dates to form the initial clusters, we have no secondary chronological information remaining to cross-validate the results. Instead, we used k-fold cross-validation. K-fold cross-validation splits the labeled data into *K* equal-sized parts and withholds the *k*th part of the labeled data from the analysis. In doing so, a portion of the data is used to fit the model, and a different portion of the data is used to test it. When *k* = *n,* the cross-validation withholds one labeled data point from the learning procedure and tries to infer its label from the rest of the sample. This procedure is known as *leave-one-out* cross-validation. Leave-one-out is more precise for prediction error; however, it has high variance and is more computationally expensive because it requires running the analysis *n* times. When running *n* analyses is too computationally burdensome, and a lower variance is preferred, higher *k* values are chosen [13]. Using leave-one-out cross-validation, we determined that only 35.2% of the cross-validated grouped cases were correctly classified by the model. This suggests that discriminant function analysis is not a very reliable method for accurately dating unknown temples to our five time periods (S2 and S3 Fig).

#### Principal component analysis

we next tried principal component analysis (PCA). The goal of PCA is to simplify the data matrix, by reducing dimensionality, to identify inter-relationships among variables. PCA defines uncorrelated axes of variability (components) and evaluates the correlation between the original variables and the components. Each coordinate and group is given a “score” that can be used to assign coordinates to groups. PCA works best with interval level data with a normal distribution and few outliers [33]. PCA can be used as a preliminary methodology to decrease collinearity and replace mutually unrelated factors with mutually correlated predictors for subsequent regression.

To determine the sampling adequacy for the overall data set, we first conducted a Kaiser-Meyer-Olkin (KMO) test. Unfortunately, the overall KMO for the dataset was 0.41, which means that it was unacceptable for PCA analysis. However, there were two groups of correlated variables (group one: laterite, horseshoe mound (east), pedestal type A3, moat, and square; group two: Pedestal types A1, A2, and A4). We re-ran the PCA analysis for each group independently, which had an overall KMO of .645 for group one and .369 for group two. Based on the KMO scores, we decided to proceed with group one and conducted Bartlett’s test of sphericity to ensure that there are no correlations between variables. The Bartlett’s test was statistically significant (p < 0.00), indicating that the data was likely factorable. The analysis revealed three components that explained 49.3%, 22.7%, and 17.1% of the total variance, respectively. We then calculated a Pearson correlation coefficient for the year and three components. The first component (PCC = 0.278, p-value = 0.004) and the second component (PCC = 0.314, p-value = 0.001) were statistically significant; however, the third component was not statistically significant (0.168, p-value = 0.09).

We then fitted a multiple linear regression model with various combinations of the three components. The linear models were statistically significant (p < 0.001) for all combinations of the three components except for the second component alone (p = 0.090). The linear model using all three of the components explained 20.4% of the variance (R = 0.452, adjusted R squared =0.181, F=8.6, p= 0.000), the first and second components explained 17.6% of the variance (R = 0.420, adjusted R squared = 0.160, F =10.9, p = 0.000), the first component alone explained 7.7% of the variance (R = 0.278, adjusted R squared =0.068, F=8.6, p= 0.004), and the second component alone explained 9.9% of the variance (R = 0.314, adjusted R squared =0.090, F=11.3, p= 0.001). Unfortunately, the PCA did not explain more of the variance than the multiple linear regression.

**Graph-based Semi-Supervised Machine learning.** GSSL models propagate labels to unlabeled data based on edge weights; the larger the edge weight the more similar the data points. First, a measure of similarity is defined between data points. One typical example is the Hamming distance, which measures the difference between data points by the number of attributes on which they differ [36]. These similarity measures are then converted to edge weights; often, this is accomplished via a Gaussian kernel, which puts significantly more weight on edges connecting data points which are very similar [8]. Finally, labels are assigned to vertices to minimize the total penalty arising from a mathematical object known as the “graph Laplacian.” This penalty is similar to the least-squares formula used in linear regression; however, it replaces the assumption of linearity by a more flexible assumption on the so-called “manifold structure” of the data set [37]. To define the edge weights, many GSSL methods use a Gaussian kernel applied to the Hamming distance [8]. GSSL methods are quire flexible and can be used for both binary or multi-way classification [38].

We conducted the analysis using NumPy, a scientific computing package for Python. We first sanitized the data by standardizing the data to remove errors caused by human data collection, so the data was suitable for computer analysis. After collection and sanitization, the data was loaded into an SQLite database and manipulated via the Python SQLAlchemy package. We first normalized numeric data (azimuth and area), so each entry lay between 0 and 1. We then calculated the similarity between data points by using the Hamming distance. Explicitly, the similarity between two temples was defined as the number of non-numeric fields on which they agreed plus 2, minus the distance between the two temples’ normalized azimuth and area fields ((azimuth_1_ - azimuth_2_)^2^ + (area_1_ - area_2_)^2^). If either temple was missing azimuth or area data, the corresponding entry for that temple was replaced by .5. With this definition, the similarity for each pair of temples lay between 0 and 11. Using these results, we built a weighted graph with edge weights assigned via a Gaussian kernel to put progressively greater weight on objects that are closer. Finally, we assigned years to unlabeled temples to minimize total penalty arising from graph Laplacian.

To cross-validate our results, we used a standard k-fold leave-one-out validation, as described in the discriminant function analysis section. We conducted the procedure in a combination of Python and Bash where *k* = *n* [13]: for each temple for which we know the true date, we removed its label and tried to infer it from all the other labels. We repeated the process 105 times, once for every labeled temple in our dataset. We chose to use *k* = *n* because it has a lower bias than some lower values of *k*, even though the variance is higher. The cross-validation suggests that our AAE for the entire dataset is 74 years from the original label (median absolute error is 50 years).

To test whether the labeled and non-labeled temples are from the same distribution, we compared temples with inscriptions to temples with lintels but no inscriptions in our labeled dataset. If the labeled and unlabeled temples do represent different distributions, it could undermine the effectiveness of the GSSL model [39]. Temples with inscriptions are often fundamentally different from temples without inscriptions. Inscriptions were expensive to commission and, as such, were often written for and by the elite [20]. We argue that temples without inscriptions that were dated by their lintels are more likely to represent the non-royal and non-elite temples. There were 35 labeled temples whos dates were derived from lintels alone because they did not have inscriptions; AAE for these temples is 54-years, which suggests that the GSSL works better for them than it did for the entire sample

# SUPPORTING Figures


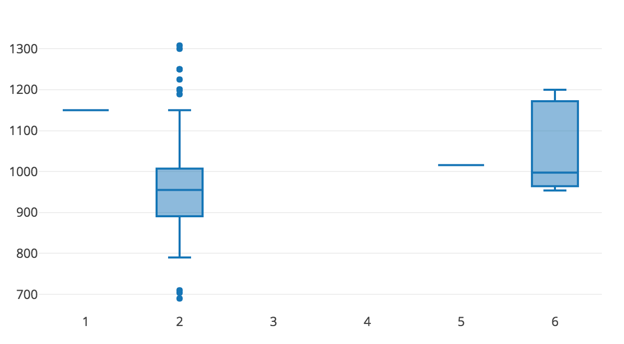

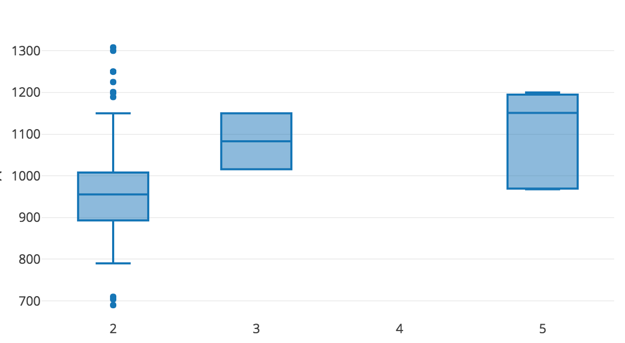
S1 Fig. Box plots of four K-means clusters for five and six clusters

.


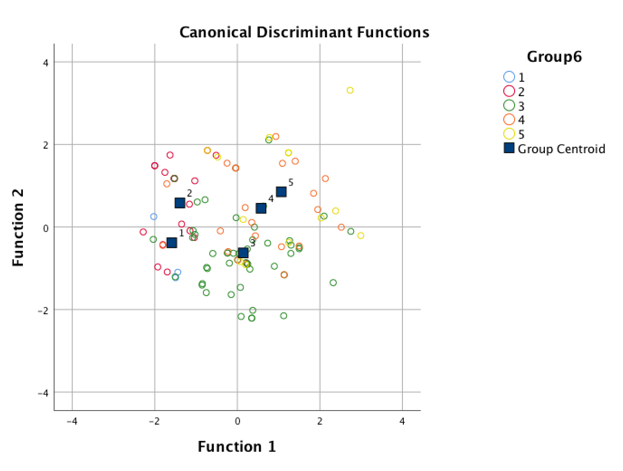


S2 Fig. Canonical discriminant functions of temples for five-time periods at Angkor

.


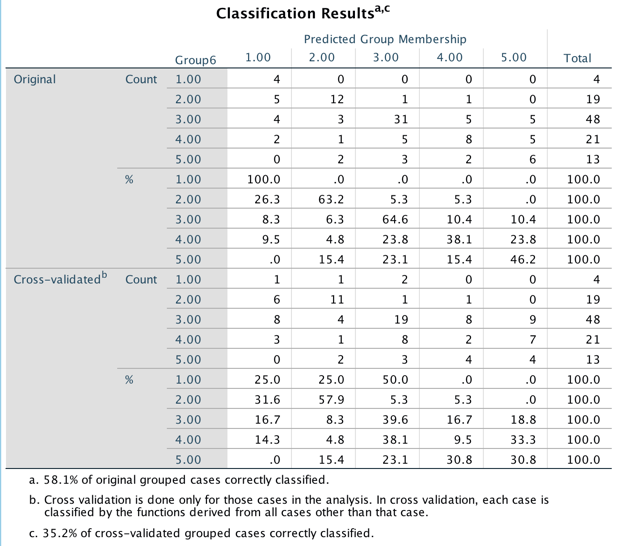


S3 Fig. Results from the discriminant function analysis with leave-one-out cross-validation

.

Groups represent the following time periods ordered from 1 through 5, pre-802 CE, 803-889 CE, 890 – 1001 CE, 1002 – 1164 CE, and 1165 – 1320 CE.
