# Supplementary material for: Semi-supervised machine learning approaches for predicting the chronology of archaeological sites: A case study of temples from medieval Angkor, Cambodia
Source: PLoS One. 2018 Nov 5;13(11):e0205649. doi: 10.1371/journal.pone.0205649 (PMC6218026; doi:10.1371/journal.pone.0205649)
Supplement: S1 Table — (PDF) [file pone.0205649.s002.pdf]

id	name_english	lustig_site_id	archsite_id	pelle_objectid	Eileen_median_inscription_date(1)	certainty
3	Damnak Sdach	518	63200	27	949.5	Uncertain
61	Wat Thipadei	520	63600	1145	912	Certain
63	Preah Enkosei (Pr.)	53	54400	1171	975.5	Certain
92	Phum Khcay	552	65201	1899	1299.5	Uncertain
112	Kouk Svay Chek	616	200429	2779	1308	Certain
156	Samrong	51	61002	3790	1090	Certain
173	Kouk Pongrô (Pr.)	287	52002	4157	899.5	Uncertain
176	Andaung Kouk Ponlei	null	100131	4173		
178	Sralao (Pr.)	null	60900	4178		
186	Kouk Kandal	null	62000	4268		
188	Kouk Kuk	null	60600	4285		
191	Char (Pr.)	50	59800	4311	986.5	Certain
257	Kouk Châk (Pr.)	502	54401	6137	1075	Uncertain
260	He Phka (Pr.)	500	54406	6150	956	Uncertain
262	Trapeang Totung Thngay (Pr.)	null	57800	6168		
263	Kouk Svay Pream	583	57900	6179	969	Certain
264	Kouk Daung	544	58000	6184	1002	Certain
265	Trapeang Phong (Pr.)	195	58300	6204	983	Certain
288	Kouk Neak Ta Trapeang Péa	null	100352	6839		
296	Kouk Ta Ro	null	51300	6890		
298	Phnom Bok (Pr.)	null	54700	6901		
299	Trapeang Chambâk (Pr.)	null	54800	6903		
300	Leak Neang (Pr.)	658	54900	6908	909	Certain
304	Daun So	504	58903	6928	1075	Uncertain
307	Kouk Pô (Pr.)	586	59700	6944	978	Certain
320	Kouk Neak Ta	338	200218	7078	899.5	Uncertain
335	Phum Prasat	null	56800	7290		

id	name_english	lustig_site_id	archsite_id	pelle_objectid	Eileen_median_inscription_date(1)	certainty
338	Phnom Phu (Pr.)	null	57100	7388		
386	Cedei (Pr.)	null	50200	8424		
393	Wat Athvea	737	50000	8463	1149.5	Uncertain
401	Kouk O Chrung	503	54600	8524	1007.5	Certain
447	Kouk Sla Ket (Pr.)	538	51400	8829	956	Uncertain
472	Lolei (Pr.)	174	58900	8941	889	certain
473	Kandol Dom North (Pr.)	126	58600	8948	882.5	Uncertain
474	Kandol Dom South (Pr.)	741	58700	8954	949.5	Uncertain
475	Olok (Pr.)	343	58402	8958	883	Uncertain
479	Preah Kô (Pr.)	167	58500	8991	889	certain
484	Kouk Thvèng	231	100608	9034	947	Certain
502	Phnom Krom (Pr.)	null	50100	9167		
523	Kouk Ta Nei	null	100472	9389		
525	Prei Monti (Pr.)	753	58200	9414	849.5	Uncertain
543	Koh Ho (Pr.)	733	51602	9674	1049.5	Uncertain
549	Kouk Trapeang Lopov (Pr.)	220	51800	9717	949.5	Uncertain
603	Chau Srei Vibol (Pr.)	null	56400	10285		
643	Wat Kralanh	null	63500	11403		
669	Tor (Pr.)	585	54300	12101	1189	Certain
686	Kouk Daung	null	60200	13015		
721	Banteay Thom (Pr.)	651	52000	14233	1200.5	Uncertain
759	Banteay Srei (Pr.)	97	54602	15897	1301	Uncertain
761	Phnom Dei II	618	61301	15929	893	Certain
778	Sek Ta Tuy (Pr.)	103	21302	16745	1035	uncertain
788	Bayon (Pr.)	84	47400	17264	1200.5	Uncertain
789	Phnom Roung	null	59600	17367		
795	Thommanon (Pr.)	null	49000	17869		

id	name_english	lustig_site_id	archsite_id	pelle_objectid	Eileen_median_inscription_date(1)	certainty
796	Ta Keo (Pr.)	59	53300	18002	1025.5	Uncertain
800	Chau Say Tevoda	null	48900	18102		
806	Kutishvara	null	53502	19104		
807	Bat Chum (Pr.)	692	53600	19128	956.5	Certain
814	Kravan (Pr.)	57	53700	19257	921	Certain
815	East Mebon	364	53100	19322	953	Certain
818	Pre Rup (Pr.)	581	53800	19747	1080	Certain
821	Banteay Samre (Pr.)	null	54100	19872		
823	To Be Determined	499	100885	20134	949.5	Uncertain
827	West Mebon	136	51700	20719	713	Certain
831	Prei Khmeng (Pr.)	124	59400	20916	924.5	Certain
832	Ak Yum (Pr.)	122	59202	20989	674	Uncertain
862	Kapilapura	512	49200	22798	968	certain
875	Bei (Pr.)	null	49400	23532		
876	Bay Kaek West (Pr.)	null	49300	23533		
885	Phnom Bakheng (Pr.)	110	49600	23569	968	Certain
927	temple east of Khleang, north	null	48006	24008		
941	Top (Pr.)	null	48600	25094		
1002	Prei Prasat	666	53002	26643	902	Uncertain
1003	Komnâp (Pr.)	657	53003	26644	899.5	Uncertain
1004	Leak Neang (Pr.)	54	54000	26645	959	Certain
1031	Wat Prasat	null	59100	26679		
1047	Wat Khnat	214	59200	26700	851	Certain
1058	Bakong (Pr.)	434	58400	26716	881	Certain
1061	Krohom (Pr.)	null	55500	27057		
1063	Phnom Sruoch	553	55815	27133	1149.5	Uncertain
1065	Khting Slap (Pr.)	null	55806	27212		

id	name_english	lustig_site_id	archsite_id	pelle_objectid	Eileen_median_inscription_date(1)	certainty
1083	Don Meas (Pr.)	null	55611	28073		
1108	Pr. Ang Ampil	206	200764	null	1002	Certain
1121	O Paong (Pr.)	null	55600	null		
1122	Damrei Krap (Pr.)	null	55800	null		
1125	O Ka-aek (Pr.)	498	58907	null	949.5	Certain
1143	Chrung (Pr,), northeast	511	47203	null	1249.5	Uncertain
1144	Chrung (Pr.), northwest	508	47204	null	1249.5	Uncertain
1146	Anlong Thom (Pr.)	null	55403	null		
1147	Koki (Pr.)	null	55603	null		
1148	Rup Arak (Pr.)	null	55604	null		
1152	Thmâ Puok (Stèle)	516	81200	null	989	Certain
1153	Banteay Chmar	514	81600	null	1200.5	Uncertain
1155	Wat Tasar Sdâm	129	100011	null	899.5	Uncertain
1219	Bos Neak (Pr.)	null	55610	null		
1260	Thm Dap (Pr.)	null	55702	null		
1262	Neak Ta (Pr.)	null	55900	null		
1613	Ta Prohm	558	53400	12151	1186	Certain
1618	Neak Pean (Pr.)	715	52700	null	1249.5	Uncertain
1640	Royal Palace	null	47601	null		
1662	Angkor Wat	490	49700	22446	1149.5	Uncertain
1665	Preah Khan		52200			
1668	Wat Damnak	77	54500	null	1025.5	Uncertain
1721	Trapeang Srangè (Pr.)	null	58800	null		

id	Eileen_median_inscription_date(2)	certainty	Eileen_median_inscription_date(3)	certainty	Eileen_median_inscription_date(4)	certainty	Eileen_median_inscription_date(5)	certainty
3								
61								
63	972.5	Certain						
92								
112								
156								
173								
176								
178								
186								
188								
191								
257								
260								
262								
263								
264								
265								
288								
296								
298								
299								
300								
304								
307	1050	Certain						
320								
335								

id	Eileen_median_inscription_date(2)	certainty	Eileen_median_inscription_date(3)	certainty	Eileen_median_inscription_date(4)	certainty	Eileen_median_inscription_date(5)	certainty
338								
386								
393								
401								
447								
472	891	Certain	893	Certain	949.5	certain		
473								
474								
475								
479	878	Uncertain	879	Certain	886	Certain	891	Certain
484								
502								
523								
525								
543								
549	1002	Certain						
603								
643								
669								
686								
721	1249.5	Uncertain						
759	1305	Certain	969	Certain	949.5	Uncertain	1011	Certain
761								
778	1030	uncertain	980	uncertain	980	uncertain		
788	1249.5	Uncertain	1327	Certain				
789								
795								

id	Eileen_median_inscription_date(2)	certainty	Eileen_median_inscription_date(3)	certainty	Eileen_median_inscription_date(4)	certainty	Eileen_median_inscription_date(5)	certainty
796	1043.5	Uncertain	1004.5	Certain	893	Certain	1238	Uncertain
800								
806								
807	953	Uncertain						
814								
815	956	Uncertain						
818	961	Certain						
821								
823	956	Uncertain						
827								
831								
832	704	Certain	1001	Certain				
862								
875								
876								
885	649.5	Uncertain	999.5	Uncertain	1049.5	Uncertain		
927								
941								
1002								
1003								
1004								
1031								
1047								
1058	887	Uncertain	899.5	Uncertain	949.5	Uncertain	949.5	Uncertain
1061								
1063								
1065								

id	Eileen_median_inscription_date(2)	certainty	Eileen_median_inscription_date(3)	certainty	Eileen_median_inscription_date(4)	certainty	Eileen_median_inscription_date(5)	certainty
1083								
1108								
1121								
1122								
1125								
1143								
1144								
1146								
1147								
1148								
1152								
1153								
1155								
1219								
1260								
1262								
1613	1200.5	Uncertain						
1618								
1640								
1662	1249.5	Uncertain						
1665								
1668								
1721								

id	Eileen_median_inscription_date(6)	certainty	Eileen_median_inscription_date(7)	certainty	Eileen_median_inscription_date(8)	certainty	lintel_median	lintel_date_certainty	Date to use
3							null	null	949.5
61							975	0	943
63							961	1	968
92							null	null	1299.5
112							null	null	1308
156							null	null	1090
173							null	null	899.5
176							790	0	790
178							1071	1	1071
186							970	0	970
188							980	0	980
191							980	0	983
257							880	0	880
260							null	null	956
262							960	0	960
263							870	0	969
264							null	null	1002
265							865	0	983
288							null	null	1199
296							960	0	960
298							895	0	895
299							895	0	895
300							900	0	905
304							null	null	1075
307							1004	1	1004
320							null	null	899.5
335							905	0	905

id	Eileen_median_inscription_date(6)	certainty	Eileen_median_inscription_date(7)	certainty	Eileen_median_inscription_date(8)	certainty	lintel_median	lintel_date_certainty	Date to use
338							915	0	915
386							970	0	970
393							null	null	1149.5
401							null	null	1007.5
447							970	0	963
472							889	1	893
473							null	null	882.5
474							null	null	949.5
475							710	0	710
479	897	Certain	999.5	Uncertain	1005	Certain	879	1	879
484							null	null	947
502							890	0	890
523							null	null	1200
525							880	0	865
543							null	null	1049.5
549							955	1	976
603							1016	1	1016
643							1040	0	1040
669							null	null	1189
686							930	0	930
721							null	null	1225
759	1049.5	Uncertain	967.5	Certain			967	1	967
761							null	null	893
778							null	null	1007.5
788							null	null	1201
789							690	0	690
795							null	null	1100

id	Eileen_median_inscription_date(6)	certainty	Eileen_median_inscription_date(7)	certainty	Eileen_median_inscription_date(8)	certainty	lintel_median	lintel_date_certainty	Date to use
796	1149.5	Uncertain	1050	uncertain			null	null	1005
800							null	null	1100
806							955	0	955
807							953	1	954
814							921	1	921
815							953	1	954
818							961	1	961
821							null	null	1150
823							null	null	953
827							null	null	713
831							650	0	925
832							690	0	704
862							875	0	968
875							900	0	900
876							995	0	995
885							895	0	900
927							1005	0	1005
941							970	0	970
1002							null	null	902
1003							null	null	899.5
1004							960	0	960
1031							970	0	970
1047							710	0	851
1058	1025.5	Uncertain					880	0	881
1061							830	0	830
1063							815	0	815
1065							820	0	820

id	Eileen_median_inscription_date(6)	certainty	Eileen_median_inscription_date(7)	certainty	Eileen_median_inscription_date(8)	certainty	lintel_median	lintel_date_certainty	Date to use
1083							840	0	840
1108							null	null	1002
1121							825	0	825
1122							810	0	810
1125							880	0	949
1143							null	null	1249.5
1144							null	null	1249.5
1146							835	0	835
1147							825	0	825
1148							825	0	825
1152							null	null	989
1153							null	null	1200.5
1155							null	null	899.5
1219							840	0	840
1260							830	0	830
1262							825	0	825
1613							null	null	1193
1618							null	null	1250
1640							1011	1	1011
1662							null	null	1150
1665									1151
1668							null	null	1025.5
1721							880	0	880

id	Rationale	literature	Additional time periods	Known dates not used
3	A			
61	C			
63	B	"Wat preah Einkosei is one of the oldest pagodas in Siem Reaap town and		
92	A			
112	A			
156	A			
173	A			
176	A			
178	A			
186	A			
188	A			
191	B			
257	F			
260	A			
262	A			
263	F			
264	A			
265	F			
288	F	guide said that a doorframe we found at the temple is typical J7 style, 11[illegible]		
296	A			
298	A	900 (Glazie, The Angkor Guide, page 49)		
299	A			
300	B			
304	A			
307	F			
320	A			
335	A			

id	Rationale	literature	Additional time periods	Known dates not used
338	A			
386	A			
393	A			
401	A			
447	B			
472	D	893 (Glazie, The Angkor		950
473	A			
474	A			
475	F			
479	F			1001
484	A			
502	A	900 (Glazie, The Angkor Guide, page 49)		
523	E	1150-1250 (Glazie, The Angkor Guide, page 49		
525	B			
543	A			
549	C			
603	A			
643	A			
669	A			
686	A			
721	B			
759	D	967 (Glazie, The Angkor		1305
761	A			
778	C			
788	D	1150-1250 (Glazie, The		1327
789	A			
795	E	1050-1150 (Glazie, The Angkor Guide, page 49)		

id	Rationale	literature	Additional time periods	Known dates not used
796	D	1000-1050 (Glazie, The		893
800	E	1050-1150 (Glazie, The Angkor Guide, page 49)		
806	A			
807	B			
814	B	921 (Glazie, The Angkor Guide, page 49)		
815	B	952 (Glazie, The Angkor Guide, page 49)		
818	D	961 (Glazie, The Angkor		1080
821	E	1150 (Glazie, The Angkor Guide, page 189)		
823	B			
827	A			
831	F			
832	F			1001
862	F			
875	A	900-999 (Glazie, The Angkor Guide, page 49)		
876	A			
885	D	900 (Glazie, The Angkor		650
927	A			
941	A			
1002	A			
1003	A			
1004	B			
1031	A			
1047	F			
1058	D	881 (Glayes		1001
1061	A			
1063	F			
1065	A			

id	Rationale	literature	Additional time periods	Known dates not used
1083	A			
1108	A			
1121	A			
1122	A			
1125	F			
1143	A			
1144	A			
1146	A			
1147	A			
1148	A			
1152	A			
1153	A			
1155	A			
1219	A			
1260	A			
1262	A			
1613	B	1186 (Glazie, The Angkor Guide, page 49); strong support for habitation d		
1618	A			
1640	A			
1662	D	1150 (Glazie, The Angkor		1150
1665	F	1151 (Glazie, The Angkor Guide, page 173)		
1668	A			
1721	A			

id	
3	
61	
63	l was built around a 10th century brick temple. Originally, it was a sanctua
92	
112	
156	
173	
176	
178	
186	
188	
191	
257	
260	
262	
263	
264	
265	
288	[illegible]9 is the middle of J7's reign
296	
298	
299	
300	
304	
307	
320	
335	

id	
1083	
1108	
1121	
1122	
1125	
1143	
1144	
1146	
1147	
1148	
1152	
1153	
1155	
1219	
1260	
1262	
1613	uring the 12-13th centuries (GAP Ta Prohm excavation report page 5); are
1618	
1640	
1662	
1665	
1668	
1721	

1639	#N/A
1640	#N/A
1662	#N/A
1665	#N/A

id

1668	#N/A
1676	#N/A
1721	#N/A

id	
3	
61	
63	ry with two enclosures that includes a group of three east-facing brick tov
92	
112	
156	
173	
176	
178	
186	
188	
191	
257	
260	
262	
263	
264	
265	
288	
296	
298	
299	
300	
304	
307	
320	
335	

id	
1083	
1108	
1121	
1122	
1125	
1143	
1144	
1146	
1147	
1148	
1152	
1153	
1155	
1219	
1260	
1262	
1613	a was occupied as early as the 10th century and as late as the 13th centur
1618	
1640	
1662	
1665	
1668	
1721	

id	
3	
61	
63	vers (each with a sandstone door frame), two libraries, an east gate/entra
92	
112	
156	
173	
176	
178	
186	
188	
191	
257	
260	
262	
263	
264	
265	
288	
296	
298	
299	
300	
304	
307	
320	
335	

id

1083
1108
1121
1122
1125
1143
1144
1146
1147
1148
1152
1153
1155
1219
1260
1262
1613 y (GAP Ta Prohm excavation report page 7)
1618
1640
1662
1665
1668
1721

id	
3	
61	
63	nce pavilion, an encircling moat, and a second laterite encloseure" (GAP \
92	
112	
156	
173	
176	
178	
186	
188	
191	
257	
260	
262	
263	
264	
265	
288	
296	
298	
299	
300	
304	
307	
320	
335	

id	
3	
61	
63	Wat Preah Einkosei preliminary report: 2); lots of occupation post-angkori[illegible]
92	
112	
156	
173	
176	
178	
186	
188	
191	
257	
260	
262	
263	
264	
265	
288	
296	
298	
299	
300	
304	
307	
320	
335	

id	
3	
61	
63	an period (GAP Wat Preah Einkosei preliminary report)
92	
112	
156	
173	
176	
178	
186	
188	
191	
257	
260	
262	
263	
264	
265	
288	
296	
298	
299	
300	
304	
307	
320	
335	
