# Supplementary material for: Semi-supervised machine learning approaches for predicting the chronology of archaeological sites: A case study of temples from medieval Angkor, Cambodia
Source: PLoS One. 2018 Nov 5;13(11):e0205649. doi: 10.1371/journal.pone.0205649 (PMC6218026; doi:10.1371/journal.pone.0205649)
Supplement: S2 Table — (PDF) [file pone.0205649.s003.pdf]

Temple ID	name_english	Lustig ID	Archsite ID	Pelle ID	Azimuth	Principle Reservoir	Sandstone	Pink Sandstone	Laterite	Brick	Thmaphnom	other	Morphology
876	Bay Kaek West (Pr.)	0	49300	23533	90.4	0	1	0	1	1	0	0	square
874	Sok Kraop (Pr.)	0	49602	23531	90.0	0	0	0	1	1	0	0	square
878	remains of a temple	0	47155	23539	90.0	0	1	0	1	1	0	0	square
933	Mangalartha (Pr.)	614	48700	24223	90.0	0	1	0	0	0	0	0	square
973	sculptures	0	47127	26613	90.7	0							square
968	terrace	0	47169	26608	89.5	0							square
965	terrace	0	47133	26605	89.9	0							square
952	Rong Damrei	0	47125	25407	83.8	0							square
956	temple remains	0	47158	26593	87.2	0							square
1015	To Be Determined	0	47102	26660	89.7	1	1	0	0	1	0	0	square
1069	Dot Sdach Kamlong	0	55202	27233	91.2	0	1	0	1	1	0	0	square
877	Bay Kaek East (Pr.)	0	47106	23534	90.0	0	1	0	1	1	0	0	square
940	Terrace I	0	47113	25089	88.9	0							square
958	Terrace R	0	47142	26595	82.4	0							square
542	Srah Khhut	0	101002	9670	###	1	0	0	1	1	0	0	square
912	Preah Pithu, Y	0	48500	23970	82.9	0	1	0	0	0	0	0	square
832	Ak Yum (Pr.)	122	59202	20989	87.2	0	0	0	0	1	0	0	square
949	terrace	660	47122	25404	90.0	0							square
989	terrace	0	47116	26629	87.7	0							square
978	terrace	0	47165	26618	90.0	0	1	0	0	0	0	0	square
302	Kroes Prei Ten	0	57302	6914	85.5	0	1	0	1	0	0	0	square
875	Bei (Pr.)	0	49400	23532	###	0	0	0	1	1	0	0	square
1618	Neak Pean (Pr.)	715	52700	13179	90.6	0	1	0	1	0	0	0	
969	terrace	0	47167	26609	89.3	0							square
635	Neak Ta Dem Chomk	0	200453	11172	83.9	0	1	0	0	0	0	0	square
976	terrace	0	47126	26616	87.9	1							square
936	Terrace B	0	47136	24627	84.6	0							square
526	To Be Determined	0	200649	9426	89.5	1	1	0	1	0	0	0	horsesho
1048	To Be Determined	0	200700	26701	90.0	0	0	0	1	0	0	0	square
685	Kouk Bos	0	200492	13014	90.0	0	1	0	1	0	0	0	square
884	To Be Determined	0	47105	23567	59.9	0	1	0	1	0	0	0	circle
1016	To Be Determined	0	200707	26661	90.0	0	0	0	1	0	0	0	square

Temple ID	name_english	Lustig ID	Archsite ID	Pelle ID	Azimuth	Principle Reservoir	Sandstone	Pink Sandstone	Laterite	Brick	Thmaphnom	other	Morphology
111	Kouk Pongro	0	62100	2697	90.0	1	1	0	1	0	1	0	horsesho
908	Terrace T	0	47134	23965	88.4	0							square
944	Terrace G	0	47140	25398	77.3	0	1	0	1	0	0	0	square
794	embarkation terrace	0	53302	17846	###	0	0	0	1	0	0	0	square
941	Top (Pr.)	0	48600	25094	87.0	0	1	0	1	0	0	0	blob
1007	Ong Mong (Pr.)	0	53004	26648	89.5	0	1	0	1	1	0	0	square
990	Terrace J	0	47111	26630	86.8	0	1	0	1	0	0	0	square
1061	Krohom (Pr.)	0	55500	27057	92.4	0	0	0	0	1	0	0	square
704	Toul Yey Reat	0	200686	13281	94.2	0	0	0	1	0	0	0	horsesho
977	temple	0	47164	26617	80.8	0	0	0	1	1	0	0	square
938	Terrace E	0	47144	24649	84.3	0	1	0	0	0	0	0	square
702	Preah Phtu	0	52300	13260	77.3	0	1	0	1	0	0	0	horsesho
883	To Be Determined	0	47104	23560	90.0	0	1	0	1	1	0	0	square
321	Tuol Anlong La-âk	0	200517	7079	73.6	0	0	0	1	1	0	0	horsesho
711	Wat Mahather	0	61501	13604	79.7	0							horsesho
1076	Preah Bat Phnom Be	0	55818	28056	###	0							square
1045	Kouk Prasat Sambuc	0	100663	26698	###	0	1	0	0	1	0	0	square
1078	Kouk Kroes	0	200737	28060	89.5	1							square
484	Kouk Thvèng	231	100608	9034	93.1	0	1	0	0	0	0	0	horsesho
985	Terrace M	794	47107	26625	87.2	0	1	0	1	0	0	0	square
723	Tonlé Snguot (Pr.)	767	52100	14266	90.0	0	1	0	1	0	0	0	square
706	Toul Ta Kong	0	200688	13286	###	1	0	0	1	0	0	0	square
960	remains	0	47159	26597	86.8	0							square
937	Terrace D	0	47143	24630	83.9	0	1	0	1	0	0	0	square
1480	Srah Ât Tœuk	0	100745	10251	89.0	0	0	0	1	0	0	0	square
1489	Ta Ponleu	0	54410	10990	88.9	1							square
161	Kouk Prasat	0	100978	3929	84.1	1							horsesho
1067	Krol Romeas (Pr.)	0	55402	27228	90.6	0							square
287	Trapeang Suot Muot	0	100372	6777	88.7	1	1	0	1	0	0	0	horsesho
582	Trapeang Bet Phon	0	200470	10152	92.7	1	0	0	1	0	0	0	horsesho
44	Kouk Kbal Domrey	0	200053	911	###	1	1	0	0	0	0	0	horsesho
999	To Be Determined	0	200644	26640	91.1	0	1	0	0	0	0	0	horsesho

Temple ID	name_english	Lustig ID	Archsite ID	Pelle ID	Azimuth	Principle Reservoir	Sandstone	Pink Sandstone	Laterite	Brick	Thmaphnom	other	Morphology
1000	Trapéang Svay Chuk	0	100393	26641	76.5	0	1	0	0	1	0	0	square
1004	Leak Neang (Pr.)	54	54000	26645	85.6	0	0	0	0	1	0	0	Square
839	Anluong Ok	0	100480	21535	83.3	1	0	0	1	0	0	0	horsesho
663	Trapéang Chrei	0	100189	11941	84.8	1	1	0	1	0	0	0	square
585	Kouk A Rak	0	200475	10162	###	0	0	0	1	0	0	0	horsesho
54	Kouk Bos Leas	0	100061	1077	88.6	0							horsesho
1002	Prei Prasat	666	53002	26643	89.7	1							square
274	Kuk Top Thom	0	21900	6519	89.4	0							square
799	Eastern Hospital Cha	772	49101	18083	89.9	0	1	0	1	0	0	0	square
675	Kouk Srok Thnot	0	100690	12344	98.7	1	1	0	1	0	0	0	horsesho
1017	unnamed	0	100778	26662	###	0	0	0	1	0	1	0	square
94	Kouk Kou	0	200141	1923	80.4	1							horsesho
255	Trapéang Réang	0	100763	6020	###	0	0	0	0	1	0	0	square
1082	Poeng Preah Pram P	0	55303	28072	81.0	0							square
1487	Svay Romeat	0	200132	10828	86.9	0							horsesho
299	Trapeang Chambâk (	0	54800	6903	87.3	1							square
824	Prei Krous I (Pr.)	0	200637	20341	90.0	0	1	0	1	1	0	0	square
254	Kouk Kroes (Pr.)	0	100605	5991	89.8	0	1	1	1	1	0	0	square
470	Kouk Ta Ôk	0	100761	8929	87.6	0	1	0	1	1	0	0	horsesho
902	Tep Pranam (Pr.)	60	47900	23917	88.3	0							
763	#N/A	0	200891	15979	88.5	1	1	1	1	1	0	0	horsesho
987	sculptures	0	47114	26627	90.0	0							square
360	Kouk Kok Cha	0	200385	7854	89.4	0	1	0	0	0	0	0	horsesho
97	Kouk Trapeang Lbau	0	100071	2025	67.0	1							horsesho
56	Kouk Neak Ta Trape	0	100080	1085	###	1	1	0	1	0	0	0	horsesho
677	Toul Kon Tbal	0	200693	12547	76.1	0							square
866	Kouk Trapeang Rom	0	100777	23059	90.0	0	1	0	0	0	0	0	square
836	Tuol Kou	0	100478	21321	88.7	1	1	0	0	0	0	0	horsesho
41	Kouk Pongro	0	200052	846	93.2	1	1	0	1	0	0	0	horsesho
491	Trapéang Khna	0	100719	9069	86.8	1	0	0	1	0	0	0	square
1226	Arak Bak Kâ	0	22416	15046	49.6	0							horsesho
50	Kouk Prey Koy	0	200055	1051	87.5	1							horsesho

Temple ID	name_english	Lustig ID	Archsite ID	Pelle ID	Azimuth	Principle Reservoir	Sandstone	Pink Sandstone	Laterite	Brick	Thmaphnom	other	Morphology
1033	Tuol Prasat Kânchân	0	100668	26682	###	0	1	0	1	1	0	0	square
415	Kouk Yéay Laï	0	100556	8627	80.7	0	0	0	1	0	0	0	horsesho
853	Prei (Pr.)	0	54408	22273	92.2	0	1	0	1	1	0	0	square
656	Trapéang Tasat	0	100594	11520	88.8	1							horsesho
927	temple east of Khlea	0	48006	24008	90.3	0	1	0	1	0	0	0	square
88	Kouk Bos Veang	0	100038	1841	95.7	1							square
707	Trapéang Svay	0	100385	13414	97.8	0	1	0	0	0	0	0	horsesho
1003	Komnâp (Pr.)	657	53003	26644	89.9	0	0	0	1	0	0	0	square
983	remains of a temple	0	47161	26623	89.6	1							square
1011	Neak Ta Dek	0	47157	26654	89.3	0	1	0	1	0	0	0	square
1525	sculptures	0	47151	26657	90.0	0							square
384	Tuol Don Ey	0	200175	8369	87.2	1							horsesho
231	To Be Determined	0	200143	5446	###	1							horsesho
771	O Prasat	0	200729	16428	88.4	1							square
843	Kouk Prasat	0	100664	21857	###	1	1	0	0	1	0	0	horsesho
53	Kouk Kralanh	0	100052	1073	90.0	1							horsesho
134	Tuol Prolay Yean De	0	200103	3150	90.0	0							horsesho
935	Kouk Trapeang Ta Ku	0	100783	24509	90.0	0	1	0	1	0	0	0	square
583	Kouk Ta Srei	0	200471	10159	90.0	1	1	0	0	0	0	0	horsesho
710	Kap Chen (Pr.)	0	21504	13539	90.0	0							horsesho
1055	Kouk Trapeang Kâm	0	100632	26710	90.0	0	0	0	1	1	0	0	square
942	Terrace Q	0	47121	25109	90.3	0	1	0	1	0	0	0	square
306	Trapeang Trach	0	59601	6938	90.0	0	0	0	0	1	0	0	square
98	Kouk Khang Lech	0	100070	2030	83.8	1	1	0	1	0	0	0	horsesho
1072	Kouk Krahung	0	58803	27477	97.5	0	1	0	0	0	0	0	square
427	Kouk Boeng Chhouk	0	200506	8710	90.0	1	1	0	1	1	0	0	horsesho
1035	Kouk Mokak	0	100672	26684	90.0	0	1	0	0	1	0	0	square
709	Phnom Hâp (Pr.)	0	55209	13432	89.0	0	1	0	0	1	0	0	square
841	Kouk Kânchân Snor	0	100625	21758	84.2	1	1	0	0	1	0	0	horsesho
239	Kouk Kou	0	200479	5577	###	1	1	1	1	1	0	0	horsesho
636	Toum Taa	0	200675	11179	90.0	0	1	0	1	1	0	0	square
1029	unnamed	0	100661	26677	###	0	0	0	1	1	0	0	square

Temple ID	name_english	Lustig ID	Archsite ID	Pelle ID	Azimuth	Principle Reservoir	Sandstone	Pink Sandstone	Laterite	Brick	Thmaphnom	other	Morphology
736	Krüs Préah Puth	0	100889	14671	94.0	1							square
948	Terrace O	0	200578	25403	5.6	0							square
725	Trapeang Kânchan T	0	100944	14391	90.7	1	0	0	0	1	0	0	horsesho
1436	Borne	0	100930	4788	75.7	0							square
722	Nokor Krov I	0	200643	14242	89.5	0	1	0	1	0	0	0	horsesho
556	Trapéang Tim	0	100860	9853	96.2	1	1	0	1	1	0	0	horsesho
623	Tuol Ta Méak	0	100419	10760	90.9	1	1	0	0	0	0	0	horsesho
837	Kouk Mân	0	100479	21345	88.8	1	1	0	0	1	0	0	horsesho
631	unnamed	0	100843	11143	90.0	0	1	0	0	0	0	0	horsesho
313	Kouk Trapeang Svay	0	100107	6965	78.3	1	1	0	1	1	0	0	horsesho
833	Kouk Krüs	0	100585	21123	87.1	0	1	0	1	1	0	0	horsesho
910	Preah Pithu, V	0	48400	23968	87.5	0	1	0	0	0	0	0	blob
1025	Mdom Véang	0	100646	26671	90.0	0	0	0	1	1	0	0	square
820	Prei Phnom	0	100834	19839	90.4	0	0	0	1	1	0	0	square
613	Neak Ta Kouk Kânhc	0	100734	10495	95.2	0	1	0	1	0	0	0	horsesho
276	undetermined	0	100204	6544	85.7	1							horsesho
1036	To Be Determined	0	200699	26685	99.6	0	0	0	0	1	0	0	square
420	Kouk Popel Khe	0	200061	8660	79.5	1	1	0	0	1	0	0	horsesho
364	Kouk Ta Mau	0	200300	7949	89.5	1	1	0	1	0	0	0	horsesho
761	Phnom Dei II	618	61301	15929	90.0	0	1	0	0	1	0	0	square
241	#N/A	0	100016	5687	90.0	1							horsesho
541	Veal Rondas	0	100997	9667	87.1	0	1	0	0	0	0	0	square
46	#N/A	0	200489	922	90.0	0	1	0	0	0	0	0	horsesho
1661	Baksei Chamkrong (I	501	49500	23546	89.7	0	0	0	1	1	0	0	
577	Kouk Neak Ta	0	200435	10132	92.6	0	1	0	1	0	0	0	horsesho
220	Kouk Ta Nak	0	200146	5301	93.7	1	1	0	0	0	0	0	horsesho
554	Kouk Lvei	0	100825	9817	90.0	0	1	0	1	0	0	0	horsesho
996	Terrace L	0	47108	26636	90.0	0	1	0	1	0	0	0	square
703	To Be Determined	0	200552	13280	68.1	1	1	0	0	1	0	0	horsesho
120	Kouk Kâmnâp	0	100145	3005	90.0	0	0	0	1	1	0	0	horsesho
580	#N/A	0	200465	10147	90.3	1	1	1	1	1	1	0	horsesho
1450	DELETE	0	200202	7107	88.9	0							square

Temple ID	name_english	Lustig ID	Archsite ID	Pelle ID	Azimuth	Principle Reservoir	Sandstone	Pink Sandstone	Laterite	Brick	Thmaphnom	other	Morphology
1010	To Be Determined	0	100769	26652	82.2	0	1	0	1	1	0	0	square
437	Kouk Kâm Hèng	0	100587	8756	###	1	1	0	0	1	0	0	horsesho
600	#N/A	0	200662	10233	90.4	1	1	0	0	0	0	0	square
309	Kouk Kou	0	100035	6952	87.8	1							horsesho
504	Kouk Trapeang Chhc	0	100691	9179	87.3	1							horsesho
540	Kouk Khlôk	0	100752	9635	94.8	1	1	0	1	0	0	0	horsesho
844	Kouk Neak Ta Kuong	0	100462	21939	85.2	1	1	0	1	1	0	0	2causewa
655	Kouk Ta Kuoy	0	100589	11513	90.0	1	1	0	1	1	0	0	horsesho
860	Kouk Trapeang Run	0	100776	22725	90.2	1	1	0	1	0	0	0	square
1043	To Be Determined	0	200702	26694	66.6	0	0	0	1	1	0	0	horsesho
215	Kong Srok (Pr.)	0	100077	5143	74.1	1	1	0	1	0	0	0	horsesho
610	#N/A	0	200665	10321	###	1	1	0	1	0	1	0	horsesho
756	Put Chhum Rieng (P	0	200732	15448	82.7	1							square
793	cruciform terrace	0	200573	17845	###	0	1	0	0	0	0	0	blob
513	Prei Roka	0	100413	9321	85.1	1	1	0	0	0	0	0	square
490	Tuol Pô Pi	0	100716	9064	###	0	1	0	0	1	0	0	square
184	To Be Determined	0	200698	4210	90.0	0	0	0	0	1	0	0	square
283	Kouk Yeay Moav	0	100323	6700	89.8	1	1	0	1	0	0	0	horsesho
92	Phum Khcay	552	65201	1899	85.6	1	0	0	0	0	0	0	horsesho
639	Kbal Chen (Pr.)	0	56200	11378	90.0	0	1	0	1	1	0	0	square
684	Toul Trapeang Tbal	0	200683	13011	76.4	1							horsesho
344	Kouk Trapeang Khna	0	200303	7509	89.0	1	1	0	1	1	0	0	horsesho
932	To Be Determined	0	200639	24028	89.4	0	1	0	1	0	0	0	square
204	Tuol Roka	0	100100	4632	75.3	1	1	0	0	0	0	0	horsesho
939	Terrace S	795	47119	25014	84.8	0							square
905	Terrace N°4	0	47405	23920	89.1	0	1	0	1	0	0	0	blob
433	Kouk Trapeang Kou	0	100519	8733	89.6	1	1	0	1	1	0	0	horsesho
595	Kouk Prasat Pong To	0	200565	10204	###	1	1	0	0	0	0	0	2causewa
718	Toul Prasat Trapeang	0	51100	13878	85.7	1	1	0	0	1	0	0	horsesho
442	Neak Ta Veang	0	50002	8798	79.4	0	1	0	1	0	0	0	square
850	Tuol Ta Lo	0	100708	22102	81.1	0	1	0	1	1	0	0	horsesho
1079	Trapeang Snao (Pr.)	0	21805	28067	90.0	1							square

Temple ID	name_english	Lustig ID	Archsite ID	Pelle ID	Azimuth	Principle Reservoir	Sandstone	Pink Sandstone	Laterite	Brick	Thmaphnom	other	Morphology
109	Kouk Kou	0	100985	2599	83.5	1	1	0	1	0	0	0	horsesho
695	To Be Determined	0	200697	13200	66.5	0	0	0	0	0	1	0	horsesho
519	Trapeang Kouk Kamı	0	100438	9347	###	1	1	0	0	1	0	0	horsesho
240	Kouk Neakta	0	200147	5610	92.3	1	1	0	0	0	0	0	square
431	Andaung Prasat	0	100590	8722	79.5	1	1	0	1	1	0	0	horsesho
400	Tuol Kouk Srah	0	100530	8512	91.7	1	1	0	0	0	0	0	horsesho
486	Kouk Mon	0	100614	9043	###	1	1	0	0	1	0	0	horsesho
573	Semoan (Pr.)	0	200428	10120	89.3	1	1	0	0	0	0	0	horsesho
322	Kouk Ta Suos	0	200301	7082	90.0	1	1	0	1	0	0	0	horsesho
227	Kouk Ta Sa	0	200651	5412	90.0	1							horsesho
223	Tuol Kânchân Réang	0	100095	5319	75.2	1							2causewa
506	Tuol Svay Sâr	0	100387	9185	90.0	0	1	0	1	1	0	0	horsesho
100	Kouk Kou	0	100068	2048	87.8	1							horsesho
662	Run (Pr.)	0	55000	11940	80.4	0	1	0	1	1	0	0	square
1030	Kouk Prei O	0	100660	26678	88.8	1	1	0	1	1	0	0	square
445	Kouk Daun Kom	0	50700	8819	74.2	1	1	0	0	0	0	0	horsesho
508	Kouk Neak Ta	0	100206	9292	91.3	1	1	0	0	0	0	0	horsesho
766	DELETE	0	200014	16117	90.2	0							horsesho
419	Bos Sok	0	200154	8651	81.5	0							horsesho
440	Kouk Krous Trapean	0	200021	8783	87.6	1	1	0	1	0	0	0	square
6678	To Be Determined		200680	11806	85.0	0							horsesho
118	Kouk Kdei	0	61600	2969	89.9	1	1	1	1	0	0	0	horsesho
611	Kouk Trapeang Koh	0	200667	10329	72.3	1							horsesho
1005	Kouk Trapeang Snôr	0	100862	26646	88.7	1	0	0	0	1	0	0	square
1456	Tuol Snuol	0	100764	8592		0							blob
397	Kouk Neak Ta	0	200003	8479	88.3	1	1	0	1	0	0	0	2causewa
390	Kouk Khvean	0	200480	8450	83.4	1	1	0	1	0	0	0	horsesho
507	Trapéang Chhouk	0	100202	9287	84.6	0							horsesho
1071	Prei Prasat (Pr.)	496	58802	27432	90.3	0	0	0	1	1	0	0	square
697	Prei (Pr.)	650	52500	13227	74.2	0	1	0	1	0	0	0	square
576	Kouk Chheu Khmao	0	200434	10129	###	1	1	0	0	1	0	0	square
872	Ta Prohm Kel	721	49800	23230	90.0	0	1	0	1	0	0	0	square

Temple ID	name_english	Lustig ID	Archsite ID	Pelle ID	Azimuth	Principle Reservoir	Sandstone	Pink Sandstone	Laterite	Brick	Thmaphnom	other	Morphology
199	Kouk Trapeang Amp	0	100099	4529	66.7	1	1	0	0	0	0	0	horsesho
776	Trapeang Beng (Pr.)	0	21204	16691	88.7	1							horsesho
361	Kouk Ta Kong	0	200305	7874	90.6	0	1	0	1	0	0	0	horsesho
906	Preah Pithu, U	0	48200	23963	85.8	0	1	0	0	0	0	0	square
469	Kouk Krüs	0	100371	8924	88.8	0	1	0	0	1	0	0	horsesho
55	Kouk Trapeang Krap	0	100062	1081	85.3	1							horsesho
125	To Be Determined	0	200124	3043	90.0	0							horsesho
122	To Be Determined	0	100148	3019	91.5	1	1	0	1	1	0	0	horsesho
121	DELETE	0	200123	3013	90.3	0							horsesho
6432	DELETE		100144	2998	90.0	0							horsesho
116	Wat Slat	0	100118	2936	90.0	1							2causewa
236	Kouk Ondong Ta Ton	0	200148	5530	###	1							horsesho
657	Kouk Kâmnâp	0	100615	11543	90.0	0	1	0	1	1	0	0	square
1075	Kouk Ponlei	0	200730	28054	90.0	1							square
250	To Be Determined	0	200670	5900	88.2	0	1	0	1	0	0	0	square
434	Kouk Daun Téav	0	100554	8737	89.5	1	1	0	1	0	0	0	horsesho
7	Kouk Khvean	0	200004	48	86.0	1	1	0	1	0	0	0	horsesho
104	Kouk Kou	0	200129	2426	67.7	1	1	0	1	0	0	0	horsesho
1042	To Be Determined	0	200703	26692	95.6	0	0	0	0	1	0	0	square
280	Kouk Kroes	0	200203	6687	90.8	0	0	0	1	1	0	0	horsesho
592	Tuol Prasat Srah Kou	0	200538	10191	83.1	1	0	0	1	0	0	0	horsesho
1057	Poy Ta Chap (Pr.)	0	100634	26712	90.0	0	1	0	1	1	0	0	square
586	Kouk Kou Trapeang I	0	200482	10168	90.5	1	1	0	1	1	0	0	horsesho
380	Kouk Samong	0	200502	8351	###	0	0	0	0	1	0	0	horsesho
51	Kouk Kou	0	200056	1056	89.4	0							horsesho
730	Neak Ta Bak Ka	0	22300	14483	86.6	1							
528	Prei Neak Ta Dek	0	100742	9540	88.6	0	1	0	0	1	0	0	horsesho
58	Kouk Rom Dol	0	200461	1105	90.5	0							square
838	Kouk Ta Yâ	0	100577	21359	89.9	1	0	0	0	1	0	0	horsesho
782	Roluos (Pr.)	0	100124	17185	90.0	0	0	0	0	1	0	0	square
566	Kouk Ta Chat	0	100065	10091	70.4	1							horsesho
512	Kouk Kroes	0	100412	9317	92.7	1	1	0	1	1	0	0	horsesho

Temple ID	name_english	Lustig ID	Archsite ID	Pelle ID	Azimuth	Principle Reservoir	Sandstone	Pink Sandstone	Laterite	Brick	Thmaphnom	other	Morphology
970	Sanctuary N	0	47131	26610	88.0	0	0	0	1	0	0	0	square
713	Trapeang Sangkae (F	0	56408	13630	84.0	1							square
1027	Kouk Trapeang Ien	0	100623	26675	88.6	0	1	0	0	0	0	0	square
93	Bos Kou	0	200142	1908	89.1	0							horsesho
812	Daun Yât	0	100829	19242	91.4	0							horsesho
1659	Phimeanakas (Pr.)	61	47602	23897	89.6	0	1	0	1	0	0	0	
558	Kânchân Svay	0	100891	9869	90.0	1							horsesho
147	Kouk Ta Mau	0	200008	3399	78.5	1	0	0	1	1	0	0	horsesho
64	Kouk Trapeang Cho	0	200067	1224	69.4	1	1	1	1	1	0	0	horsesho
813	To Be Determined	0	200636	19246	###	0							horsesho
930	Terrace N°2	0	47188	24013	88.7	0	1	0	1	0	0	0	square
553	unnamed	0	100774	9812	90.0	0	1	0	0	0	0	0	horsesho
888	Preah Kouk Thlôk	0	47403	23863	85.3	0							square
561	Kouk Châk (Pr.)	0	58906	10044	92.3	1	1	0	0	0	0	0	square
618	Phum Prasat (Pr.)	0	54604	10677	90.0	1	1	0	1	1	0	0	horsesho
1013	Kouk Dong 3	0	200632	26658	90.0	0	0	0	1	1	0	0	square
957	sculptures	0	47146	26594	89.3	0							square
13	Kouk Kou	0	63700	237	96.6	1	0	0	1	0	0	0	horsesho
851	Noreay (Pr.)	0	51603	22122	###	1	1	0	0	0	0	0	horsesho
391	Kouk Krabei Riel	0	50500	8457	90.0	1	1	0	0	1	0	0	horsesho
226	Kos Kou	0	200463	5385	90.0	1							horsesho
811	Trapéang Arak Svay	0	100400	19225	80.1	1	1	0	0	1	0	0	horsesho
405	Srok Russei (Pr.)	0	100515	8553	79.5	1	1	0	1	1	0	0	horsesho
346	Tuol Ta Sok	0	200445	7597	###	0	1	0	1	0	0	0	horsesho
432	Kouk Prasat	0	100593	8730	92.9	1	1	0	1	1	0	0	horsesho
237	Kouk Bos Kralanh	0	100021	5550	97.2	0							horsesho
14	Kouk Trapeang Lbau	0	200006	254	84.1	1	1	0	1	1	0	0	horsesho
292	Kanh Chon Chuk	0	200036	6872	81.0	1							square
193	Kouk Ta Srei	0	60400	4345	85.5	1	1	0	1	0	0	0	horsesho
641	Wat Kouk Khpuos	0	59103	11385	90.0	1	0	1	0	0	0	0	horsesho
816	Kânchân Trâméang I	0	100689	19368	90.1	0	0	0	1	1	0	0	horsesho
840	Kouk Wat (Pr.)	0	100622	21649	###	0	1	0	0	1	0	0	horsesho

Temple ID	name_english	Lustig ID	Archsite ID	Pelle ID	Azimuth	Principle Reservoir	Sandstone	Pink Sandstone	Laterite	Brick	Thmaphnom	other	Morphology
108	Kouk Yeay Love	0	100110	2552	80.0	1							horsesho
252	Daun Chan (Pr.)	0	21500	5959	90.5	0							horsesho
77	Kouk Kou Prey Antol	0	100081	1501	94.8	1	1	0	1	0	0	0	horsesho
492	Ta Chikrey (Pr.)	0	100720	9070	94.5	0	1	0	1	0	0	0	square
383	Kouk Don Mei	0	200138	8367	70.1	1							horsesho
1445	Pakaong	0	100534	6085	91.0	0	0	0	0	1	0	0	square
349	Khbon (Pr.)	0	57301	7641	90.2	0	1	0	1	1	0	0	horsesho
72	DELETE	0	200068	1420	65.7	1							square
62	Kouk Kou	0	200064	1153	73.9	0	1	1	1	1	0	0	horsesho
183	Kouk Thkov	0	100876	4192	88.4	1	1	0	0	1	0	0	horsesho
708	Trapéang Bœng Phlc	0	100407	13419	90.0	0	0	0	1	1	0	0	horsesho
598	#N/A	0	200660	10221	90.0	1	1	0	1	0	0	0	horsesho
1083	Don Meas (Pr.)	0	55611	28073	90.0	0	0	0	0	1	0	0	square
825	Prei Kreuk (Pr.)	0	200638	20538	89.2	0	1	0	1	1	0	0	4causewa
6554	DELETE		100314	11273	90.0	1							square
665	Ta Va (Pr.)	0	100173	11985	89.1	0	1	0	1	0	0	0	square
91	Kouk Romchek	0	100032	1891	90.0	1	1	0	0	1	0	0	horsesho
343	Kouk Som Rong	0	200226	7486	###	1	1	0	0	1	0	0	horsesho
719	To Be Determined	0	200684	13919	84.6	0	1	0	1	0	0	0	horsesho
565	#N/A	0	100041	10086	###	1	0	0	1	1	1	0	horsesho
295	Kouk Prei Sa-oy	0	51200	6887	84.3	1	1	0	0	1	0	0	horsesho
18	Kouk Bos Ta Chet	0	200048	368	93.7	1	1	1	0	1	0	0	horsesho
31	Sonday	0	100162	670	89.8	1	0	0	0	1	0	0	square
208	Kouk Trapeang Ron	0	200167	4829	67.4	1	1	0	0	0	0	0	2causewa
270	Kouk Ta Krem	0	100224	6455	90.0	0							horsesho
1054	Kouk Trapeang Kâm	0	100633	26709	92.9	0	0	0	1	1	0	0	square
1091	DELETE	0	200214	7017	92.0	0							
312	Kouk Kou Trapeang	0	100047	6963	94.8	1	1	0	1	1	0	0	horsesho
517	Kânchân Ta Sék	0	100436	9339	91.1	1	1	0	0	0	0	0	square
597	#N/A	0	200659	10210	90.0	1	1	0	0	0	0	0	horsesho
269	Kouk Thmei	0	100225	6450	89.5	0							square
318	Kouk Kanhchoan Ta	0	200213	7037	90.0	1	0	0	1	1	0	0	square

Temple ID	name_english	Lustig ID	Archsite ID	Pelle ID	Azimuth	Principle Reservoir	Sandstone	Pink Sandstone	Laterite	Brick	Thmaphnom	other	Morphology
293	Kouk Kos	0	100578	6875	68.5	0	1	0	0	0	0	0	square
404	Kouk Kâmnâp	0	100684	8550	93.9	0	0	0	1	0	0	0	horsesho
676	Banteay Kdei (Pr.)	577	53500	15845	86.6	1							4causewa
500	Tuol Pao	0	100550	9127	90.0	1							horsesho
234	Kouk Neakta	0	200149	5507	90.0	1	1	1	1	1	0	0	horsesho
279	Kro Lok Konseng	0	100208	6651	94.6	1							horsesho
244	Kouk Soeng	0	100915	5743	84.6	1							horsesho
774	#N/A	0	200761	16585	90.8	0	0	0	1	1	0	0	horsesho
32	Kouk Trapeang Kouk	0	200026	680	58.9	1							square
85	Kouk Trapeang Ka N	0	100043	1736	90.0	1	0	0	1	0	0	0	2causewa
87	Kouk Bos Veang	0	100037	1827	99.7	1							horsesho
381	Kanhchon Hom	0	200116	8362	###	1	1	0	0	1	0	0	horsesho
599	#N/A	0	200661	10229	91.7	0	1	0	0	1	0	0	horsesho
661	Trapeang Thlok Bau	0	100428	11888	89.7	1	0	1	0	0	0	0	horsesho
42	Kouk Kou	0	200045	862	###	0	1	0	1	0	0	0	horsesho
664	To Be Determined	0	200597	11944	78.8	1	0	0	1	1	1	0	horsesho
354	Tuol Bos Veang	0	200210	7713	94.2	1	1	0	0	0	0	0	horsesho
49	Kouk Kou Dok Por	0	100046	981	90.0	1	1	0	1	0	0	0	horsesho
149	Srei Ronas (Pr.)	0	100186	3429	90.0	1	1	0	1	0	1	0	horsesho
333	Kouk Kroes	0	200201	7256	80.4	1	1	0	1	1	0	0	horsesho
1009	unnamed	0	100768	26650	86.9	0	1	0	1	1	0	0	horsesho
330	Daun Diu (Pr.)	0	56700	7180	89.6	1	1	0	1	1	0	0	horsesho
686	Kouk Daung	0	60200	13015	88.0	0	0	0	0	1	0	1	horsesho
52	Kouk Om	0	100057	1072	90.0	0							2causewa
1020	Trapéang Ta Loich	0	100652	26665	83.9	0	1	0	0	0	0	0	square
1023	Kouk Rüssei	0	100658	26668	90.8	1	1	0	0	0	0	0	square
30	Kouk Kou	0	200031	627	###	0							horsesho
604	Kouk Trapeang Snou	0	200209	10296	91.5	1	1	0	1	0	0	0	horsesho
773	Prei Khmang (Pr.)	0	200606	16496	90.3	1							2causewa
297	Kouk Thlok (Pr.)	0	54407	6895	90.0	1	1	0	0	1	0	0	horsesho
369	Kouk Yeang Daoch	0	100344	8099	###	1	1	0	1	1	0	0	horsesho
1070	Sema Don Meas	0	55612	27360	88.8	0							square

Temple ID	name_english	Lustig ID	Archsite ID	Pelle ID	Azimuth	Principle Reservoir	Sandstone	Pink Sandstone	Laterite	Brick	Thmaphnom	other	Morphology
728	Ta Muong	0	47306	14452	87.1	1	1	0	1	0	0	0	square
285	Krulok Tasek	0	200196	6725	83.6	1							2causewa
574	Kouk Ta Soeng	0	200430	10126	###	1	1	0	0	1	0	0	square
845	unnamed	0	100707	21946	89.6	1							horsesho
304	Daun So	504	58903	6928	89.3	1	1	0	1	0	0	0	horsesho
138	Kouk Thas	0	100991	3241	91.2	1	1	0	0	1	0	0	square
74	Kouk Chambok Sow	0	200058	1487	90.0	1	1	0	1	0	1	0	2causewa
216	Kouk Chom Bok	0	100073	5159	83.9	1							horsesho
17	Kouk Trapeang Plus	0	200043	326	92.1	1	1	0	1	1	1	0	horsesho
1498	DELETE	0	100841	11564	91.8	0							square
47	Trapeang Song Ke (P	0	100049	939	90.0	1	1	1	1	1	0	0	horsesho
222	Tuol Srah Kou	0	100542	5318	75.2	1	0	0	0	1	0	0	2causewa
12	Kouk Bos Krolor	0	200002	225	89.4	1	1	1	1	1	0	0	square
272	Don Pheng (Pr.)	0	200194	6506	90.0	1	0	0	1	1	0	0	horsesho
998	Prasat Chey Emey	0	100894	26639	82.3	0	1	0	1	0	0	0	square
334	Kouk Krous	0	200221	7281	90.5	1	1	0	1	1	0	0	horsesho
480	Tuol Trapéang Svay	0	100566	9004	99.2	0							horsesho
296	Kouk Ta Ro	0	51300	6890	89.6	0	0	0	0	1	0	0	horsesho
6847	Kanhchon Chrov		200009	91	###	1							square
362	Kouk Ta Sen	0	200227	7919	###	1	1	0	0	0	0	0	horsesho
578	Kouk Chas Khang Le	0	200446	10133	96.9	1	1	0	1	0	0	0	horsesho
570	Krous Prasat Lich	0	200419	10113	91.3	0	1	0	1	1	0	0	horsesho
26	Chranieng (Pr.)	0	200033	539	89.2	1	0	0	0	1	0	0	horsesho
602	#N/A	0	100022	10244	91.6	1	1	0	1	0	0	0	horsesho
232	Kouk Bos Kralanh	0	100028	5468	90.0	1	1	0	0	1	0	0	2causewa
426	Kouk Trapeang Chre	0	100604	8700	78.0	1	0	0	1	0	0	0	horsesho
1510	Kanchan Reang	0	100129	14423	90.8	1							square
1434	Kouk Trapeang Pring	0	100174	3788	93.3	1							square
332	Ta Chey (Pr.)	0	100315	7223	87.2	1	1	0	1	0	0	0	horsesho
781	Kouk Rüssei Dop	0	100128	17140	89.4	0							horsesho
214	Kouk Ta Neang	0	100086	5097	91.4	1							horsesho
101	Kouk Bos Som	0	100069	2056	95.8	1	1	0	0	1	0	0	horsesho

Temple ID	name_english	Lustig ID	Archsite ID	Pelle ID	Azimuth	Principle Reservoir	Sandstone	Pink Sandstone	Laterite	Brick	Thmaphnom	other	Morphology
1006	Kouk Ta Kong	0	53801	26647	89.0	0	1	0	1	1	0	0	square
308	Kouk Trapeang Krol	0	62500	6947	80.3	1	1	0	1	0	0	0	horsesho
323	Kouk Prasat Khnar T	0	100325	7101	89.6	1	1	0	1	1	0	0	horsesho
103	Ka-aek Tum	0	200137	2410	93.2	1	1	0	0	1	0	0	2causew
356	Kouk Neak Ta Ong	0	200229	7734	90.0	1	0	0	1	0	0	0	horsesho
221	To Be Determined	0	200152	5316	90.0	1	1	0	1	0	0	0	horsesho
268	Trapeang Ampil	0	200197	6439	90.6	1	1	0	1	0	0	0	square
1049	Kouk Ta Kè	0	100674	26702	93.3	0	0	0	1	1	0	0	square
166	Kouk Kroes	0	61003	4044	90.7	1							horsesho
1516	#N/A	0	200897	20243	###	1							blob
652	Trapeang Khna Thm	0	100495	11487	91.9	1	1	0	0	1	0	0	horsesho
770	Ampil Thvear Prei (P	0	200728	16410	89.5	1							horsesho
277	Kanhchon Ta Em	0	100203	6596	95.8	1							horsesho
340	Kouk Kroes	0	200690	7436	88.8	0							horsesho
7049	To Be Determined		200653	8373	89.1	0							horsesho
829	Trapéang Kom Phnié	0	100621	20852	67.0	1							square
229	Kouk Bos Thom	0	100030	5434	###	1	1	0	1	1	0	0	horsesho
132	Prei Vihéar	0	100988	3130	84.2	0	1	0	1	1	0	0	horsesho
750	Kôk Balang	0	22417	15194	68.7	0							
235	unnamed	0	200150	5511	90.4	1	1	0	0	0	0	0	horsesho
529	Tuol Ta Préap	0	100527	9543	84.4	0	1	0	1	0	0	0	horsesho
127	Bos Thmâr	0	100143	3058	90.0	1	0	0	0	1	0	0	horsesho
955	Preah Si Ar	0	47401	26308	83.2	0							square
532	Kouk Srah	0	100538	9566	95.8	1	1	0	0	0	0	0	horsesho
499	Trapeang Thlok (Pr.)	0	100549	9123	89.6	1							horsesho
218	Kouk Kou	0	100082	5277	93.8	1							horsesho
69	Kroluk Thkov	0	100151	1376	86.0	0							horsesho
7021	DELETE		200598	9151		0							blob
96	Kouk Khdoch	0	100044	1957	91.7	0							horsesho
1037	Kouk Ta Ang	0	100676	26686	77.5	0	0	0	0	1	0	0	square
870	Trapeang Kbal Damr	0	49702	23142	90.2	0	1	0	0	1	0	0	horsesho
417	Trapeang Klong (Pr.)	0	100427	8640	90.0	1	1	0	0	1	0	0	horsesho

Temple ID	name_english	Lustig ID	Archsite ID	Pelle ID	Azimuth	Principle Reservoir	Sandstone	Pink Sandstone	Laterite	Brick	Thmaphnom	other	Morphology
233	Kouk Ta Som	0	200145	5474	90.0	1	1	0	1	1	0	0	horsesho
642	Wat Kandaol Pô Pro	0	61700	11399	90.0	0	1	0	1	0	0	0	horsesho
1008	unnamed	0	100831	26649	85.6	1	1	0	1	0	0	0	square
1031	Wat Prasat	0	59100	26679	98.8	0	1	0	0	1	0	0	square
6005	Krol Kô (Pr.)		52600	17225	88.0	0	1	0	1	0	0	0	horsehoe
245	Trapeang Chuk	0	100322	5749	###	1	1	0	1	1	0	0	square
1499	DELETE	0	100856	11565		0							square
762	#N/A	0	200892	15973	77.7	1							square
20	Kouk Trapeang Yey C	0	200049	397	90.4	1	0	0	0	1	0	0	horsesho
22	Kouk Kou	0	200038	450	90.0	0	1	0	1	1	0	0	horsesho
515	Kouk Ta Meun	0	100421	9334	90.9	0	1	0	1	1	0	0	horsesho
909	Preah Pithu, X	0	48300	23966	90.0	0	1	0	1	0	0	0	blob
19	Prey Kou	0	200050	376	85.8	1	1	1	0	1	0	0	horsesho
186	Kouk Kandal	0	62000	4268	90.0	1	0	0	0	1	0	0	horsesho
291	Kouk Ta Chhâkk	0	100477	6870	78.2	1	1	0	1	0	0	0	horsesho
319	Kouk Chombok Lor	0	100326	7059	90.0	1	1	0	1	1	0	0	2causewa
551	Kouk Roka	0	100368	9771	90.4	0	1	0	1	1	0	0	horsesho
126	Kouk Pongro (Pr.)	0	100146	3054	94.3	1	0	0	0	1	0	0	horsesho
520	Trapéang Pring	0	100442	9355	96.9	1	0	0	1	0	0	0	horsesho
267	DELETE	0	200507	6428	###	0							horsesho
768	To Be Determined	0	200016	16222	87.2	0	1	0	0	1	0	0	horsesho
669	Tor (Pr.)	585	54300	12101	87.7	0	0	0	1	0	0	0	horsesho
647	Réan Andœk Touch (	0	100443	11447	89.6	1	1	0	1	1	0	0	horsesho
76	Kouk Beng	0	200057	1497	86.3	1	1	0	0	1	0	0	horsesho
6685	Tuol Krâpeu Slap		100755	6160	79.5	0	1	0	0	0	0	0	square
734	Krüs Daun Moal	0	100490	14612	###	0	0	0	0	1	0	0	horsesho
797	Ta Mao (Pr.)	0	47154	18024	###	0	1	0	1	0	0	0	2causewa
401	Kouk O Chrung	503	54600	8524	90.8	1	0	0	0	1	0	0	horsesho
392	Kouk Bankrai	0	51500	8460	88.1	0	1	0	0	0	0	0	horsesho
212	Kouk Kou	0	100075	5032	91.3	1							horsesho
1478	#N/A	0	200490	10171	###	0	0	0	1	0	0	0	square
408	Kouk Yéay Ong	0	100402	8571	85.8	1	1	0	1	1	0	0	horsesho

Temple ID	name_english	Lustig ID	Archsite ID	Pelle ID	Azimuth	Principle Reservoir	Sandstone	Pink Sandstone	Laterite	Brick	Thmaphnom	other	Morphology
310	Kouk Ta Di	0	100036	6955	85.7	0							horsesho
410	Kroes Kouk Kanhcha	0	100381	8588	90.0	1	0	0	1	1	0	0	horsesho
57	Kouk Khtom	0	100058	1098	89.8	1							horsesho
849	Trapéang Thlok	0	100458	22083	96.7	0							horsesho
4	O Yeang (Pr.)	0	63300	33	###	1	1	0	0	1	0	0	horsesho
435	Kouk Arak (Pr.)	0	100584	8746	90.6	1	1	0	1	1	0	0	horsesho
144	Kouk Krous	0	100161	3343	89.4	1	1	0	1	1	0	0	horsesho
1095	DELETE	0	100879	11161	85.0	0							square
729	To Be Determined	0	200628	14454	81.6	0							horsesho
555	Trapéang Thlok Dau	0	100859	9850	95.5	1							horsesho
164	Tuol Trapeang Svay	0	100140	3978	91.7	0							horsesho
205	Kouk Chan	0	62300	4688	73.9	1	1	0	0	1	0	0	horsesho
559	Kouk Neakta	0	200205	9944	91.3	1	1	0	0	0	0	0	horsesho
447	Kouk Sla Ket (Pr.)	538	51400	8829	90.0	1	0	0	0	1	0	0	horsesho
217	Kouk Ta Meakh	0	100074	5175	80.2	1	1	0	1	1	0	0	horsesho
716	Ta Moni (Pr.)	0	60204	13779	89.4	0	1	0	0	1	0	0	horsesho
501	To Be Determined	0	200442	9157	87.9	1	1	0	1	1	0	0	2causewa
61	Wat Thipadei	520	63600	1145	91.9	1	1	0	0	1	0	0	horsesho
1522	Tuol Trapéang Anch	0	100677	21806		0							blob
256	Reou (Pr.)	0	21202	6130	85.1	1							horsesho
290	Trav Treang (Pr.)	0	51000	6859	85.1	1	1	0	0	1	0	0	horsesho
338	Phnom Phu (Pr.)	0	57100	7388	90.0	1	0	0	0	1	0	0	horsesho
95	Wat Khcheay	0	100034	1945	90.0	0	1	0	0	0	0	0	2causewa
48	Kouk Kou Trapeang	0	100050	943	88.5	1	1	0	0	0	0	0	horsesho
368	Tuol Kouk Dombek	0	200304	8086	90.0	0	1	0	1	0	0	0	horsesho
1053	Tuol Prasat	0	59203	26708	90.0	0	1	0	1	1	0	0	square
608	Tuol Trapeang Yey P	0	200224	10308	82.8	0	1	0	1	0	0	0	square
374	Kouk Phkam	0	100341	8257	###	1	1	0	1	0	0	0	horsesho
871	Bay Ka-ek (Pr.)	0	49900	23224	86.6	0	1	0	1	0	0	0	horsesho
421	Kouk Trapeang Kou	0	200458	8661	90.4	1							square
242	#N/A	0	100015	5700	###	1	1	0	1	1	0	0	horsesho
524	Kroes (Pr.)	0	100494	9394	90.0	0	1	0	1	1	0	0	horsesho

Temple ID	name_english	Lustig ID	Archsite ID	Pelle ID	Azimuth	Principle Reservoir	Sandstone	Pink Sandstone	Laterite	Brick	Thmaphnom	other	Morphology
848	Tuol Kânchân Kou	0	100586	22028	85.4	1							horsesho
78	Kouk Trapeang Prea	0	200078	1510	90.0	1	1	0	0	0	0	0	horsesho
177	Tuol O Prasat Sralao	0	100130	4174	90.6	1	1	0	0	1	0	0	square
148	Tuol Prasat Prei Pol	0	200187	3419	89.1	0	0	0	1	0	0	0	horsesho
696	Trapeang Prei (Pr.)	0	100171	13223	###	1	0	0	1	1	0	0	square
514	Tuol Romduol	0	100417	9330	83.6	0							horsesho
65	Kouk Pongro	0	100116	1254	71.1	0	1	0	0	1	0	0	square
135	Kouk Trapeang Rona	0	100150	3165	90.0	1	1	1	0	1	0	0	horsesho
331	Beng Laâk (Pr.)	0	56502	7199	89.5	1	1	0	0	0	0	0	horsesho
336	Sema (Pr.)	0	56501	7361	90.0	1	1	0	1	1	0	0	square
1034	Kouk Béng	0	100626	26683	###	0	1	0	1	1	0	0	square
213	Kouk Kou	0	100078	5055	74.7	1	1	0	1	1	0	0	square
828	Kouk Bœng Kândal	0	100620	20841	70.0	1	1	0	1	1	0	0	horsesho
516	Tuol Koa	0	100423	9335	80.1	1							horsesho
80	Kouk Bos Preal	0	200041	1564	93.2	0							horsesho
733	#N/A	0	100245	14566	90.0	1							horsesho
1503	Kouk Kâmnâp	0	100903	12248		0							square
1051	Tuol Kânhchié Daun	0	100628	26705	82.6	1	0	0	1	1	0	0	square
581	Kouk Kanhchon Cha	0	200467	10148	91.1	1							square
439	Kouk Prasat	0	100476	8777	88.7	1	0	0	1	0	0	0	horsesho
847	Châmbâk Kroluoch	0	100597	21981	73.8	0	0	0	1	1	0	0	horsesho
749	Chum Teav (Pr.)	0	100185	15160	90.0	1	1	0	1	1	0	0	2causewa
210	Kouk Thmei	0	200156	4930	91.0	0	0	0	1	0	0	0	2causewa
691	Trapeang Prei (Pr.)	0	56407	13047	87.4	1							horsesho
441	Kouk Ta Men	0	100990	8795	86.0	1	1	0	0	1	0	0	square
328	Khla Krahoem (Pr.)	0	56600	7163	###	1	1	0	1	1	0	0	horsesho
683	Kouk Ta Tong	0	200682	13010	79.9	1	0	0	1	1	1	0	horsesho
1024	Bantéay Chheu	0	100460	26669	75.9	0	0	0	1	1	0	0	square
106	Kouk Takel	0	200130	2468	85.3	1	1	1	1	1	0	0	horsesho
869	Prasat Thom	0	100653	23128	88.3	0	1	0	0	1	0	0	horsesho
1486	Kouk Ta Kuong	0	54411	10667	###	0	1	0	1	0	0	0	square
155	Prey Krous	0	100167	3761	90.0	1							horsesho

Temple ID	name_english	Lustig ID	Archsite ID	Pelle ID	Azimuth	Principle Reservoir	Sandstone	Pink Sandstone	Laterite	Brick	Thmaphnom	other	Morphology
238	Kouk Thnaot	0	100017	5572	###	1	1	0	0	1	0	0	horsesho
5730	Chrei (Pr.)		21800	14963	89.8	0							4causewa
666	Kouk Ta Och	0	100489	12022	91.7	1	1	0	1	1	0	0	horsesho
632	Kouk Khlôk	0	100845	11147	85.9	1	0	0	1	0	0	0	square
168	Srei Chumreov (Pr.)	0	100541	4092	87.0	1	1	0	1	1	0	0	horsesho
834	Phum O (Pr.)	0	59102	21203	89.7	1	1	0	1	1	0	0	horsesho
206	Kouk Trapeang Kou	0	100102	4736	83.9	1							horsesho
1052	Kouk Trapeang Snuo	0	100627	26706	78.4	0	1	0	1	1	0	0	square
258	Kouk Koh Kou	424	100424	6141	83.5	1	1	0	1	1	0	0	horsesho
778	Sek Ta Tuy (Pr.)	103	21302	16745	90.0	0							square
852	Tuol Prasat Kô Vien	0	100456	22247	89.4	1							horsesho
294	Wat Pama	0	50800	6883	90.0	1	1	0	1	0	0	0	square
753	Prei Prasat (Pr.)	0	51900	15385	90.0	1	1	0	1	0	0	0	2causewa
355	Kouk Ta Sin	0	100348	7727	89.0	1	1	0	1	1	0	0	horsesho
1044	Kouk Ta Kè	0	100673	26697	88.6	0	1	0	1	1	0	0	square
73	Kouk Ta Hok	0	200404	1458	90.8	1	0	0	1	1	0	0	horsesho
71	Veal Trapeang Ka Ab	0	200081	1416	82.1	1							square
1038	Trapeang Trâbèk (Pr.	0	100629	26687	86.6	1	1	0	1	1	0	0	square
82	Kouk Chan	0	64800	1598	###	1	1	0	1	1	0	0	horsesho
141	Kouk Chrap	0	200010	3283	###	1							horsesho
207	DELETE	0	100954	4803	73.3	1							horsesho
1488	Kouk Dâng Kâmbet	0	100377	10896	90.7	0							blob
90	Kouk Ta Lom	0	100031	1887	91.8	1							horsesho
389	Kouk Kou	0	200473	8447	68.8	0	1	1	0	1	0	0	horsesho
735	Kroes Chieng Khim	0	100887	14624	86.3	1	1	0	1	1	0	0	horsesho
185	Kouk Thbong (Pr.)	0	60800	4231	###	1	1	0	0	1	0	0	horsesho
678	Trapéang Ta Tuot	0	100618	12591	90.0	1	1	0	1	1	0	0	horsesho
367	Kouk Ta Sen	0	200306	8042	78.7	0	0	0	1	0	0	0	horsesho
379	Kouk Kanes	0	200207	8323	94.1	1	1	0	0	0	0	0	horsesho
624	Kouk Kâmnâp	0	100175	10775	88.4	0	0	0	1	1	0	0	square
324	Kouk Kroes	0	200215	7119	88.9	1	1	0	1	1	0	0	horsesho
388	Khrus (Pr.)	0	200392	8445	90.0	0	1	1	1	1	0	0	2causewa

Temple ID	name_english	Lustig ID	Archsite ID	Pelle ID	Azimuth	Principle Reservoir	Sandstone	Pink Sandstone	Laterite	Brick	Thmaphnom	other	Morphology
831	Prei Khmeng (Pr.)	124	59400	20916	68.1	0	0	0	0	1	0	0	HORsesh
1464	Kouk Chong Spean	0	100740	9470		0	1	0	0	0	0	0	blob
1520	Kouk Yéay Hèam	0	100675	21715		0							blob
889	Preah En Tep	0	47404	23864	89.7	0							square
423	Kouk Chas	0	100602	8683	74.4	1	0	0	1	1	0	0	horsesho
668	Truong Chrouk (Pr.)	0	47172	12097	86.1	0	1	0	1	0	0	0	horsesho
521	To Be Determined	0	200664	9363	89.4	0	0	0	1	0	0	0	horsesho
196	Thnâl Ta Ek	0	200536	4401	89.5	1	1	0	0	0	0	0	horsesho
503	Trapeang Chong (Pr.	0	56100	9169	87.3	0	1	0	1	1	0	0	horsesho
534	Trapeang Prom Ly	0	100725	9595	90.0	0	1	0	1	1	0	0	horsesho
370	Kouk Trâbaek	0	200308	8114	###	1	1	0	1	1	0	0	horsesho
429	Kouk Prâvas	0	100473	8715	90.0	0	1	0	1	1	0	0	horsesho
21	Kouk Khdei	0	200042	425	95.5	0							horsesho
63	Preah Enkosei (Pr.)	53	54400	1171	98.4	1	0	0	0	1	0	0	horsesho
395	Tuol Kouk Kroeul	0	200176	8470	84.9	0							square
907	Preah Pithu, T	578	48100	23964	86.8	0	1	0	0	0	0	0	square
15	Kouk Trapeang Vean	0	200051	276	###	0	1	0	0	1	0	0	horsesho
769	Totei (Pr.)	0	200015	16322	89.1	0	1	0	0	1	0	0	horsesho
588	Kouk Tanong	0	200498	10174	93.9	0	0	0	1	0	0	0	horsesho
129	Tuol Tamnup La Hou	0	100149	3077	74.6	0	1	0	0	0	0	0	horsesho
169	Kouk Krous	0	100168	4106	89.2	1	1	0	0	1	0	0	horsesho
653	Kouk Trâbèk	0	100503	11490	91.5	1	1	0	0	0	0	0	horsesho
113	Kanhchon Ta Phal	0	200134	2800	90.0	0							horsesho
133	Kâmboch (Pr.)	0	100989	3141	90.0	1	0	0	0	1	0	0	horsesho
243	Tuol Prasat Kouk Sa	0	200576	5734	78.3	1	1	1	1	1	0	0	horsesho
117	Tamoch (Pr.)	0	61800	2943	90.0	1	1	0	1	1	0	0	horsesho
867	DELETE	0	49603	23090	94.5	0							2causewa
446	Trapeang Tasath (Pr.	0	50701	8824	89.4	1	1	0	1	1	0	0	horsesho
606	Tuol Trapeang Chon	0	200222	10300	###	1	1	0	1	0	0	0	square
494	Wat Ampil	0	100565	9084	78.2	1	1	0	1	1	0	0	square
539	Tuol Kânchân Ta Em	0	100750	9630	79.1	0							horsesho
471	Kouk Dâng Kâmbet	0	100376	8937	90.8	1	0	0	1	1	0	0	horsesho

Temple ID	name_english	Lustig ID	Archsite ID	Pelle ID	Azimuth	Principle Reservoir	Sandstone	Pink Sandstone	Laterite	Brick	Thmaphnom	other	Morphology
303	Tram Neak	0	57803	6920	84.2	1	1	0	1	0	0	0	horsesho
626	Sralao Srong (Pr.)	0	200020	10813	81.3	1	0	0	1	1	0	0	square
587	Kouk Khcheay	0	200497	10173	###	1	1	0	1	0	0	0	square
9	Prei Kouk Ta Ek	0	100913	71	86.7	0	0	0	1	0	0	0	square
1001	Kouk Khmoch	0	100389	26642	89.7	1	0	0	1	0	0	0	square
143	Tuol Daun Chê	0	100160	3336	90.0	1	1	0	1	0	0	0	horsesho
601	#N/A	0	200663	10239	93.5	1	1	0	0	0	0	0	square
1012	To Be Determined	0	200645	26656	87.8	0	1	0	1	0	0	0	square
107	Kouk Trapeang Ta Ve	0	62200	2512	90.0	1							horsesho
342	Kouk Ta Sen	0	200219	7470	###	1	1	1	0	1	0	0	horsesho
200	Kouk Soeng 2	0	100096	4539	82.5	1	1	0	0	1	0	0	horsesho
612	Kouk Ta Kâmpông	0	100739	10494	80.6	0	0	0	1	0	0	0	horsesho
743	Kong Pluk (Pr.)	0	21600	14920	90.0	1							square
619	Khnâp (Pr.)	0	55208	10680	90.0	0	1	0	1	1	0	0	square
281	Kale Kalo (Pr.)	0	100210	6691	###	1	1	0	1	1	0	0	square
846	Anloung Chen	0	100657	21955	88.4	0	0	0	1	0	0	0	horsesho
175	Tuol Don Mov	0	100959	4168	90.0	0							horsesho
140	Kouk	0	200069	3260	84.0	0							horsesho
59	Kouk Komphneang	0	100056	1115	95.3	1	1	0	1	0	0	0	2causewa
436	Kouk Phlou	0	100469	8752	87.9	0							horsesho
567	Kouk Thbong	0	100103	10092	79.5	1	1	0	0	0	0	0	square
855	Kompong Phnom (Pı	0	100870	22327	88.5	1	1	0	1	1	0	0	square
259	Kouk Prasat Daeum	0	100531	6145	76.2	1	0	0	0	1	0	0	horsesho
483	Kouk Louk	0	100588	9018	80.6	1	0	0	0	1	0	0	horsesho
23	Kouk Kou	0	200040	458	###	0	1	0	1	1	0	0	horsesho
70	To Be Determined	0	200091	1402	90.0	1	1	0	0	0	0	0	2causewa
548	unnamed	0	100523	9711	84.3	1							horsesho
1077	Ta Phou (Pr.)	0	200735	28057	83.5	0							square
792	To Be Determined	0	200694	17443	82.8	1	0	0	1	0	1	0	square
997	Kouk Rœul	0	100947	26638	82.3	1	1	0	1	0	0	0	square
543	Koh Ho (Pr.)	733	51602	9674	87.5	0	0	1	0	1	0	0	horsesho
497	Krüs Châmbâk Thom	0	100529	9113	89.0	0	1	0	1	0	0	0	horsesho

Temple ID	name_english	Lustig ID	Archsite ID	Pelle ID	Azimuth	Principle Reservoir	Sandstone	Pink Sandstone	Laterite	Brick	Thmaphnom	other	Morphology
809	unnamed	0	100832	19164	###	0	1	0	0	1	0	0	square
27	Kouk Kou	0	200355	596	###	1	1	0	0	1	0	0	horsesho
1028	Tuol Kouk Pongro	0	100596	26676	90.0	1	1	0	1	1	0	0	square
687	Kouk Chas Mom	0	100090	13018	86.0	1							horsesho
673	Komnap Cangan Cro	0	200624	12143	89.6	0	0	0	0	1	1	0	horsesho
289	Kouk Phum Thom	0	200206	6845	84.7	1	1	0	1	0	0	0	horsesho
614	Prei Krauch North (P	0	200548	10646	84.0	1	1	0	1	1	0	0	horsesho
253	To Be Determined	0	200685	5990	###	0	1	0	0	1	1	0	square
34	Kouk Kou Trapeang I	0	200032	727	87.1	1	1	0	1	0	0	0	horsesho
399	Kong Hing (Pr.)	743	58102	8506	87.9	0	0	1	1	1	0	0	horsesho
720	Krol Romeas	728	47190	14184	88.9	0	0	0	1	0	0	0	circle
659	Kouk Ta Sao	0	100826	11560	###	0	1	0	0	0	0	0	square
275	Kouk Trapeang Svay	0	200191	6535	90.0	0	1	0	1	1	1	0	horsesho
681	Kouk Prasat Chkêvie	0	100475	12751	###	1	1	0	1	0	0	0	horsesho
248	Tuol Prasat Kouk Kh	0	100563	5853	65.7	0	1	0	1	0	0	0	square
509	Kouk Yeay Tea	0	100321	9301	###	1	1	0	1	1	0	0	horsesho
887	Preah Ngok	673	47902	23862	87.5	0	0	0	1	0	0	0	square
403	Balaing (Pr.)	0	100433	8544	88.0	1	1	0	1	1	0	0	horsesho
25	Kouk Chong Krom	0	200035	491	90.8	1	1	0	0	0	0	0	horsesho
765	Neang Kangrei (Pr.)	0	61502	16085	90.5	0	1	1	1	1	0	0	square
6601	DELETE		100429	11876	95.0	1							square
6999	To Be Determined		200553	3618		0							
40	Kouk Phluong	0	200422	821	90.0	1	0	0	1	0	0	0	horsesho
1	Tuol Kanhchan Kou	0	200007	11	90.3	0	1	0	0	1	0	0	horsesho
136	Kouk Pongro	0	200126	3182	90.0	1	1	0	0	1	0	0	horsesho
596	Kouk Tanor	0	200617	10205	98.5	0	1	0	0	0	0	0	square
705	Toul Ta Kong	0	200687	13283	###	0							square
487	Kouk Prasat (Pr.)	0	100648	9049	###	1	1	1	1	1	0	0	square
24	Kouk Ta Keo	0	200034	481	###	0							horsesho
202	Prey Prasat	0	200125	4553	90.0	0	1	0	1	1	0	0	horsesho
873	Tuol Krü	0	100779	23296	91.8	0	0	0	0	1	0	0	horsesho
418	Kouk Krüs	0	100497	8645	89.7	0	0	0	1	0	0	0	horsesho

Temple ID	name_english	Lustig ID	Archsite ID	Pelle ID	Azimuth	Principle Reservoir	Sandstone	Pink Sandstone	Laterite	Brick	Thmaphnom	other	Morphology
6	Kouk Don Rom	0	200005	41	###	1	1	0	1	0	0	0	horsesho
527	Kouk Ta Dan	0	100955	9497	69.3	0	0	0	1	0	0	0	square
1014	Kouk Dong 4	0	200640	26659	89.3	0	1	0	1	1	0	0	square
335	Phum Prasat	0	56800	7290	90.0	1	0	0	1	1	0	0	horsesho
159	Krâlok Rin	0	100878	3924	90.1	1	0	0	1	1	1	0	2causewa
114	Kou Kbal Roméas	0	100119	2834	89.9	1	1	0	0	1	0	0	2causewa
89	Kouk Dok Por	0	200468	1878	90.2	1	0	0	1	0	0	0	square
634	Kouk Ta Nuong	0	100912	11164	73.6	1	0	0	0	1	0	0	square
536	Kouk Ta Kahom	0	100733	9601	###	0	1	0	1	0	0	0	blob
325	Svay Sâ (Pr.)	0	56503	7124	83.2	0	0	0	1	1	0	0	horsesho
351	Kouk Kaun Sat	0	100330	7682	90.9	1	1	0	0	0	0	0	horsesho
633	Tuol Kânchân Bei	0	100867	11155	82.7	0							horsesho
416	unnamed	0	100516	8636	###	0	1	0	1	0	0	0	horsesho
1482	unnamed	0	100861	10376	90.6	0	1	0	0	0	0	0	square
358	Wat Trey Nhor	0	200302	7777	88.8	1	1	0	1	1	0	0	horsesho
823	To Be Determined	499	100885	20134	88.2	0	1	0	0	0	0	0	horsesho
165	Kroes (Pr.)	0	100557	3993	89.8	1	1	0	0	1	0	0	horsesho
747	Roeu (Pr.)	0	200731	14994	78.9	0							horsesho
868	buddhist terrace no	0	100780	23093	90.7	0	1	0	1	0	0	0	square
438	Prapis (Pr.)	0	50600	8763	86.4	1	1	0	0	1	0	1	horsesho
1018	Tuol Kouk Ta Chan	0	200641	26663	89.4	0	0	0	1	0	0	1	square
406	DELETE	0	200540	8561	82.2	1	1	0	1	1	0	0	horsesho
1448	To Be Determined	0	200658	6599	90.3	0	0	0	1	0	0	0	square
640	Wat Sanlong	0	56500	11381	90.0	1	1	0	1	1	0	0	2causewa
862	Kapilapura	512	49200	22798	85.0	1	0	0	1	1	0	0	4causewa
854	Kouk Dam Pnieu	0	200634	22325	87.2	0	1	0	1	1	0	0	horsesho
350	Tuol Yey Meas	0	200217	7671	71.3	0	1	0	0	0	0	0	horsesho
689	Kouk Ta Phiev	0	200691	13024	###	1							horsesho
1040	Kouk Hien	0	100662	26689	###	0	1	0	0	1	0	0	square
224	Kouk Kou Trapeang	0	100079	5332	90.0	1	1	0	1	0	0	0	horsesho
396	Trapeang Prasat	0	200001	8474	87.6	1	0	0	0	1	0	0	horsesho
560	DELETE	0	200510	10040	###	1							horsesho

Temple ID	name_english	Lustig ID	Archsite ID	Pelle ID	Azimuth	Principle Reservoir	Sandstone	Pink Sandstone	Laterite	Brick	Thmaphnom	other	Morphology
579	Neak Ta Kouk Ta Nea	0	200455	10140	67.4	1	1	0	1	0	0	0	square
671	Tuol Krüs	0	100172	12126	89.3	1							horsesho
842	Kouk Tuonléap	0	100678	21796	###	0	0	0	0	1	0	0	square
6811	Tuol Kon Yeang		200105	3144	90.0	0							square
398	Kouk Ping Puong	0	100367	8497	89.6	1	1	0	1	1	0	0	horsesho
430	Tuol Prasat	0	100591	8720	81.0	0	1	0	1	1	0	0	horsesho
154	Kouk Söng	0	100164	3733	90.0	1	0	0	0	1	0	0	horsesho
219	Kralang Sangke (Pr.)	0	65600	5287	81.5	1	0	0	0	1	0	0	horsesho
609	Tuol Kouk Roka	0	200315	10313	97.0	1							square
201	Kouk Soeng	0	60000	4540	75.4	1	1	0	0	1	0	0	horsesho
557	Kouk Ta Song	0	100881	9863	90.0	0	1	0	1	1	0	0	square
189	Trapeang Phlung (Pr.	0	60500	4290	86.9	1	1	0	1	1	0	0	horsesho
1359	DELETE	0	200140	1965	90.0	1							square
649	Kouk Ta Kuong (Pr.)	0	100467	11458	90.7	1	1	0	0	0	0	0	square
316	Tuol Trapeang Lboeu	0	100188	6986	88.5	1	1	0	0	1	0	0	horsesho
68	Kouk Yeay Seav (Pr.)	0	100964	1360	73.2	1	1	0	1	1	0	0	square
537	Tuol Snay Péak Néar	0	100735	9605	94.8	1	0	0	0	1	0	0	square
363	Kouk Ta Sin	0	100346	7937	90.1	1	0	0	1	1	0	0	horsesho
615	Prei Krauch South (P	0	50900	10648	84.3	1	1	0	1	0	0	0	horsesho
547	Trapéang Sâmrong	0	100453	9700	87.5	1	1	0	0	0	0	0	2causewa
160	Kouk Bos Chong	0	100961	3927	84.1	1	1	0	0	1	0	0	square
298	Phnom Bok (Pr.)	0	54700	6901	90.0	0	1	0	1	1	0	0	square
353	Tuol Ta Koav	0	100349	7705	90.0	1	1	0	1	0	0	0	horsesho
443	Prei Phdau (Pr.)	0	50300	8801	89.5	1	1	0	0	1	0	0	horsesho
230	Kouk Chan Laok	0	100029	5441	90.0	1	1	0	1	1	0	0	horsesho
102	Tuol Prasat Tumroer	0	100072	2186	75.0	1	1	0	0	1	0	0	horsesho
187	Kouk Trapeang Ponle	0	60700	4276	89.2	1	1	0	1	1	0	0	horsesho
861	To Be Determined	0	200631	22749	87.4	1	1	0	1	1	0	0	horsesho
590	Kouk Kanhchoan Chı	0	200503	10177	90.0	1	1	0	1	1	0	0	horsesho
179	Tuol Phom Preas Ko	0	100883	4181	91.3	0							square
5	Kouk Arak	0	200436	39	###	0	1	0	0	0	0	0	square
1088	DELETE	0	200101	3305	90.0	1							

Temple ID	name_english	Lustig ID	Archsite ID	Pelle ID	Azimuth	Principle Reservoir	Sandstone	Pink Sandstone	Laterite	Brick	Thmaphnom	other	Morphology
827	West Mebon	136	51700	20719	90.3	1	1	0	0	0	0	0	square
249	Tuol Prasat	0	100909	5885	89.0	1	1	0	0	0	0	0	2causewa
6774	Trapéang Krop Bay		100962	4188	85.9	1							horsesho
29	Kontrep (Pr.)	0	200027	616	###	1	1	0	1	1	0	0	horsesho
864	To Be Determined	0	200633	22936	83.8	1	1	0	1	0	0	0	square
589	Kouk Prasat (Pr.)	0	200499	10176	###	0	1	0	1	1	0	0	blob
444	Kouk Ampil	0	50400	8811	86.5	1							horsesho
638	Wat Tbaeng	0	55213	11375	90.0	0	0	0	0	1	0	0	square
167	To Be Determined	0	200184	4089	87.4	1	1	0	0	0	0	0	horsesho
650	Sramoch (Pr.)	0	100468	11464	87.7	0	1	0	1	1	0	0	horsesho
157	Tuol Sa Ouey	0	100995	3795	91.3	0							square
629	Kouk Prasat Don On	0	100952	10842	82.3	1	0	0	1	0	0	0	square
1050	Kouk Kâmnâp (Pr.)	0	100635	26704	99.8	0	1	0	0	1	0	0	square
594	Tuol Prasat Raka Oa	0	200547	10196	84.2	1	1	0	1	0	0	0	square
518	Kouk Kpuos	0	100437	9344	90.0	0	1	0	1	0	0	0	horsesho
11	Kouk Rosey	0	200476	170	96.7	1	1	0	1	1	0	0	horsesho
375	#N/A	0	200307	8264	###	1							horsesho
123	Kouk Trapeang Rong	0	200062	3022	72.9	1	0	0	1	0	0	0	square
575	Kouk Balang	0	200433	10127	98.0	0	1	0	1	0	0	0	square
817	Top (Pr.)	0	53900	19423	###	0	1	0	0	1	0	0	square
347	Kouk Tbong	0	100331	7616	89.5	1	0	0	0	1	0	0	horsesho
1022	unnamed	0	100865	26667	88.7	0	1	0	0	0	0	0	square
1056	Kouk Ta Tép	0	100127	26711	91.0	0	1	0	1	1	0	0	square
482	Kouk Kdei	0	100583	9011	88.5	0	1	0	1	1	0	0	square
422	Kouk Neak Ta	0	100599	8679	65.5	1	1	0	1	1	0	0	horsesho
1476	Tuol Ta Te	0	100853	9844	96.9	0	0	0	1	1	0	0	blob
670	Trapeang Koung	0	100211	12115	89.3	1	1	0	0	0	0	0	square
622	Tuol Chba Ta Poy	0	100966	10737	90.0	0	1	0	1	1	0	0	4causewa
752	Ta Ong (Pr.)	0	22600	15336	90.0	1							square
329	undetermined	0	100318	7170	###	1							square
198	DELETE	0	200172	4436	68.6	1							square
591	Kouk Beng (Pr.)	0	200535	10180	89.6	1	1	0	0	0	0	0	horsesho

Temple ID	name_english	Lustig ID	Archsite ID	Pelle ID	Azimuth	Principle Reservoir	Sandstone	Pink Sandstone	Laterite	Brick	Thmaphnom	other	Morphology
651	Tuol Kouk Putréa	0	100471	11469	90.0	1							horsesho
372	Chamrieng (Pr.)	0	57300	8235	83.3	1	1	0	1	0	0	0	horsesho
1063	Phnom Sruoch	553	55815	27133	90.0	0				1			square
1046	Kouk Arak	0	100665	26699	###	0	1	0	0	1	0	0	square
549	Kouk Trapeang Lopo	220	51800	9717	76.7	1	0	0	0	1	0	0	horsesho
247	Kouk Voeur Phom	0	100094	5852	69.5	1	1	0	0	0	1	0	square
197	Tuol Prasat Bos Knhe	0	200174	4412	90.4	0	0	0	1	1	0	0	horsesho
271	To Be Determined	0	200195	6468	76.0	1							horsesho
1039	Tuol Trapéang So	0	100647	26688	87.2	0	1	0	0	1	0	0	square
402	Kouk Prasat	0	54601	8534	88.0	0	0	0	0	1	0	0	horsesho
535	Méas Phos (Pr.)	0	100728	9598	95.9	0	1	0	1	0	0	0	horsesho
153	Tuol Kânchân Pong	0	100163	3720	90.0	1	0	0	0	1	0	0	2causewa
1065	Khting Slap (Pr.)	0	55806	27212	89.8	0							square
142	Phlov Prasat (Pr.)	0	100993	3314	90.0	0	1	0	1	1	0	0	horsesho
84	Kouk Kou Trapeang I	0	200063	1694	84.4	1	1	1	0	1	0	0	horsesho
495	Wat Loâk	0	100749	9098	86.9	0	1	0	0	0	0	0	square
564	#N/A	0	200486	10067	87.0	1	0	0	0	1	0	0	horsesho
326	Kouk Krous	0	100327	7134	85.6	1	1	0	1	1	0	0	horsesho
628	Kouk Prasat Don On	0	100950	10841	89.5	0	1	0	0	0	0	0	square
1467	Kouk Trâbèk	0	100748	9625	92.9	1	0	0	0	1	0	0	horsesho
1388	Kouk Oun Tey	0	100987	2547	89.5	1							square
1492	Kouk Khmoch	0	100606	11232	73.0	0							square
163	Kouk Ta Kong	0	100139	3971	89.3	1	1	0	0	0	0	0	horsesho
425	Kouk Moeun Duong	0	100601	8695	72.6	1	1	0	1	1	0	0	square
1523	Tuol Acha Mok	0	100679	21825		0							blob
357	Kouk Don Krim	0	200356	7747	90.0	0	1	0	1	0	0	0	horsesho
1484	Kouk Phnéo	0	100718	10444	94.3	0	1	0	0	0	0	0	blob
371	Kouk Ta Tet	0	200444	8203	###	1	1	0	1	0	0	0	square
413	Trâpeang Boeng (Pr.)	0	200560	8615	89.5	1	1	0	1	1	0	0	horsesho
1032	Trapeang Sen (Pr.)	0	100485	26680	91.6	1	1	0	1	1	0	0	square
563	#N/A	0	100020	10052	90.0	0	1	1	1	0	0	0	2causewa
190	Sras Chom Bok	0	100121	4293	90.0	0							2causewa

Temple ID	name_english	Lustig ID	Archsite ID	Pelle ID	Azimuth	Principle Reservoir	Sandstone	Pink Sandstone	Laterite	Brick	Thmaphnom	other	Morphology
151	Rông (Pr.)	0	200529	3598	85.8	1	1	0	1	1	0	0	horsesho
139	To Be Determined	0	200071	3256	58.3	1							square
625	Tuol Phum Krapeuw	0	200678	10804	80.8	0	0	0	1	0	0	0	square
33	Smonh (Pr.)	0	200029	703	80.3	1	0	0	1	1	0	0	2causewa
569	Kouk Khlang Han	0	200418	10108	###	1	0	0	1	0	0	0	horsesho
314	Wat Chas	0	100152	6973	90.0	1	1	0	1	1	0	0	2causewa
6573	DELETE		100361	13413	90.0	1							square
6755	DELETE		100892	12065	90.0	1							square
546	Kouk Kbal Romeas	0	100454	9687	91.2	1	1	0	1	1	0	0	horsesho
672	Toul Krus San Katchi	0	200681	12128	91.9	1	0	0	0	1	0	0	horsesho
112	Kouk Svay Chek	616	200429	2779	97.5	1	0	0	0	0	0	0	horsesho
1519	Kouk Chas	0	100600	21231		0	0	0	0	1	0	0	blob
819	Kouk Mokak	0	100767	19814	90.2	0	1	0	1	0	0	0	2causewa
260	He Phka (Pr.)	500	54406	6150	86.5	1	1	0	1	1	0	0	horsesho
859	To Be Determined	0	47182	22552	82.0	0	1	0	1	0	0	0	square
1360	Kouk Ta Tet	0	200469	1985	90.3	0	0	0	1	0	0	0	square
2	Kouk Daung	0	63100	17	###	1							horsesho
550	Kouk Yéay Tœum	0	100359	9736	88.5	0							square
382	Kouk Bos Thom	0	100108	8366	89.9	1							square
39	Tuol Prasat Kou	0	101009	805	90.0	1	1	1	0	1	0	0	2causewa
174	Lech (Pr.)	0	100958	4166	90.0	1	1	1	1	1	0	0	horsesho
660	Tuol Ta Phuong	0	100840	11568	82.2	0	1	0	1	0	0	0	horsesho
502	Phnom Krom (Pr.)	0	50100	9167	90.7	0	1	0	1	1	0	0	square
393	Wat Athvea	737	50000	8463	90.0	0	1	0	1	0	0	0	square
38	Arak Thalo	0	62700	781	86.8	0	0	1	1	1	0	0	horsesho
1475	Tuol A Sok	0	100849	9830	95.7	1	1	0	0	0	0	0	horsesho
1026	Kouk Lmong	0	100671	26673	90.9	0	1	0	0	0	0	0	square
1461	Kouk Ta But	0	100696	9059		1	1	0	0	1	0	0	blob
724	Ta Kâmnâp	0	100877	14272	86.3	1							2causewa
162	Tuol Neak Ta	0	100138	3964	78.8	1	0	1	0	1	0	0	horsesho
10	Tomrop (Pr.)	0	63000	86	75.9	1	1	0	1	1	0	0	horsesho
476	Kouk Krüs	0	100535	8968	89.2	0	1	0	1	1	0	0	horsesho

Temple ID	name_english	Lustig ID	Archsite ID	Pelle ID	Azimuth	Principle Reservoir	Sandstone	Pink Sandstone	Laterite	Brick	Thmaphnom	other	Morphology
1642	Khleang, north	774	48002	24009	89.4	0	1	0	1	0	0	0	square
865	Prei Phnom	0	100787	22954	76.0	0	1	0	0	1	0	0	2causewa
1479	Kouk Prasat Pô Bant	0	200556	10199	89.4	0							square
130	Pongro (Pr.)	0	61500	3089	86.3	1	1	0	0	1	0	0	horsesho
643	Wat Kralanh	0	63500	11403	85.5	1	1	1	1	1	0	0	horsesho
746	Svay Leu (Pr.)	296	22400	14973	88.8	1							
654	unnamed	0	100526	11499	90.0	1	1	0	1	1	0	0	horsesho
1389	DELETE	0	100986	2589		1							square
1438	Kouk Trapeang Treas	0	100106	5321	86.5	1							square
8	Seman Yung	0	62900	56	88.5	1	1	0	0	1	0	0	horsesho
593	Tuol Prasat Kouk Rak	0	200539	10192	70.4	0	1	0	1	0	0	0	square
1511	Kouk Pongro	0	100910	17207		1							blob
759	Banteay Srei (Pr.)	97	54602	15897	90.9	1	0	1	1	0	0	0	2causewa
209	Kouk Kom Nop	0	200451	4896	85.3	1	1	0	0	1	0	0	square
115	Kbal Khla (Pr.)	0	61900	2870	92.0	1	1	0	0	0	0	0	horsesho
621	Phnom Dei I	0	61300	10720	90.0	0	1	1	1	1	0	0	2causewa
211	Kouk Chan	0	100105	5003	89.4	1	1	0	0	0	0	0	square
414	Tuol Krüs (Pr.)	0	100416	8621	85.4	1	1	0	1	1	0	0	horsesho
835	Kouk Ta Svay	0	100580	21248	90.9	0	1	0	0	0	0	0	square
305	Prei Prasat	0	58905	6934	90.0	1	1	0	1	1	0	0	2causewa
152	Phnom Veak (Pr.)	0	55210	3610	89.4	1	1	1	1	1	0	0	square
79	Kou (Pr.)	0	200079	1542	85.3	1	1	0	0	1	0	0	horsesho
158	Kouk Pongro	0	100141	3857	90.0	1							horsesho
6015	Ta Nei (Pr.)		53200	18202	88.0	0	1	0	1	0	0	0	2causewa
16	Kouk Trapeang Chuk	0	200046	306	89.7	1	1	0	0	1	0	0	square
822	Kruos	0	100194	19998	85.8	0	1	0	1	0	0	0	square
45	#N/A	0	200488	920	90.0	0	1	0	0	1	0	0	square
904	Preah Palilay (Pr.)	714	47800	23919	88.0	0	1	0	1	0	0	0	horsesho
616	Patri (Pr.)	0	54402	10656	86.6	1	1	0	1	1	0	0	horsesho
307	Kouk Pô (Pr.)	586	59700	6944	###	0	0	0	0	1	0	0	horsesho
531	Kouk Ta Sok	0	100537	9557	95.3	1	0	0	1	0	0	0	square
473	Kandol Dom North (	126	58600	8948	93.9	0	0	0	0	1	0	0	horsesho

Temple ID	name_english	Lustig ID	Archsite ID	Pelle ID	Azimuth	Principle Reservoir	Sandstone	Pink Sandstone	Laterite	Brick	Thmaphnom	other	Morphology
571	Kouk Chan	0	200424	10115	91.7	1							horsesho
43	Kouk Kou	0	200054	896	95.1	1	1	1	1	1	0	0	horsesho
1073	Kouk Treang	0	58100	27581	88.8	1	0	0	0	1	0	0	horsesho
83	Wat Angkor Pheas	0	64700	1650	87.2	1	1	1	0	1	0	0	4causewa
646	Krüs Trâméng Thom	0	100441	11439	89.6	0	1	0	1	0	0	0	2causewa
228	Kouk Kou	0	200144	5415	91.7	0	1	0	0	0	0	0	horsesho
605	Kouk Trapeang Vean	0	200220	10298	###	1							horsesho
648	Réach Chântoal (Pr.)	0	100450	11452	90.0	1	1	0	1	1	0	0	horsesho
496	Trapéang Hos	0	100528	9108	89.6	1	1	0	0	1	0	0	horsesho
617	Tuol Trapéang Kou	0	100492	10665	89.5	1							horsesho
105	Wat Roluos	0	100355	2437	86.8	0	1	0	1	0	0	0	horsesho
3	Damnak Sdach	518	63200	27	###	0	0	0	0	1	0	0	horsesho
352	Kouk Tet	0	200211	7698	###	1							square
474	Kandol Dom South (	741	58700	8954	86.1	1	0	0	0	1	0	0	horsesho
257	Kouk Châk (Pr.)	502	54401	6137	88.2	1	0	0	0	1	0	0	horsesho
688	Kouk Yéay Ok	0	100741	13022	###	0	0	0	0	1	0	0	square
806	Kutishvara	0	53502	19104	89.8	0	0	0	1	1	0	0	horsesho
1524	To Be Determined	0	200618	22094	90.0	0	0	0	0	0	1	0	square
266	Wat Prey Preas	0	200090	6249	90.0	0	1	1	0	1	0	0	horsesho
1441	Borne	0	100935	5736	59.4	0							blob
412	Trapeang Thlok And	0	100373	8605	88.3	1	0	0	1	1	0	0	horsesho
424	Phum Prasat (Pr.)	0	59300	8689	75.4	1	1	0	0	1	0	0	horsesho
485	Kouk Ta Suos	0	100611	9037	###	1							square
498	Kânchân Thom	0	100449	9116	95.6	1	1	0	1	1	0	0	horsesho
1490	sans nom	0	100711	11116	98.3	0							blob
86	Prei Danghaeum (Pr.	0	59000	1753	90.0	1	1	0	0	1	0	0	horsesho
475	Olok (Pr.)	343	58402	8958	88.7	0							2causewa
493	Wat Prin	0	100517	9080	82.9	1	1	0	1	0	0	0	square
1041	Tuol Kouk Phéav	0	100667	26690	84.1	0	1	0	1	1	0	0	square
1474	Kânchân Ta Vèn	0	100844	9821	92.2	1							square
225	Wat Prasat	0	65302	5373	88.5	0	1	0	0	0	0	0	horsesho
1090	DELETE	0	200183	10854	86.3	1							

Temple ID	name_english	Lustig ID	Archsite ID	Pelle ID	Azimuth	Principle Reservoir	Sandstone	Pink Sandstone	Laterite	Brick	Thmaphnom	other	Morphology
1166	Kouk Ta Tei	0	100998	3741	80.8	0	1	0	0	0	0	0	
284	DELETE	0	100209	6717	95.0	1	1	0	1	1	0	0	horsesho
1357	DELETE	0	100064	1444	65.7	1							square
1497	Trapeang Véng	0	100513	11495	87.3	0	1	0	0	0	0	0	square
1471	Kouk Sâmbour	0	100994	9666	98.1	1							square
176	Andaung Kouk Ponle	0	100131	4173	92.9	0							horsesho
478	DELETE	0	100363	8987	91.5	1							horsesho
1721	Trapeang Srangè (Pr.	0	58800	8987	91.5	1	0	0	0	1	0	0	
428	Phum Prei Thom	0	50702	8714	88.5	0	1	0	1	1	0	0	blob
407	Kouk Ta Lonn	0	100401	8567	90.7	0							horsesho
264	Kouk Daung	544	58000	6184	91.1	1	0	0	0	1	0	0	horsesho
1496	Kouk Kânchân	0	100493	11475	85.5	0							horsesho
6965	Phum Phnov		200477	258		0	0	0	0	0	0	1	square
137	Tuol Konhchon Krola	0	200128	3213	91.6	1	1	1	0	1	0	0	horsesho
170	Kouk Ta Loun	0	100169	4114	90.0	1	1	0	0	1	0	0	horsesho
337	Tuol Trapeang Ta Se	0	100235	7369	90.0	1	1	0	1	1	0	0	horsesho
791	To Be Determined	0	100888	17442	84.3	1	0	0	1	0	0	0	horsesho
607	Kouk Chen Pou	0	200223	10301	80.0	1	1	0	0	1	0	0	horsesho
311	Kouk Kou Trapeang	0	100045	6958	89.2	0	1	0	1	1	1	0	horsesho
510	Kouk Chas	0	100375	9304	90.0	0	1	0	0	1	0	0	square
511	Trapéang Thnâl Bak	0	100406	9308	88.1	1	1	0	1	1	0	0	horsesho
320	Kouk Neak Ta	338	200218	7078	89.7	1	0	0	1	1	0	0	square
754	Trapéang Kânsèng	0	100884	15414	88.8	1	1	0	0	0	0	0	square
359	Kouk Wat	0	200431	7795	###	1	0	0	0	1	0	0	square
538	Tuol Kngôk	0	100746	9614	96.8	1	0	0	1	1	0	0	horsesho
282	Wat Pongro	0	100324	6694	90.0	1	1	0	1	0	0	0	square
262	Trapeang Totung Thı	0	57800	6168	91.1	1	0	0	0	1	0	0	horsesho
807	Bat Chum (Pr.)	692	53600	19128	90.0	1	0	0	0	1	0	0	horsesho
913	Terrace N°1	0	48800	23979	89.6	0	0	0	1	0	0	0	square
914	Khleang, south	323	48001	23983	89.2	0	1	0	1	0	0	0	square
584	Kouk Trapeang Ka T	0	200472	10160	###	0	0	0	0	1	0	0	square
246	Kos Kou (Pr.)	0	100455	5830	93.0	0							horsesho

Temple ID	name_english	Lustig ID	Archsite ID	Pelle ID	Azimuth	Principle Reservoir	Sandstone	Pink Sandstone	Laterite	Brick	Thmaphnom	other	Morphology
1513	To Be Determined	0	200708	19227	93.2	0							horsesho
644	Wat Roka	0	100356	11411	93.6	0	1	0	1	0	0	0	horsesho
188	Kouk Kuk	0	60600	4285	87.8	1	1	0	0	1	0	0	horsesho
7040	DELETE		200626	3063	90.0	0							square
692	Kompong Stung Prea	0	65500	13161	88.4	1	0	0	1	0	0	0	blob
173	Kouk Pongrô (Pr.)	287	52002	4157	91.0	1	0	0	1	1	0	0	horsesho
387	Kouk Leav	0	200133	8437	84.7	1	1	1	0	0	0	0	square
1500	DELETE	0	200537	12080		0	1	0	1	0	0	0	blob
1439	#N/A	0	200657	5518	89.6	0	0	0	0	0	1	0	square
645	Neak Ta Suos (Pr.)	0	100434	11429	89.4	0	1	0	1	1	0	0	2causewa
1019	Wat Prohm Kel	0	100785	26664	86.3	0	1	0	1	0	0	0	square
278	Kouk Kâmnâp	0	200505	6630	88.3	1	1	0	0	1	0	0	square
1454	To Be Determined	0	200309	8074	###	0	1	0	0	0	0	0	blob
690	Kouk Neak Ta	0	200432	13031	92.9	0	1	1	1	1	1	0	horsesho
1518	Kouk Thnot	0	100666	20980		0							blob
1363	DELETE	0	200047	357	96.5	0							square
99	Kouk Bos Andong	0	200460	2041	90.1	1	1	0	1	1	0	0	square
1432	DELETE	0	100982	3404	88.9	1							blob
1364	DELETE	0	200037	463		1							blob
1427	Kouk Ta Chom		100984	2692	###	0							square
1458	Kouk Arak	0	100607	9026	95.8	1							square
1466	DELETE	0	100730	9600	###	0							blob
6013	DELETE		53083	14451	92.0	0							square
145	Tuol Trapeang Kang	0	200528	3382	82.2	1	1	0	1	1	0	0	horsesho
1435	Kouk Ta Ros	0	100738	4030	87.6	0	0	0	0	1	0	0	blob
377	Prey Chor	0	200450	8274	91.3	0	1	0	0	0	0	0	square
1433	Kouk Chom Bok	0	100201	3532	###	1							blob
156	Samrong	51	61002	3790	88.7	1	0	0	1	0	0	0	horsesho
60	Tuol Prasat Kouk Toc	0	100063	1129	79.2	0	0	0	1	1	0	0	horsesho
1485	DELETE	0	100747	10500	86.1	0							blob
1470	Kouk Krânhoung	0	100759	9640	99.7	0	0	0	0	1	0	0	square
110	Kouk Kou	0	100975	2651	89.7	0	1	0	1	0	0	0	square

Temple ID	name_english	Lustig ID	Archsite ID	Pelle ID	Azimuth	Principle Reservoir	Sandstone	Pink Sandstone	Laterite	Brick	Thmaphnom	other	Morphology
365	Kouk Krous (Pr.)	0	100350	7969	90.3	0	1	0	1	0	0	0	horsesho
6004	Banteay Prei (Pr.)		52400	13234	88.0	0							horsesho
1505	Kouk Hing	0	100514	12582	90.3	0							square
348	Kouk Yeay Mau	0	100333	7635	93.5	1	1	0	1	1	0	0	horsesho
146	Kouk Kroes (Pr.)	0	200516	3398	90.0	1	0	0	1	1	0	0	horsesho
1521	#N/A	0	200904	21736		0							blob
637	To Be Determined	0	200668	11180	76.7	1	0	0	0	1	0	0	square
731	Sam Yot (Pr.)	0	21203	14531	90.0	1							2causewa
701	Toul Nokor Krau	0	200671	13245	86.8	0	1	0	1	0	0	0	square
721	Banteay Thom (Pr.)	651	52000	14233	87.0	1	1	0	1	0	0	0	horsesho
300	Leak Neang (Pr.)	658	54900	6908	89.4	1	0	0	0	1	0	0	square
81	Don Tei	0	61400	1580	91.7	0	0	0	1	0	0	0	square
67	Kbal Ansông (Pr.)	0	100113	1340	90.0	1	1	1	1	1	0	0	horsesho
178	Sralao (Pr.)	0	60900	4178	89.6	0	0	0	1	1	0	0	2causewa
481	Kouk Ta Préah	0	100573	9005	91.2	1							horsesho
810	Tuol Kbal Khmoch	0	100680	19197	91.1	1							2causewa
181	Bak (Pr.)	0	100875	4185	90.0	0	1	1	1	1	0	0	square
1430	DELETE	0	100972	3074	92.6	0	0	0	1	0	0	0	square
192	Thnu (Pr.)	0	60300	4333	83.4	1	1	0	0	1	0	0	2causewa
1468	Trapéang Sva Keüs	0	100751	9631	93.7	0	0	0	0	1	0	0	square
66	Kouk Chan (Pr.)	0	100111	1294	78.9	1	1	0	1	1	0	0	horsesho
315	Tuol Bromork	0	100187	6981	90.0	1							square
448	Kouk Ko	0	100726	8858	###	0	1	0	1	1	0	0	square
1514	Kouk Daun Ros	0	100399	19232		0							blob
522	Trapeang Svay (Pr.)	0	100451	9375	90.7	0	0	0	1	0	0	0	2causewa
1437	DELETE	0	100076	5043	###	0							square
1506	Kouk Loep	0	100866	12592	95.5	1							square
1477	Trapeang Ron Prey	0	200456	10141	73.1	1							square
488	Kouk Krâbao	0	100669	9050	90.4	0	0	0	0	1	0	0	horsesho
826	Prei (Pr.)	0	100447	20619	86.4	1	1	0	1	1	0	0	square
572	Kouk Sala Rien	0	200426	10118	91.8	0	1	0	0	0	0	0	square
726	Krüs Châmbâk Ma K	0	100645	14440	87.3	0	1	0	1	0	0	0	square

Temple ID	name_english	Lustig ID	Archsite ID	Pelle ID	Azimuth	Principle Reservoir	Sandstone	Pink Sandstone	Laterite	Brick	Thmaphnom	other	Morphology
1058	Bakong (Pr.)	434	58400	26716	89.8	1							2causewa
1504	Kouk Khpuos	0	100871	12390	###	1							blob
172	Prei Vihear (Pr.)	0	100965	4148	90.0	1	1	0	0	1	0	0	horsesho
1451	Kouk Tataok	0	200200	7307	70.2	1							square
1440	DELETE	0	100914	5731	86.8	0							square
814	Kravan (Pr.)	57	53700	19257	89.6	1	0	0	0	1	0	0	2causewa
1494	Kouk Roka	0	100695	11275		0	0	1	0	0	0	0	blob
376	Kanhchon Kantor	0	200447	8273	###	0	1	0	1	0	0	0	square
1517	To Be Determined	0	200650	20637	90.5	0							square
341	Kouk Chan	0	56900	7440	###	1	1	0	0	0	0	0	square
1459	Tuol Phnau	0	100683	9052		1							blob
525	Prei Monti (Pr.)	753	58200	9414	90.0	0	1	1	0	1	0	0	square
1526	DELETE	0	200616	27430		0							blob
6006	Ta Som (Pr.)		52800	17233	88.0	0	1	0	1	0	0	0	2causewa
477	DELETE	0	100369	8979	89.4	0							horsesho
830	Kouk Kpuos	0	100126	20877	89.0	1							4causewa
1481	Kouk Cheung	0	100854	10341	88.8	0							blob
1047	Wat Khnat	214	59200	26700	84.8	0	0	0	0	1	0	0	horsesho
339	Kouk Prasat Savatei	0	100311	7417	89.4	1	1	0	1	0	0	0	horsesho
1509	Tuol Kouk Chrés	0	100729	13075	###	0	1	0	1	1	0	0	blob
1473	DELETE	0	100727	9786		0							blob
1515	Trapéang Trach	0	100388	19452		0	0	0	1	0	0	0	blob
171	Kouk Krœul	0	100133	4127	90.0	0	1	0	0	0	0	0	square
1457	Kouk Sâmrong	0	100603	9023		0	0	0	1	0	0	0	blob
378	Phnom Dei	0	200208	8308	91.4	1	1	0	1	0	0	0	horsesho
409	Wat Kouk Chan Réar	0	100404	8579	88.5	1	1	0	0	1	0	0	horsesho
1021	Kouk Véang	0	100775	26666	86.7	0	1	0	1	1	0	0	square
562	Seman Teng (Pr.)	0	62800	10047	75.3	0	1	0	1	0	0	0	horsesho
1465	Kouk Deuk Pôr	0	100555	9572	###	0							blob
1493	DELETE	0	200541	11266	74.2	0							square
1719	Wat Kosaing	0	200692	7409	90.0	1							
717	Kouk Chas Mom	0	100091	13782	73.1	1	0	0	0	1	0	0	square

Temple ID	name_english	Lustig ID	Archsite ID	Pelle ID	Azimuth	Principle Reservoir	Sandstone	Pink Sandstone	Laterite	Brick	Thmaphnom	other	Morphology
1358	Kouk Ta Hun	0	200491	1628	89.3	1							blob
28	Phum Trapeang Pon	0	200060	609	71.9	0							square
789	Phnom Roung	0	59600	17367	90.0	0	0	0	0	1	0	0	square
1469	Kouk Trâ Méng Kông	0	100754	9637	88.9	0							blob
489	Wat Mechrey	0	100697	9061	77.0	1	1	0	1	1	0	0	square
1449	Kouk Ta Chun	0	100207	6633	90.4	0							square
263	Kouk Svay Pream	583	57900	6179	87.8	0	0	0	0	1	0	0	horsesho
345	Kouk Roka	0	200312	7574	###	1	1	0	0	0	0	0	square
630	Chbar Chek	0	100981	10846	88.8	0	0	0	1	0	0	0	square
1501	DELETE	0	100686	12171	80.9	0							blob
124	To Be Determined	0	200066	3024	78.9	1	1	0	0	0	0	0	square
1460	Trapeang Kouk Pong	0	100694	9057	###	1							square
191	Char (Pr.)	50	59800	4311	89.7	0	1	0	1	1	0	0	2causewa
1442	Kouk Chan (Pr.)	0	100937	5862	63.1	0	1	0	1	0	0	0	blob
1455	DELETE	0	200311	8196	###	1							square
627	To Be Determined	0	200023	10814	87.7	0							horsesho
366	Kouk Chouk Krohom	0	200425	7992	###	0	1	0	1	0	0	0	blob
1453	Wat Trey Nhor	0	200313	7576	###	0							square
265	Trapeang Phong (Pr.)	195	58300	6204	91.0	1	0	0	0	1	0	0	horsesho
1060	Balang (Pr.)	0	55605	26968	91.2	0	1	0	0	1	0	0	square
533	Kouk Chan	0	100723	9584	90.0	1	0	0	0	1	0	0	square
544	Prei (Pr.)	0	49701	9676	90.0	1							horsesho
472	Lolei (Pr.)	174	58900	8941	91.0	1							square
6579	Chapou Teng (Pr.)		100378	8600	90.0	1	1	0	0	1	0	0	horsesho
6791	Phom Chreas Kouk Konda		200153	5137	80.0	1	0	0	0	0	0	1	square
620	Banteay Ampil (Pr.)	0	56000	10681	81.4	0	1	0	1	0	0	0	horsesho
479	Preah Kô (Pr.)	167	58500	8991	90.0	0							horsesho
790	Wat Tuol Kpuos	0	100543	17404	98.9	0							square
1507	Kouk Roka	0	100547	12753	###	0							blob
658	Kouk Pongro	0	100681	11552	88.4	1	1	0	0	0	0	0	horsesho
1087	DELETE	0	200310	7900	###	1							
1527	Prei Sralao	0	200630	28030		0	0	0	1	0	0	0	blob

Temple ID	name_english	Lustig ID	Archsite ID	Pelle ID	Azimuth	Principle Reservoir	Sandstone	Pink Sandstone	Laterite	Brick	Thmaphnom	other	Morphology
6780	Trapeang Ampeo		100977	2717	89.0	1							square
1491	Tuol Angkanh	0	100848	11149	99.7	0	1	0	0	0	0	0	blob
796	Ta Keo (Pr.)	59	53300	18002	90.1	0	1	0	1	0	0	0	horsesho
5729	Batang		21700	14961	89.8	0							4causewa
1429	Kouk Chouk	0	100147	3015	90.0	1							square
180	Sampeou (Pr.)	0	61000	4184	88.7	0	0	0	1	0	0	0	square
1356	Prey Eat	0	100114	1357	73.6	0							square
1289	Kouk O Rolous	0	100938	12419		0							blob
1362	Kouk Chas	0	200478	286	90.5	1							blob
815	East Mebon	364	53100	19322	86.7	1	1	0	1	1	0	0	Square
1443	Borne	0	100931	5890	77.5	1							blob
818	Pre Rup (Pr.)	581	53800	19747	89.3	0							4causewa
317	Samrong (Pr.)	0	56602	7008	89.7	1	1	0	0	0	0	0	square
1390	DELETE	0	200131	2595	84.0	1							square
1428	Kouk Svay	0	100980	3007	90.1	1	1	0	1	0	0	0	square
890	Baphuon (Pr.)	603	47500	23883	89.5	0	1	0	1	0	0	0	horsesho
1502	Kouk Snuol	0	100685	12184	90.0	0							blob
1508	Toul Trapeang Chas	0	200212	13026	98.2	1							blob
1472	Sans nom	0	100946	9729		0							blob
131	Phnom Dei Kândal	0	100983	3119	88.2	0	1	1	0	0	0	0	square
128	Khpôp (Pr.)	0	61200	3068	89.8	0	1	0	1	1	0	0	square
6084	Neam Rup		59900	4381	82.82	1	1	0	0	0	0	0	2causewa
1462	Kouk Svay Chak	0	100609	9086		0							blob
788	Bayon (Pr.)	84	47400	17264	89.5	0	1	0	1	0	0	0	4causewa
6656	Wat Daun Chroam		100613	9085		0	1	0	0	1	0	0	blob
386	Cedei (Pr.)	0	50200	8424	91.3	1	0	0	1	1	0	0	2causewa
1277	DELETE	0	100500	11372	92.0	1							horsesho
203	Wat Ta Kam	0	100101	4614	75.7	1	1	0	0	0	0	0	square
891	Leper King Terrace	0	47702	23885	89.6	0	1	0	0	0	0	0	blob
530	Tuol Prasat	0	100532	9549	90.6	0	1	0	0	0	0	0	4causewa
885	Phnom Bakheng (Pr.	110	49600	23569	90.7	0	1	0	1	0	0	0	4causewa
1452	undetermined	0	100357	7526	###	0							square

Temple ID	name_english	Lustig ID	Archsite ID	Pelle ID	Azimuth	Principle Reservoir	Sandstone	Pink Sandstone	Laterite	Brick	Thmaphnom	other	Morphology
1512	Tuol Doeum Pô	0	100544	17466	86.7	1							blob
1447	DELETE	0	100956	6216	66.6	0							blob
6915	Sambour		200378	176		0							blob
1431	Ta Koy	0	100153	3402	###	0							blob
6920	Kouk		200384	411		0							blob
1632	Beng Mealea (Pr.)	763	21400	14919	89.0	1	1	0	1	0	0	0	4causewa
1613	Ta Prohm	558	53400	12151	88.7	1	1	0	1	0	0	0	2causewa
1483	Kouk Srok	604	57802	10399	###	0	1	0	0	0	0	0	blob
1495	Kouk Prâvas	0	100484	11472	80.3	1	1	0	0	1	0	0	horsesho
6677	Wat Thmei		100715	9088	###	0	1	0	0	0	0	0	square
6964	Roka		200474	124		0	0	0	0	0	0	1	blob
1463	Tuol Prei Châmkar	0	100408	9199	89.9	1	1	0	0	0	0	0	blob
6908	Lbaeuk		200371	460		0							blob
6688	remains of a channel		100758	8884	95.0	0	0	0	1	0	0	0	square
6914	Roka Leu		200377	5164		0							blob
6917	Roka Krom		200380	5182		0							blob
552	Prei Ta Ev	0	100760	9806	###	0	1	0	1	1	0	0	square
6962	Phum Damnak Sleng		200464	5369		0							blob
6913	Tumreung		200376	2144		0	0	0	0	0	0	1	blob
6956	Peak Sneang Chas		200448	1384		0							blob
36	Preah Khan	0	200024	761	82.4	0	0	0	0	1	0	0	square
603	Chau Srei Vibol (Pr.)	0	56400	10285	90.0	0	1	0	1	0	0	0	2causewa
886	Chrung (Pr.), southea	507	47201	23816	###	0	1	0	1	0	0	0	4causewa
1662	Angkor Wat	490	49700	22446	90.0	0	1	0	1	0	0	0	2causewa
6911	Reul		200374	2418		0							blob
6918	Kouk Kraol		200381	114		0	0	0	0	0	0	1	blob
6919	Toap Svay		200382	773		0	0	0	0	0	0	1	blob
6912	Chuk		200375	1185		0	0	0	0	0	0	1	blob
6909	Chuoy Chakrei		200373	908		0	1	0	1	0	0	0	blob
1093	#N/A	0	200757				0	0	1	1	0	0	
1094	#N/A	0	200727				0	0	0	0	0	1	
1100	DELETE	0	200571				0	0	1	1	0	0	

Temple ID	name_english	Lustig ID	Archsite ID	Pelle ID	Azimuth	Principle Reservoir	Sandstone	Pink Sandstone	Laterite	Brick	Thmaphnom	other	Morphology
1102	To Be Determined	0	200705				0	0	1	1	1	0	
1103	#N/A	0	200718				0	0	1	1	0	0	
1105	#N/A	0	200744				0	0	1	0	0	0	
1106	#N/A	0	200750				0	0	1	0	0	1	
1107	#N/A	0	200753				0	0	0	1	0	0	
1108	#N/A	206	200764				0	0	1	0	0	0	
1109	#N/A	0	200769				0	0	1	0	0	1	
1110	#N/A	0	200774				0	0	1	0	0	1	
1111	#N/A	0	200777				0	0	0	1	0	0	
1112	#N/A	0	200784				0	0	1	1	0	1	
1113	#N/A	0	200792				0	0	1	0	0	1	
1115	#N/A	0	200828				0	0	1	0	0	1	
1116	#N/A	0	200832				0	0	0	0	0	1	
1119	#N/A	0	23900				1	0	1	1	0	0	
1120	Ta Lei (Pr.)	0	52900				1	0	1	1	0	0	
1121	O Paong (Pr.)	0	55600				0	0	0	1	0	0	
1122	Damrei Krap (Pr.)	0	55800				0	0	1	1	0	0	
1123	Trapeang Rong (Pr.)	0	56410				1	0	1	1	0	0	
1124	Kong Bong (Pr.)	0	56411				1	0	1	1	0	0	
1125	O Ka-aek (Pr.)	498	58907				0	0	0	1	0	0	
1126	Neak Ta (Pr.)	0	60203				1	0	1	1	0	0	
1127	#N/A	0	100018				1	0	0	1	0	0	
1128	#N/A	0	100053				1	0	1	1	0	1	
1129	DELETE	0	200193				1	0	1	1	0	0	
1130	Tuol To Ong	0	200592				1	0	1	0	1	0	
1131	#N/A	0	200721				1	1	1	0	0	0	
1132	#N/A	0	200759				1	0	1	0	0	0	
1133	#N/A	0	200760				1	0	1	1	0	0	
1134	#N/A	0	200762				1	0	1	1	0	0	
1135	#N/A	0	200763				1	0	1	1	0	0	
1136	#N/A	0	200766				1	0	1	1	0	0	
1137	#N/A	0	200767				1	1	0	1	0	0	

Temple ID	name_english	Lustig ID	Archsite ID	Pelle ID	Azimuth	Principle Reservoir	Sandstone	Pink Sandstone	Laterite	Brick	Thmaphnom	other	Morphology
1138	#N/A	0	200810				1	0	1	0	0	0	
1139	#N/A	0	19200				0	0	0	1	0	0	
1140	Rong Lmong	0	47103				1	0	1	1	0	0	
1141	terrace and remains	0	47163				1	0	1	1	0	0	
1142	Chrung (Pr.), southw	505	47202				1	0	1	0	0	0	
1143	Chrung (Pr,), northe	511	47203				1	0	0	0	0	0	
1144	Chrung (Pr.), northw	508	47204				1	0	0	0	0	0	
1145	DELETE	0	54403				1	0	0	1	0	0	
1146	Anlong Thom (Pr.)	0	55403				1	0	0	1	0	0	
1147	Koki (Pr.)	0	55603				0	0	0	1	0	0	
1148	Rup Arak (Pr.)	0	55604				0	0	0	1	0	0	
1149	Rong Chen (Pr.)	0	55700				1	0	1	0	0	0	
1150	#N/A	0	64000				1	0	0	1	0	0	
1151	#N/A	0	69400				1	0	0	1	0	0	
1152	#N/A	516	81200				1	0	1	1	0	0	
1153	#N/A	514	81600				1	0	1	0	0	0	
1154	#N/A	0	81900				1	0	0	1	0	0	
1155	#N/A	129	100011				0	0	1	0	0	0	
1156	DELETE	0	100638				1	0	0	1	0	0	
1158	Koh Ta Meas	0	100698				1	0	1	1	0	0	
1159	unnamed	0	100699				1	0	1	1	0	0	
1160	unnamed	0	100700				1	0	1	1	0	0	
1161	unnamed	0	100701				1	0	0	1	0	0	
1162	Thmâ Chho	0	100702				1	0	1	1	0	0	
1163	unnamed	0	100703				1	0	1	1	0	0	
1164	unnamed	0	100704				1	0	0	1	0	0	
1165	Kos Mémay	0	100705				1	0	0	1	0	0	
1167	DELETE	0	200400				1	0	0	0	0	0	
1168	DELETE	0	200454				1	0	1	0	0	0	
1169	DELETE	0	200523				1	0	1	0	0	0	
1170	DELETE	0	200555				1	0	0	0	0	0	
1171	DELETE	0	200557				1	0	0	0	0	0	

Temple ID	name_english	Lustig ID	Archsite ID	Pelle ID	Azimuth	Principle Reservoir	Sandstone	Pink Sandstone	Laterite	Brick	Thmaphnom	other	Morphology
1172	DELETE		0	200561			1	0	1	0	0	0	
1173	DELETE		0	200562			1	0	1	0	0	0	
1174	DELETE		0	200563			1	0	0	0	0	0	
1175		#N/A	0	200715			1	0	1	1	0	1	
1176		#N/A	0	200723			1	0	1	1	0	0	
1177		#N/A	0	200748			1	0	1	0	0	0	
1178		#N/A	0	200755			1	0	0	0	0	0	
1179		#N/A	0	200770			1	0	1	1	0	1	
1180		#N/A	0	200772			1	0	1	1	0	0	
1181		#N/A	0	200786			1	0	1	1	0	0	
1182		#N/A	0	200797			1	0	1	0	0	0	
1183		#N/A	0	200798			1	0	1	0	0	0	
1184		#N/A	0	200799			1	0	1	0	0	0	
1185		#N/A	0	200800			1	0	1	0	0	0	
1186		#N/A	0	200801			1	0	1	0	0	0	
1187		#N/A	0	200802			1	0	1	0	0	0	
1188		#N/A	0	200803			1	0	1	0	0	0	
1189		#N/A	0	200804			1	0	1	0	0	0	
1190		#N/A	0	200805			1	0	1	0	0	0	
1191		#N/A	0	200817			1	0	1	1	0	0	
1192		#N/A	0	200818			1	0	0	1	0	0	
1193		#N/A	0	200819			1	0	1	0	0	0	
1194		#N/A	0	200821			1	0	1	1	0	0	
1195		#N/A	0	200823			1	0	1	0	0	0	
1196		#N/A	0	200827			1	0	1	0	0	1	
1197		#N/A	0	200834			1	0	1	0	0	1	
1198		#N/A	0	200837			1	0	0	1	0	1	
1199		#N/A	0	200839			1	0	0	0	0	0	
1200		#N/A	0	200840			1	0	1	0	0	0	
1201		#N/A	0	200841			1	0	1	0	0	0	
1202		#N/A	0	200842			1	0	1	0	0	0	
1203		#N/A	0	200843			1	0	1	0	0	0	

Temple ID	name_english	Lustig ID	Archsite ID	Pelle ID	Azimuth	Principle Reservoir	Sandstone	Pink Sandstone	Laterite	Brick	Thmaphnom	other	Morphology
1204	#N/A	0	200844				1	0	0	0	0	0	
1205	#N/A	0	200845				1	0	0	0	0	0	
1206	#N/A	0	200848				1	0	0	0	0	0	
1207	#N/A	0	200849				1	0	0	0	0	0	
1208	#N/A	0	200850				1	0	0	0	0	0	
1209	#N/A	0	200851				1	0	1	0	0	1	
1210	#N/A	0	200852				1	0	0	1	0	0	
1211	#N/A	0	200854				1	0	0	0	0	0	
1212	#N/A	0	200855				1	0	1	1	0	1	
1213	#N/A	0	200856				1	0	1	0	0	0	
1214	#N/A	0	200859				1	0	1	0	0	0	
1215	#N/A	0	200864				1	0	0	0	0	0	
1216	#N/A	0	200865				1	0	1	0	0	0	
1217	#N/A	0	200881				1	0	1	0	0	0	
1218	#N/A	0	200902				1	0	0	1	0	0	
1219	Bos Neak (Pr.)	0	55610				0	0	0	1	0	0	
1220	O Top (Pr.)	0	55802				0	0	1	1	0	0	
1221	#N/A	0	200749				0	0	0	1	0	0	
1254	Chup Chrei (Pr.)	0	55302				0	0	0	1	0	0	
1256	Trung Khla Khmum (	0	55503				0	0	1	1	0	0	
1258	Banteay	0	55622				0	0	1	1	0	0	
1260	Thmâ Dap (Pr.)	0	55702				0	0	0	1	0	0	
1262	Neak Ta (Pr.)	0	55900				0	0	0	1	0	0	
1284	Kos Krâmom	0	100706				0	0	1	0	0	0	
1303	Lop (Pr.)	0	200526				0	0	1	1	0	0	
1305	DELETE	0	200545				0	1	1	0	0	0	
1306	DELETE	0	200566				0	0	1	1	0	0	
1307	DELETE	0	200570				0	0	1	1	0	0	
1312	#N/A	0	200719				0	0	0	1	0	1	
1313	#N/A	0	200722				0	0	1	1	0	1	
1314	#N/A	0	200725				0	0	0	0	0	1	
1315	#N/A	146	200726				0	0	1	0	0	0	

Temple ID	name_english	Lustig ID	Archsite ID	Pelle ID	Azimuth	Principle Reservoir	Sandstone	Pink Sandstone	Laterite	Brick	Thmaphnom	other	Morphology
1316		#N/A	0	200743			0	0	1	0	0	1	
1318		#N/A	0	200747			0	0	1	1	0	1	
1319		#N/A	0	200751			0	0	1	1	0	1	
1320		#N/A	0	200752			0	0	1	1	0	1	
1321		#N/A	0	200754			0	0	1	0	0	0	
1322		#N/A	0	200756			0	0	1	0	0	1	
1323		#N/A	0	200758			0	0	1	0	0	0	
1324		#N/A	0	200765			0	0	0	1	0	0	
1327		#N/A	0	200773			0	0	1	0	0	1	
1328		#N/A	0	200775			0	0	0	0	0	1	
1329		#N/A	0	200776			0	0	0	1	0	1	
1330		#N/A	0	200778			0	0	0	1	0	0	
1331		#N/A	0	200779			0	0	0	1	0	1	
1332		#N/A	0	200787			0	0	1	1	0	1	
1334		#N/A	0	200811			0	0	0	1	0	0	
1336		#N/A	0	200824			0	0	0	0	0	1	
1337		#N/A	0	200826			0	0	1	1	0	1	
1339		#N/A	0	200831			0	0	1	1	0	1	
1340		#N/A	0	200835			0	0	1	0	0	1	
1349		#N/A	0	200900			0	0	1	0	0	0	
1350		#N/A	0	200901			0	0	0	1	0	0	
1351		#N/A	0	200903			0	0	1	1	0	0	
1534	Kouk Ta Ong		0	100864			1	0	0	0	0	0	
1555		#N/A	0	200720			0	0	0	1	0	1	
1560		#N/A	0	200795			0	0	0	0	0	1	
1562		#N/A	0	200833			1	0	0	0	0	0	
1568	Trapéang Sdei		0	100687			0	0	1	0	0	0	
1569	Trapéang Thlok Krâl		0	100693			1	0	1	0	0	0	
1571	Toul Ta Kwem		0	100731			1	0	1	0	1	0	
1572	Kouk Svay Khmao		0	100732			1	0	0	0	0	0	
1574	Tuol Prakhaing		0	100762			1	0	1	0	0	0	
1575	Kouk Roka		0	100773			1	0	0	0	0	0	

Temple ID	name_english	Lustig ID	Archsite ID	Pelle ID	Azimuth	Principle Reservoir	Sandstone	Pink Sandstone	Laterite	Brick	Thmaphnom	other	Morphology
1579	Kouk O Wat	0	100874				1	0	0	0	0	0	
1588	Trapeang Phnom De	0	200437				1	0	0	0	0	0	
1590	Tuol Prasat Prei Prea	0	200558				1	0	0	1	0	0	
1633	Poeng Ta Roet	0	22500				1	0	0	0	0	0	
1640	Royal Palace	0	47601				0	0	1	0	0	0	
1668	Wat Damnak	77	54500				1	0	0	0	0	0	
1669	Poeng Menoah	0	55618				0	0	1	0	0	0	
1672	DELETE	0	58902				0	0	0	1	0	0	
1677	DELETE	0	100039				1	0	0	0	0	0	
1681	#N/A		100060				1	1	1	1	1	1	
1682	undetermined	0	100067										
1683	DELETE	0	100083				0	0	0	1	0	0	
1684	DELETE	0	100084				1	0	0	0	0	0	
1685	DELETE	0	100087				0	0	1	0	0	0	
1686	DELETE	0	100089										
1688	Veal Sre Ta De	0	100112										
1689	DELETE	0	100115										
1692	undetermined	0	100228										
1696	DELETE	0	100351				0	0	1	0	0	0	
1704	unnamed	0	100882										
1705	DELETE	0	100951										
1706	DELETE	0	100976										
1707	DELETE	0	200012				1	1	1	1	0	0	
1708	Beng Khnar	0	200096										
1710	DELETE	0	200199				1	0	0	1	0	0	
1713	DELETE	0	200228				1	0	1	0	0	0	
1715	Me Tei	0	200519				0	0	1	0	0	0	
1716	Neak Ta Kouk Srok	0	200543				1	0	1	0	0	0	
1717	statue	0	200585				1	0	0	0	0	0	
1718	To Be Determined	0	200676				1	1	1	0	0	0	
1720	Toul Kohn Domrei	0	200704				0	0	1	0	0	0	
5932	Angkor Thom walls and m		47100				0	0	1	0	0	0	

Temple ID	name_english	Lustig ID	Archsite ID	Pelle ID	Azimuth	Principle Reservoir	Sandstone	Pink Sandstone	Laterite	Brick	Thmaphnom	other	Morphology
5934	Terrace K		47110				1	0	1	0	0	0	
5945	laterite steps		47132				0	0	1	0	0	0	
5949	Terrace F		47139				1	0	1	0	0	0	
5950	Terrace H		47141				1	0	1	0	0	0	
5964	ancient outlet of the East		47178				1	0	1	1	0	0	
5968	terrace		47185				1	0	1	0	0	0	
5971	Terrace N°3		47189				1	0	1	0	0	0	
5973	Rong Ta Dev		47205				0	0	1	0	0	0	
5974	stone blocks		47206				0	0	1	0	0	0	
5976	Victory Gate of Angkor T		47301				1	0	1	0	0	0	
5977	Gate of the Dead of Angk		47302				1	0	1	0	0	0	
5978	North Gate of Angkor Th		47303				1	0	1	0	0	0	
5979	West Gate of Angkor Tho		47304				1	0	1	0	0	0	
5980	South Gate of Angkor Th		47305				1	0	1	0	0	0	
5985	terrace		47406				1	0	0	0	0	0	
5988	Terrace of the Elephants		47700				1	0	0	0	0	0	
5989	Srah Ta Set		48003				0	0	1	0	0	0	
5991	Srah Andông		48005				0	0	1	0	0	0	
5997	Spean Thma		49100				1	0	0	0	0	0	
5999	DELETE		51402				0	0	0	1	0	0	
6007	Spean Veal Reach Dak		52802				0	0	1	0	0	0	
6010	stele shelter		53073				0	0	1	0	0	0	
6011	stele shelter		53074				0	0	1	0	0	0	
6012	stele shelter		53077				0	0	1	0	0	0	
6014	stele shelter		53084				1	0	1	0	0	0	
6016	Srah Srang		53503				0	0	1	0	0	0	
6018	Srah Phong		53802				0	0	1	0	0	0	
6019	Krol Romeas		54200				0	0	1	0	0	0	
6020	Spean Tor		54302				0	0	1	0	0	0	
6029	Preah Noreay		55207	28069	90.0	0							square
6031	Preah Thom		55300				1	0	0	0	0	0	
6047	DELETE		200574				1	0	1	1	0	0	

Temple ID	name_english	Lustig ID	Archsite ID	Pelle ID	Azimuth	Principle Reservoir	Sandstone	Pink Sandstone	Laterite	Brick	Thmaphnom	other	Morphology
6048	Spean Thmâ Stung Toch		56300				1	0	0	0	0	0	
6050	Stung Chas		56403				0	0	1	0	0	0	
6074	Spean		57705				0	0	1	0	0	0	
6077	Spean O Ka-aek		58904				0	0	1	0	0	0	
6080	Wat Bo		59302				1	0	0	0	0	0	
6082	Spean Thmâ		59500				0	0	1	0	0	0	
6090	Tuol Yeay Nâk		61503				1	0	1	1	0	0	
6092	Kouk Kbal Krapeu		62400				1	0	1	0	0	0	
6093	Lovea		62402				0	0	1	0	0	0	
6094	Spean Thmâ Stoeng Chkk		62600				0	0	1	0	0	0	
6114	Spean Stung Preah Srok		65300				0	0	1	0	0	0	
6413	DELETE		100033				1	0	0	0	0	0	
6416	Bam Penh Reach		200619				0	0	1	0	0	0	
6425	Neak Ta Trapeang Snol		100122				1	0	0	0	0	0	
6444	Srè Ta Ôk		100178				0	0	1	0	0	0	
6466	Prey Kop Bos Thom		100217				0	0	1	0	0	0	
6469	DELETE		100220				1	0	1	0	0	0	
6473	Kouk Ta Keo		100226				1	0	0	1	0	0	
6571	Wat Bantéay Rüssei		100358				1	0	0	0	0	0	
6580	Lolei Baray		100379				0	0	1	0	0	0	
6585	DELETE		100386				1	0	1	0	0	0	
6588	DELETE		100394				1	0	0	0	0	0	
6590	Trapéang Khmoch		100396				1	0	0	1	0	0	
6591	Kouk Phnéao		100397				0	0	0	1	0	0	
6607	DELETE		100440				0	0	1	0	0	0	
6620	Wat Chânlông		100474				1	0	0	0	0	0	
6621	DELETE		100481				1	0	0	0	0	0	
6633	Srah Andaung Préng		100520				0	0	1	0	0	0	
6642	Trapeang Veng		100551				0	0	1	0	0	0	
6644	Kouk Roka		100553				0	0	1	0	0	0	
6664	DELETE		100649				1	0	0	0	0	0	
6667	Wat Po Langka		100656				1	0	1	0	0	0	

Temple ID	name_english	Lustig ID	Archsite ID	Pelle ID	Azimuth	Principle Reservoir	Sandstone	Pink Sandstone	Laterite	Brick	Thmaphnom	other	Morphology
6668	Wat Sérei Vuong		100659				1	0	1	0	0	0	
6669	Wat Svay		100682				1	0	0	0	0	0	
6675	Wat Kampong Phluk		100713				1	0	0	0	0	0	
6687	Tuol Ta Trao		100757				0	0	0	1	0	0	
6694	DELETE		100782				0	0	1	0	0	0	
6696	Remains of canalisation		100786				0	0	1	0	0	0	
6737	unnamed		100833				1	0	0	1	0	0	
6741	southeast corner of the L		100838				0	0	1	0	0	0	
6742	Tuol Thnâl Ansong		100839				0	0	1	1	0	0	
6749	DELETE		100863				0	0	0	1	0	0	
6751	unnamed - not found		100869				1	0	1	1	0	0	
6760	DELETE		100902				0	0	1	0	0	0	
6765	Laterite Boundary Stone		100911				0	0	1	0	0	0	
6768	Spean Thmâr		100939				0	0	1	0	0	0	
6779	Kanh Chon Thkov		100974				1	0	0	0	0	0	
6786	DELETE		101008				1	0	1	0	0	0	
6846	Spean Anlong La-âk		200011				1	0	0	0	0	0	
6901	Spean Phneak Dai		200357				0	0	1	0	0	0	
6902	Spean Thmâ		200358				0	0	1	0	0	0	
6903	Spean Khla Kon		200359				0	0	1	0	0	0	
6904	Trâpeang Spean		200360				0	0	1	0	0	0	
6916	Kouk Chan		200379				1	1	1	0	0	0	
6948	Spean Thma		200420				0	0	1	0	0	0	
6949	Spean Thmar Toch		200421				0	0	1	0	0	0	
6950	Spean		200423				0	0	1	0	0	0	
6951	Spean Trapeang Chouk		200427				0	0	1	0	0	0	
6958	Spean Yey Lek		200452				0	0	1	0	0	0	
6959	Wat Roka		200457				1	0	1	0	0	0	
6960	Neak Ta Trapeang Dok Po		200459				1	0	1	0	0	0	
6961	Spean Thma Chas		200462				0	0	1	0	0	0	
6963	Spean Thma Chas		200466				0	0	1	0	0	0	
6970	Neak Ta Dem Por		200493				1	0	0	0	0	0	

Temple ID	name_english	Lustig ID	Archsite ID	Pelle ID	Azimuth	Principle Reservoir	Sandstone	Pink Sandstone	Laterite	Brick	Thmaphnom	other	Morphology
6971	DELETE		200603				0	0	1	1	0	0	
6972	Spean		200501				0	0	1	0	0	0	
6973	Wat Ko Kir		200504				1	0	0	0	0	0	
6975	Spean Thmâ Bay Kriem		200508				1	0	1	0	0	0	
6980	Spean		200514				0	0	1	0	0	0	
6981	Spean		200515				0	0	1	0	0	0	
6982	Krâlik Krâlok		200518				0	0	1	0	0	0	
6986	Chak Chroal (Pr.)		200524				0	0	1	0	0	0	
6990	Spean Thom		200532				1	0	1	0	0	0	
6991	Trâpeang Russei		200533				0	0	0	1	0	0	
6994	Tuol Yeay Nong		200542				1	0	0	0	0	0	
6996	DELETE		200549				1	0	1	0	0	0	
7001	DELETE		200559				0	0	0	1	0	0	
7002	DELETE		200564				1	0	0	1	0	0	
7004	Spean O Chen Chien		200568				0	0	1	0	0	0	
7005	DELETE		200569				1	0	1	0	0	0	
7011	Tamnup Ang Teuk		200583				0	1	1	0	0	0	
7034	bridge		200615				0	0	1	0	0	0	
7038	To Be Determined		200623				0	0	1	0	0	0	
7045	road foundation		200646				1	0	1	0	0	0	
7047	sandstone block		200648				1	0	0	0	0	0	
7050	#N/A		200669				1	0	0	1	0	0	
7054	Wat Kaun Sat		200689				1	0	0	0	0	0	
7057	To Be Determined		200701				1	0	0	0	0	0	
7058	baray outlet		200706				1	0	1	1	1	0	
7059	Kouk Phneao 2		200709				0	0	0	1	0	0	
7060	Kouk Phneao 3		200710				0	0	0	1	0	0	
7061	Kouk Phneao 4		200711				1	0	0	1	0	0	
7062	Kouk Phneao 5		200712				1	0	0	1	0	0	
7074	#N/A		200781				1	0	0	0	0	0	
7075	#N/A		200782				1	0	0	0	0	0	
7076	#N/A		200783				1	0	0	0	0	0	

Temple ID	name_english	Lustig ID	Archsite ID	Pelle ID	Azimuth	Principle Reservoir	Sandstone	Pink Sandstone	Laterite	Brick	Thmaphnom	other	Morphology
7081		#N/A	200796				1	0	0	0	0	0	
7082		#N/A	200806				0	0	1	0	0	0	
7083		#N/A	200807				0	0	1	0	0	0	
7084		#N/A	200808				1	0	0	0	0	0	
7085		#N/A	200809				0	0	1	0	0	0	
7091		#N/A	200829				1	0	1	0	0	0	
7092		#N/A	200830				0	0	1	0	0	1	
7094		#N/A	200847				1	0	0	0	0	0	
7096		#N/A	200860				0	0	1	0	0	0	
7098		#N/A	200862				1	0	0	0	0	0	
7099		#N/A	200866				0	0	1	0	0	0	
7100		#N/A	200867				0	0	1	0	0	0	
7101		#N/A	200868				1	0	0	0	0	0	
7102		#N/A	200869				1	0	0	0	0	0	
7103		#N/A	200870				1	0	0	0	0	0	
7104		#N/A	200871				1	0	0	0	0	0	
7113		#N/A	200882				1	0	1	0	0	0	
7114		#N/A	200883				1	0	0	0	0	0	
7115		#N/A	200884				1	0	0	0	0	0	
7116		#N/A	200885				1	0	0	0	0	0	
7117		#N/A	200886				1	0	0	0	0	0	
7118		#N/A	200887				1	0	0	0	0	0	
7121		#N/A	200894				0	0	1	0	0	0	

Temple ID	Pedestal	Area	Moat
876		90.9	0
874		121.1	0
878		144.9	0
933		182.5	0
973		185.5	0
968		205.1	0
965		215.1	0
952		220.1	0
956		226.3	0
1015		229.4	0
1069		232.0	0
877		241.8	0
940		247.2	0
958		259.4	0
542		259.9	0
912		264.0	0
832		287.4	0
949		290.6	0
989		292.3	0
978		303.6	0
302		353.6	0
875	A2	353.7	0
1618	A4	357.4	0
969		358.6	0
635		379.4	0
976		381.0	0
936		385.8	0
526	e (ea	396.5	1
1048		410.0	0
685		419.2	0
884		421.6	0
1016		424.6	0

Temple ID	Pedestal	Area	Moat
111	e (ea	426.1	1
908		426.8	0
944		430.0	0
794		431.2	0
941		431.8	0
1007		435.7	0
990		438.8	0
1061		443.5	0
704	e (ea	452.3	1
977		481.6	0
938		485.7	0
702	e (ea	489.9	1
883		491.5	0
321	e (ea	496.2	1
711	e (ea	500.2	1
1076		504.8	0
1045		506.9	0
1078		520.0	1
484	e (ea	528.8	1
985		531.0	0
723		531.0	0
706		549.3	1
960		550.5	0
937		551.8	0
1480		556.8	0
1489		557.8	1
161	e (ea	560.7	1
1067		563.2	0
287	e (ea	569.0	1
582	e (ea	586.6	1
44	e (ea	595.0	1
999	e (ea	596.4	1

Temple ID	Pedestal	Area	Moat
1000		597.4	0
1004		597.4	0
839	e (ea	604.3	1
663		607.5	0
585	e (ea	607.5	1
54	A2	610.9	1
1002		617.0	0
274		626.3	0
799		627.8	0
675	e (ea	633.3	1
1017		634.3	0
94	A1	635.8	1
255		655.2	0
1082		668.0	0
1487	e (ea	668.6	1
299		671.3	0
824		671.5	1
254		671.7	0
470	e (ea	675.8	1
902		675.9	0
763	e (ea	684.0	1
987		684.9	0
360	e (ea	692.5	1
97	e (ea	696.5	1
56	e (ea	698.0	1
677		698.4	1
866		699.3	0
836	e (ea	710.5	1
41	e (ea	711.4	1
491		721.0	0
1226	e (we	721.6	1
50	e (ea	724.4	1

Temple ID	Pedestal	Area	Moat
1033		727.3	1
415	e (ea	728.1	1
853		734.1	0
656	e (ea	735.3	1
927		741.9	0
88		743.9	1
707	e (ea	746.7	1
1003		749.2	1
983		750.5	0
1011		751.1	0
1525		751.1	0
384	e (ea	752.3	1
231	e (ea	756.3	1
771		757.7	1
843	e (no	757.8	1
53	e (ea	762.6	1
134	e (ea	765.5	1
935		769.0	0
583	e (ea	770.0	1
710	e (ea	771.4	1
1055		772.6	0
942		777.6	0
306		777.7	0
98	A3	780.2	1
1072		784.0	0
427	e (ea	784.3	1
1035		785.0	0
709		799.7	0
841	e (ea	802.0	1
239	e (ea	802.5	1
636		803.8	0
1029		810.4	0

Temple ID	Pedestal	Area	Moat
736		814.3	1
948		815.6	0
725	e (ea	816.1	1
1436		816.9	0
722	e (ea	818.6	1
556	e (ea	819.9	1
623	e (we	827.8	1
837	e (ea	829.4	1
631	e (ea	832.1	1
313	e (ea	832.7	1
833	e (ea	835.5	1
910		835.8	0
1025		843.3	0
820		847.2	0
613	e (ea	848.6	1
276	e (ea	852.0	1
1036		854.2	0
420	e (ea	854.2	1
364	e (ea	854.3	1
761		855.4	0
241	e (ea	856.3	1
541		859.1	1
46	e (ea	859.7	1
1661		861.0	0
577	e (ea	863.1	1
220	e (ea	864.8	1
554	e (ea	865.4	1
996		870.2	0
703	e (ea	875.2	1
120	e (ea	877.4	1
580	e (ea	879.0	1
1450		884.8	1

Temple ID	Pedestal	Area	Moat
1010		886.6	1
437	ie (ea	894.2	1
600		894.7	0
309	ie (ea	903.8	1
504	ie (ea	905.7	1
540	ie (ea	906.4	1
844	ay	906.9	1
655	ie (ea	907.2	1
860		910.8	0
1043	ie (ea	911.4	1
215	A1	917.6	1
610	ie (ea	923.5	1
756		924.3	0
793		925.2	0
513		927.8	1
490		932.3	0
184		933.4	0
283	ie (ea	933.6	1
92	ie (ea	937.8	1
639		938.6	1
684	ie (ea	938.7	1
344	A1	941.1	1
932		950.1	0
204	ie (ea	953.0	1
939		955.7	0
905		960.1	0
433	ie (ea	960.9	1
595	ay	963.0	1
718	ie (ea	967.2	1
442		973.0	0
850	ie (ea	973.6	1
1079		982.8	0

Temple ID	Pedestal	Area	Moat
109	e (ea	983.8	1
695	e (ea	984.5	1
519	e (ea	993.0	1
240		993.7	1
431	e (ea	1000.5	1
400	e (ea	1005.8	1
486	e (ea	1006.8	1
573	e (ea	1010.7	1
322	e (ea	1010.9	1
227	e (ea	1014.5	1
223	ay	1017.7	1
506	e (ea	1025.6	1
100	e (ea	1027.8	1
662		1031.7	0
1030		1033.0	0
445	e (ea	1033.2	1
508	e (ea	1033.6	1
766	e (ea	1035.0	1
419	e (ea	1037.1	1
440		1042.5	0
6678	e (ea	1048.7	1
118	e (ea	1049.2	1
611	e (ea	1052.2	1
1005		1053.2	0
1456		1055.7	0
397	ay	1055.8	1
390	e (ea	1058.8	1
507	e (ea	1063.8	1
1071		1064.5	0
697		1066.2	0
576		1066.6	0
872		1066.9	0

Temple ID	Pedestal	Area	Moat
199	A1	1072.3	1
776	e (ea	1080.1	1
361	e (ea	1084.4	1
906		1085.8	0
469	e (ea	1089.5	1
55	B	1095.6	1
125	e (ea	1096.2	1
122	e (ea	1096.2	1
121	e (ea	1096.2	1
6432	e (ea	1096.2	1
116	ay	1098.1	1
236	e (ea	1103.7	1
657		1110.7	0
1075		1117.4	1
250		1122.6	0
434	e (ea	1128.8	1
7	e (ea	1129.8	1
104	e (ea	1129.8	1
1042		1131.3	0
280	e (ea	1132.8	1
592	e (ea	1133.4	1
1057		1134.9	0
586	e (ea	1139.6	1
380	A1	1142.6	1
51	e (ea	1144.9	1
730		1145.3	1
528	e (ea	1145.6	1
58		1147.7	1
838	e (ea	1149.4	1
782		1150.8	0
566	e (ea	1154.5	1
512	e (ea	1156.2	1

Temple ID	Pedestal	Area	Moat
970		1159.4	0
713		1159.6	0
1027		1162.0	0
93	e (ea	1163.3	1
812	e (ea	1164.7	1
1659		1165.2	0
558	e (ea	1167.3	1
147	e (ea	1168.4	1
64	e (ea	1169.2	1
813	e (ea	1171.4	1
930		1171.8	0
553	e (ea	1172.0	1
888		1172.1	0
561		1177.3	0
618	e (ea	1183.5	1
1013		1185.7	0
957		1186.6	0
13	e (ea	1188.9	1
851	e (no	1198.2	1
391	e (ea	1199.7	1
226	e (we	1201.8	1
811	e (ea	1205.0	1
405	e (ea	1206.4	1
346	e (no	1208.6	1
432	e (ea	1211.6	1
237	e (ea	1215.5	1
14	e (ea	1216.6	1
292		1220.4	1
193	e (ea	1225.3	1
641	e (ea	1226.6	1
816	e (ea	1228.7	1
840	e (ea	1231.7	1

Temple ID	Pedestal	Area	Moat
108	e (ea	1233.7	1
252	e (ea	1235.8	1
77	e (ea	1235.8	1
492		1236.0	0
383	e (ea	1237.0	1
1445		1238.7	0
349	e (ea	1244.7	1
72		1245.9	0
62	e (ea	1246.5	1
183	e (ea	1253.8	1
708	e (ea	1258.8	1
598	e (ea	1262.6	1
1083		1266.9	0
825	ay	1268.3	1
6554		1275.2	0
665		1280.0	0
91	e (ea	1294.1	1
343	e (ea	1299.6	1
719	e (ea	1303.0	1
565	e (ea	1307.3	1
295	e (ea	1308.4	1
18	A4	1309.0	1
31		1309.5	0
208	ay	1311.9	1
270	e (ea	1312.7	1
1054		1313.0	0
1091		1316.1	0
312	A1	1317.9	1
517		1331.9	0
597	e (ea	1334.9	1
269		1336.2	1
318		1336.6	1

Temple ID	Pedestal	Area	Moat
293		1336.9	0
404	e (ea	1341.9	1
676	ay	1341.9	0
500	e (ea	1342.0	1
234	e (ea	1343.4	1
279	e (ea	1345.2	1
244	e (ea	1351.4	1
774	e (ea	1352.0	1
32		1353.6	1
85	ay	1355.3	1
87	e (ea	1362.4	1
381	e (ea	1363.4	1
599	e (ea	1363.5	1
661	e (ea	1366.6	1
42	e (ea	1371.6	1
664	e (ea	1377.1	1
354	A4	1378.9	1
49	e (ea	1380.9	1
149	e (ea	1381.6	1
333	e (ea	1381.6	1
1009	e (ea	1381.8	1
330	e (ea	1383.5	1
686	e (ea	1383.8	1
52	A1	1385.8	1
1020		1385.9	1
1023		1386.2	0
30	e (ea	1391.0	1
604	e (ea	1392.5	1
773	ay	1393.1	1
297	e (ea	1400.6	1
369	e (ea	1405.6	1
1070		1407.5	0

Temple ID	Pedestal	Area	Moat
728		1409.2	0
285	ay	1410.3	1
574		1412.4	0
845	e (ea	1412.6	1
304	e (ea	1413.3	1
138		1416.9	1
74	ay	1421.8	1
216	e (ea	1422.4	1
17	e (ea	1428.0	1
1498		1428.7	0
47	e (ea	1429.6	1
222	ay	1429.6	1
12		1431.2	1
272	e (ea	1434.1	1
998		1435.9	0
334	e (ea	1439.9	1
480	e (ea	1441.1	1
296	e (ea	1445.1	1
6847		1445.6	0
362	e (ea	1447.0	1
578	e (ea	1448.7	1
570	e (ea	1458.4	1
26	e (ea	1458.6	1
602	e (ea	1463.3	1
232	ay	1464.4	1
426	e (ea	1464.8	1
1510		1466.7	0
1434		1468.4	0
332	e (ea	1469.4	1
781	e (ea	1470.9	1
214	e (ea	1472.6	1
101	e (ea	1476.5	1

Temple ID	Pedestal	Area	Moat
1006		1477.0	0
308	A1	1478.1	1
323	B	1479.2	1
103	ay	1479.8	1
356	e (ea	1483.6	1
221	e (ea	1484.7	1
268		1485.4	0
1049		1487.6	0
166	e (ea	1490.7	1
1516		1495.1	0
652	e (ea	1495.8	1
770	e (ea	1496.2	1
277	e (ea	1497.3	1
340	e (ea	1506.1	1
7049	e (ea	1515.1	1
829		1517.3	1
229	e (ea	1521.1	1
132	e (ea	1530.4	1
750		1532.6	1
235	A3	1535.5	1
529	e (ea	1540.5	1
127	e (ea	1540.7	1
955		1546.0	0
532	e (ea	1546.8	1
499	e (ea	1547.2	1
218	A	1549.6	1
69	e (ea	1550.2	1
7021		1551.3	0
96	e (ea	1554.1	1
1037		1554.5	0
870	e (ea	1556.3	1
417	e (ea	1559.9	1

Temple ID	Pedestal	Area	Moat
233	e (ea	1564.4	1
642	e (we	1564.8	1
1008		1564.9	0
1031		1565.2	0
6005	e (east	1566.1	1
245		1567.4	0
1499		1569.4	0
762		1569.6	1
20	e (ea	1571.6	1
22	e (ea	1572.0	1
515	e (ea	1574.3	1
909		1576.9	0
19	A2	1579.3	1
186	e (ea	1580.7	1
291	e (ea	1580.8	1
319	ay	1588.1	1
551	e (ea	1589.6	1
126	e (ea	1594.2	1
520	e (ea	1599.4	1
267	e (ea	1603.4	1
768	e (no	1624.0	1
669	e (ea	1625.2	1
647	e (ea	1626.7	1
76	e (ea	1628.6	1
6685		1630.1	0
734	e (no	1632.9	1
797	ay	1634.9	1
401	e (ea	1636.5	1
392	e (ea	1642.2	1
212	e (ea	1643.2	1
1478		1647.2	0
408	e (ea	1649.4	1

Temple ID	Pedestal	Area	Moat
310	A4	1653.5	1
410	e (ea	1660.8	1
57	e (ea	1662.7	1
849	e (ea	1666.7	1
4	e (ea	1667.2	1
435	e (ea	1673.0	1
144	e (ea	1674.7	1
1095		1676.5	0
729	e (ea	1677.1	1
555	e (ea	1678.0	1
164	e (ea	1678.9	1
205	e (ea	1688.6	1
559	e (ea	1696.4	1
447	e (ea	1699.2	1
217	e (ea	1701.5	1
716	e (ea	1704.6	1
501	ay	1708.3	1
61	e (ea	1713.4	1
1522		1720.7	0
256	e (ea	1722.1	1
290	e (ea	1724.5	1
338	e (ea	1726.0	1
95	ay	1728.8	1
48	e (ea	1729.1	1
368	e (ea	1730.7	1
1053		1736.4	0
608		1738.8	0
374	e (ea	1740.4	1
871	e (ea	1744.8	1
421		1754.7	1
242	e (ea	1755.8	1
524	e (ea	1758.8	1

Temple ID	Pedestal	Area	Moat
848	e (ea	1761.0	1
78	e (ea	1761.2	1
177		1761.8	0
148	e (ea	1764.5	1
696		1766.0	0
514	e (ea	1772.7	1
65		1778.5	1
135	e (ea	1778.6	1
331	e (ea	1780.5	1
336		1780.5	0
1034	A2	1790.4	0
213		1793.0	1
828	e (ea	1799.5	1
516	e (ea	1801.1	1
80	e (ea	1807.6	1
733	e (ea	1808.7	1
1503		1809.3	0
1051		1811.3	0
581		1812.6	1
439	e (ea	1819.0	1
847	e (ea	1823.0	1
749	ay	1826.8	1
210	ay	1828.6	1
691	e (ea	1833.1	1
441		1834.1	1
328	e (ea	1836.2	1
683	e (ea	1838.0	1
1024		1839.9	0
106	e (ea	1843.1	1
869	e (ea	1851.4	1
1486		1855.1	0
155	e (ea	1858.1	1

Temple ID	Pedestal	Area	Moat
238	e (ea	1858.8	1
5730	ay	1860.5	0
666	e (ea	1861.7	1
632		1863.1	0
168	e (ea	1871.2	1
834	e (ea	1872.1	1
206	e (ea	1875.7	1
1052		1886.8	0
258	e (ea	1888.6	1
778		1890.2	0
852	e (ea	1892.9	1
294		1897.9	1
753	ay	1908.5	1
355	e (ea	1913.1	1
1044		1916.3	0
73	e (ea	1922.9	1
71		1925.0	0
1038		1925.5	0
82	e (ea	1927.3	1
141	e (ea	1928.5	1
207	e (ea	1933.2	1
1488		1937.2	
90	e (ea	1941.3	1
389	e (ea	1941.7	1
735	e (ea	1958.5	1
185	e (no	1959.6	1
678	e (ea	1963.5	1
367	e (ea	1964.8	1
379	e (ea	1965.1	1
624		1969.3	0
324	e (ea	1970.3	1
388	ay	1976.0	1

Temple ID	Pedestal	Area	Moat
831	oe (e	1978.2	1
1464		1986.0	0
1520		1993.7	0
889		1994.9	0
423	e (ea	1996.2	1
668	e (ea	1998.1	1
521	e (ea	2001.7	1
196	A2	2003.0	1
503	e (ea	2007.9	1
534	e (ea	2009.2	1
370	e (ea	2010.2	1
429	e (ea	2013.6	1
21	e (ea	2014.7	1
63	e (ea	2018.8	1
395		2020.5	1
907		2022.7	0
15	e (ea	2023.5	1
769	e (ea	2031.1	1
588	e (ea	2032.6	1
129	e (ea	2033.4	1
169	e (ea	2039.0	1
653	e (ea	2044.8	1
113	e (ea	2045.8	1
133	e (ea	2050.0	1
243	e (ea	2051.8	1
117	e (ea	2052.0	1
867	ay	2056.7	1
446	e (ea	2059.6	1
606		2061.0	0
494		2067.1	0
539	e (ea	2069.3	1
471	e (ea	2069.8	1

Temple ID	Pedestal	Area	Moat
303	e (ea	2081.0	1
626		2082.5	0
587		2083.5	0
9		2087.7	0
1001		2093.2	0
143	e (ea	2095.7	1
601		2097.1	0
1012		2100.1	0
107	e (ea	2102.8	1
342	e (ea	2103.6	1
200	e (ea	2109.7	1
612	e (ea	2112.6	1
743		2115.3	0
619		2116.4	0
281		2116.9	0
846	e (ea	2118.2	1
175	e (ea	2122.4	1
140	e (ea	2124.1	1
59	ay	2124.3	1
436	e (ea	2125.6	1
567		2134.7	0
855		2136.3	1
259	e (ea	2141.2	1
483	e (ea	2143.9	1
23	A2	2150.5	1
70	ay	2150.8	1
548	e (ea	2158.6	1
1077		2158.7	0
792		2160.3	1
997		2162.7	0
543	e (ea	2166.5	1
497	e (ea	2166.8	1

Temple ID	Pedestal	Area	Moat
809		2177.7	0
27	e (ea	2184.0	1
1028		2195.7	0
687	e (ea	2199.1	1
673	e (ea	2200.0	1
289	e (ea	2200.2	1
614	e (ea	2206.1	1
253		2214.4	0
34	e (ea	2216.3	1
399	A2	2216.3	1
720		2216.9	0
659		2219.7	1
275	e (ea	2220.8	1
681	e (ea	2228.6	1
248		2229.0	0
509	e (ea	2232.3	1
887		2236.6	0
403	e (ea	2237.4	1
25	e (ea	2240.5	1
765		2255.6	0
6601		2261.4	0
6999		2266.6	
40	e (ea	2271.8	1
1	e (ea	2278.3	1
136	e (ea	2278.5	1
596		2279.1	1
705		2281.2	1
487		2301.4	0
24	e (ea	2302.9	1
202	e (ea	2310.6	1
873	e (ea	2317.8	1
418	e (ea	2330.4	1

Temple ID	Pedestal	Area	Moat
6	ıe (ea	2334.3	1
527		2334.7	0
1014		2344.8	0
335	ıe (ea	2358.6	1
159	ay	2359.0	1
114	ay	2362.3	1
89		2365.4	0
634		2365.5	0
536		2366.6	1
325	ıe (ea	2374.2	1
351	ıe (ea	2374.4	1
633	ıe (ea	2377.8	1
416	ıe (ea	2380.4	1
1482		2390.4	0
358	ıe (ea	2405.0	1
823	ıe (ea	2406.7	1
165	ıe (ea	2426.0	1
747	ıe (ea	2432.0	1
868		2437.4	0
438	ıe (ea	2440.1	1
1018		2440.5	0
406	ıe (ea	2446.1	1
1448		2451.1	1
640	A2	2454.5	1
862	B	2456.0	1
854	ıe (ea	2462.2	1
350	ıe (ea	2464.0	1
689	ıe (ea	2465.2	1
1040		2467.2	0
224	ıe (ea	2468.2	1
396	ıe (ea	2485.2	1
560	ıe (ea	2495.9	1

Temple ID	Pedestal	Area	Moat
579		2504.3	0
671	e (ea	2510.0	1
842		2516.7	0
6811		2522.8	0
398	e (ea	2528.9	1
430	e (ea	2536.8	1
154	e (ea	2542.7	1
219	e (ea	2543.3	1
609		2544.7	0
201	e (ea	2547.9	1
557	A2	2551.8	0
189	B	2552.0	1
1359		2555.1	0
649		2555.4	0
316	e (ea	2570.0	1
68		2573.8	0
537		2580.3	0
363	e (ea	2585.1	1
615	e (ea	2585.8	1
547	ay	2588.8	1
160		2590.8	0
298	B	2605.8	0
353	e (ea	2607.1	1
443	A2	2613.7	1
230	e (ea	2616.2	1
102	e (ea	2616.7	1
187	e (ea	2648.4	1
861	e (ea	2658.1	1
590	e (ea	2669.7	1
179		2684.1	1
5		2687.6	0
1088		2699.7	0

Temple ID	Pedestal	Area	Moat
827		2700.2	1
249	ay	2708.7	1
6774	e (ea	2713.2	1
29	e (no	2716.1	1
864		2717.2	0
589		2725.8	0
444	e (ea	2747.3	1
638	A1	2761.0	0
167	e (ea	2765.8	1
650	e (ea	2775.3	1
157		2778.4	1
629		2793.9	0
1050		2805.3	0
594		2817.0	0
518	e (ea	2817.8	1
11	e (ea	2825.7	1
375	e (ea	2857.2	1
123		2860.8	0
575		2868.5	1
817		2876.1	0
347	e (ea	2895.1	1
1022		2908.9	0
1056		2918.4	0
482		2925.1	0
422	e (ea	2928.4	1
1476		2932.4	0
670		2939.9	0
622	ay	2941.5	1
752		2949.0	0
329		2953.7	0
198		2960.4	0
591	e (ea	2962.5	1

Temple ID	Pedestal	Area	Moat
651	e (ea	2962.7	1
372	e (ea	2981.3	1
1063		2981.9	0
1046		2983.9	0
549	e (ea	2984.6	1
247		2989.5	0
197	e (ea	2989.5	1
271	e (ea	3017.0	1
1039		3019.8	0
402	e (ea	3027.5	1
535	e (ea	3029.0	1
153	ay	3030.8	1
1065		3032.0	0
142	e (ea	3037.2	1
84	e (ea	3041.2	1
495		3042.9	0
564	e (ea	3043.4	1
326	e (ea	3043.6	1
628	A4	3045.4	0
1467	e (ea	3046.5	1
1388		3047.4	0
1492		3056.3	0
163	e (ea	3075.4	1
425		3093.6	0
1523		3102.5	0
357	e (ea	3103.6	1
1484		3103.9	0
371		3115.9	0
413	e (ea	3121.2	1
1032		3126.6	0
563	B	3130.9	1
190	ay	3136.7	1

Temple ID	Pedestal	Area	Moat
151	e (ea	3140.5	1
139		3141.3	1
625		3149.3	1
33	ay	3158.2	1
569	e (ea	3171.9	1
314	ay	3181.9	1
6573		3185.2	0
6755		3185.3	0
546	e (ea	3198.4	1
672	e (ea	3204.6	1
112	e (ea	3225.0	1
1519		3231.4	0
819	ay	3234.0	1
260	e (ea	3241.3	1
859		3242.1	0
1360		3244.1	0
2	e (ea	3245.7	1
550		3245.9	1
382		3249.2	0
39	ay	3256.7	1
174	e (ea	3258.6	1
660	e (ea	3272.3	1
502	A2	3274.5	0
393	A2	3274.7	0
38	e (ea	3276.9	1
1475	e (we	3282.0	0
1026		3292.8	1
1461		3294.3	0
724	ay	3296.0	1
162	e (ea	3324.9	1
10	e (ea	3348.9	1
476	e (ea	3349.4	1

Temple ID	Pedestal	Area	Moat
1642		3383.6	0
865	ay	3390.4	1
1479		3391.0	0
130	e (ea	3397.9	1
643	e (ea	3414.0	1
746		3433.4	0
654	e (ea	3436.4	1
1389		3447.2	0
1438		3458.2	0
8	e (ea	3463.8	1
593		3470.4	0
1511		3497.4	0
759	A2	3522.7	1
209		3532.4	1
115	e (ea	3584.8	1
621	ay	3594.4	1
211		3606.1	1
414	e (ea	3613.3	1
835		3613.3	0
305	ay	3655.3	1
152		3680.2	0
79	e (ea	3708.7	1
158	e (ea	3720.0	1
6015	ay	3731.0	1
16		3743.3	1
822		3757.6	0
45		3759.7	1
904	e (ea	3775.1	0
616	e (ea	3789.7	1
307	e (no	3794.7	1
531		3809.5	0
473	e (ea	3828.4	1

Temple ID	Pedestal	Area	Moat
571	e (ea	3829.8	1
43	e (ea	3860.1	1
1073	e (we	3861.3	1
83	ay	3862.2	1
646	ay	3867.2	1
228	e (ea	3885.1	1
605	e (ea	3890.7	1
648	e (ea	3891.2	1
496	e (ea	3906.7	1
617	e (ea	3928.1	1
105	e (ea	3931.0	1
3	e (ea	3931.7	1
352		3941.3	1
474	e (ea	3947.0	1
257	e (ea	3969.9	1
688		3994.9	1
806	A2	4000.6	1
1524		4007.4	0
266	e (ea	4031.4	1
1441		4033.8	0
412	e (ea	4048.5	1
424	e (ea	4062.6	1
485		4069.1	1
498	e (ea	4077.5	1
1490		4092.3	0
86	e (ea	4099.4	1
475	ay	4160.4	1
493		4161.1	0
1041		4186.6	0
1474		4199.2	0
225	e (ea	4200.1	1
1090		4219.4	0

Temple ID	Pedestal	Area	Moat
1166		4229.2	0
284	e (ea	4243.7	1
1357	A1	4259.5	0
1497		4274.6	0
1471		4275.4	0
176	e (ea	4314.4	1
478	e (ea	4316.7	1
1721		4316.7	1
428		4327.1	1
407	e (ea	4332.8	1
264	e (ea	4351.0	1
1496	e (ea	4375.0	1
6965		4399.2	0
137	e (ea	4402.5	1
170	e (ea	4406.5	1
337	e (ea	4424.2	1
791	e (ea	4438.9	1
607	e (ea	4451.7	1
311	A2	4452.4	1
510		4473.3	0
511	e (ea	4542.0	1
320	A1	4562.9	0
754		4615.6	0
359		4618.3	0
538	e (no	4630.8	1
282		4635.9	0
262	e (ea	4663.4	1
807	A2	4669.3	1
913		4681.8	0
914		4681.8	0
584		4710.9	0
246	e (ea	4745.3	1

Temple ID	Pedestal	Area	Moat
1513	ıe (ea	4762.2	1
644	ıe (ea	4778.9	1
188	ıe (ea	4786.9	1
7040		4943.4	0
692		4958.2	1
173	ıe (ea	4961.6	1
387		4965.2	1
1500		5005.5	0
1439		5059.4	0
645	ay	5069.7	1
1019		5086.0	0
278		5108.4	1
1454		5129.6	0
690	ıe (ea	5157.0	1
1518		5162.0	0
1363		5207.3	1
99		5224.4	0
1432		5225.1	0
1364		5235.3	0
1427		5288.9	
1458		5307.5	1
1466		5308.0	0
6013		5309.3	0
145	ıe (ea	5348.2	1
1435		5394.1	0
377		5417.6	1
1433		5425.6	0
156	ıe (ea	5480.1	1
60	A1	5494.0	1
1485		5494.9	0
1470		5606.5	0
110		5620.3	1

Temple ID	Pedestal	Area	Moat
365	A2	5769.1	1
6004	e (ea	5779.2	1
1505		5799.3	0
348	e (ea	5857.7	1
146	e (ea	5920.9	1
1521		5927.1	0
637		5981.3	0
731	ay	6147.4	1
701		6206.8	1
721	e (ea	6212.8	1
300		6245.5	0
81		6247.5	0
67	e (ea	6249.2	1
178	ay	6257.0	1
481	e (ea	6270.7	1
810	ay	6295.8	1
181		6375.5	0
1430		6411.5	0
192	ay	6427.9	1
1468		6486.2	0
66	e (ea	6700.3	1
315		6712.4	1
448		6739.6	0
1514		6802.1	1
522	ay	6826.8	1
1437		6833.2	0
1506		6869.8	0
1477		6927.4	0
488	e (ea	6974.0	1
826		6985.4	1
572		7165.5	0
726		7188.9	0

Temple ID	Pedestal	Area	Moat
1058	ay	7266.6	1
1504		7273.2	0
172	e (ea	7274.8	1
1451		7306.9	0
1440		7324.8	0
814	A2	7346.5	1
1494		7348.7	0
376		7351.3	0
1517		7402.1	0
341		7424.2	0
1459		7433.9	1
525	A2	7517.4	1
1526		7554.2	0
6006	ay	7655.5	1
477	e (ea	7750.3	1
830	A1	7815.2	1
1481		7943.3	0
1047	e (ea	8114.8	1
339	e (ea	8120.6	1
1509		8215.8	0
1473		8270.4	0
1515		8415.4	0
171		8440.8	1
1457		8454.3	0
378	e (ea	8472.1	1
409	e (ea	8581.3	1
1021		8583.0	0
562	e (ea	8598.8	1
1465		8841.3	0
1493		8841.9	0
1719		8883.6	0
717		8904.7	0

Temple ID	Pedestal	Area	Moat
1358		9338.1	0
28		9476.8	0
789		9480.2	0
1469		9530.1	0
489		9564.3	0
1449		9718.7	0
263	e (ea	###	1
345		###	0
630		###	1
1501		###	0
124		###	1
1460		###	0
191	ay	###	1
1442		###	0
1455		###	0
627	e (ea	###	1
366		###	0
1453		###	0
265	e (ea	###	1
1060		###	0
533		###	1
544	e (ea	###	1
472	A2	###	0
6579	e (ea	###	1
6791		###	0
620	e (ea	###	0
479	e (ea	###	1
790		###	0
1507		###	1
658	e (ea	###	1
1087		###	0
1527		###	0

Temple ID	Pedestal	Area	Moat
6780		###	0
1491		###	0
796	e (ea	###	1
5729	ay	###	0
1429		###	0
180		###	0
1356		###	0
1289		###	0
1362		###	1
815	A4	###	0
1443		###	0
818	ay	###	1
317		###	0
1390		###	0
1428		###	0
890	N/a	###	0
1502		###	0
1508		###	0
1472		###	0
131		###	0
128		###	0
6084	ay	###	1
1462		###	0
788	ay	###	1
6656		###	0
386	ay	###	1
1277	e (we	###	1
203		###	1
891		###	0
530	ay	###	1
885	A2	###	0
1452		###	0

Temple ID	Pedestal	Area	Moat
1512		###	0
1447		###	0
6915		###	0
1431		###	0
6920		###	0
1632	ay	###	1
1613	ay	###	1
1483		###	0
1495	e (ea	###	1
6677		###	0
6964		###	0
1463		###	0
6908		###	0
6688		###	1
6914		###	0
6917		###	0
552		###	1
6962		###	0
6913		###	0
6956		###	0
36	A4	###	1
603	ay	###	1
886	ay	###	1
1662	ay	###	1
6911		###	0
6918		###	0
6919		###	0
6912		###	0
6909		###	0
1093			
1094			
1100			

Temple ID	Pedestal	Area	Moat
1102			
1103			
1105			
1106			
1107			
1108			
1109			
1110			
1111			
1112			
1113			
1115			
1116			
1119			
1120			
1121			
1122			
1123			
1124			
1125			
1126			
1127			
1128	A2		
1129			
1130			
1131			
1132			
1133			
1134			
1135			
1136			
1137			

Temple ID	Pedestal	Area	Moat
1172			
1173			
1174			
1175			
1176			
1177			
1178			
1179			
1180			
1181			
1182			
1183			
1184			
1185			
1186	A4		
1187			
1188			
1189			
1190			
1191			
1192			
1193			
1194			
1195			
1196			
1197			
1198			
1199			
1200			
1201			
1202			
1203			

Temple ID	Pedestal	Area	Moat
1579			
1588			
1590			
1633			
1640			
1668			
1669			
1672			
1677	A2		
1681			
1682	A1		
1683	A2		
1684	A1		
1685			
1686	A2		
1688	A2		
1689	B		
1692	A2		
1696	B		
1704	A4		
1705	A4		
1706	A3		
1707			
1708	A1		
1710			
1713			
1715			
1716			
1717			
1718			
1720			
5932			

Temple ID	Pedestal	Area	Moat
5934			
5945			
5949			
5950			
5964			
5968			
5971			
5973			
5974			
5976			
5977			
5978			
5979			
5980			
5985			
5988			
5989			
5991			
5997			
5999			
6007			
6010			
6011			
6012			
6014			
6016			
6018			
6019			
6020			
6029			0
6031			
6047			
