# Supplementary material for: Semi-supervised machine learning approaches for predicting the chronology of archaeological sites: A case study of temples from medieval Angkor, Cambodia
Source: PLoS One. 2018 Nov 5;13(11):e0205649. doi: 10.1371/journal.pone.0205649 (PMC6218026; doi:10.1371/journal.pone.0205649)
Supplement: S3 Table — (PDF) [file pone.0205649.s004.pdf]

Temple_ID	Inferred Temple Date	Specification	Error	GSSL or MLR
1	976.1907577	1	49	MLR
2	1132.732889	0	49	MLR
3	949.5	1	0	Known date
4	970	1	49	GSSL
5	976.1907577	1	49	MLR
6	1132.732889	1	49	MLR
7	930.5792673	1	49	MLR
8	970	1	49	GSSL
9	976.1907577	1	49	MLR
10	1067.197898	1	49	MLR
11	1067.197898	1	49	MLR
12	1010	1	49	GSSL
13	976.1907577	1	49	MLR
14	976.1907577	1	49	MLR
15	865.0442756	1	49	MLR
16	1087.121399	1	49	MLR
17	976.1907577	1	49	MLR
18	1021.586407	1	49	MLR
19	930.5792673	1	49	MLR
20	976.1907577	1	49	MLR
21	839	0	66	GSSL
22	976.1907577	1	49	MLR
23	976.1907577	1	49	MLR
24	976.1907577	0	49	MLR
25	910.655766	1	49	MLR
26	961	1	49	GSSL
27	970	1	49	GSSL
28	926	0	66	GSSL
29	930.5792673	1	49	MLR
30	1132.732889	0	49	MLR
31	976.1907577	1	49	MLR
32	976.1907577	0	49	MLR
33	976.1907577	1	49	MLR
34	976.1907577	1	49	MLR
36	1087.121399	1	49	MLR
38	1132.732889	1	49	MLR
39	976.1907577	1	49	MLR
40	910.655766	1	49	MLR
41	976.1907577	1	49	MLR
42	976.1907577	1	49	MLR
43	1022	1	49	GSSL
44	1035	1	49	GSSL
45	962	1	49	GSSL
46	976.1907577	1	49	MLR

47	910.655766	1	49	MLR
48	1035	1	49	GSSL
49	976.1907577	1	49	MLR
50	1132.732889	0	49	MLR
51	839	0	66	GSSL
52	976.1907577	0	49	MLR
53	856	0	66	GSSL
54	1132.732889	0	49	MLR
55	976.1907577	0	49	MLR
56	976.1907577	1	49	MLR
57	976.1907577	0	49	MLR
58	1021.586407	0	49	MLR
59	976.1907577	1	49	MLR
60	976.1907577	1	49	MLR
61	943	1	0	Known date
62	1003	1	49	GSSL
63	968	1	0	Known Date
64	910.655766	1	49	MLR
65	962	1	49	GSSL
66	1021.586407	1	49	MLR
67	865.0442756	1	49	MLR
68	976.1907577	1	49	MLR
69	865.0442756	0	49	MLR
70	976.1907577	1	49	MLR
71	976.1907577	0	49	MLR
72	976.1907577	0	49	MLR
73	929	1	49	GSSL
74	976.1907577	1	49	MLR
76	1132.732889	1	49	MLR
77	1132.732889	1	49	MLR
78	930.5792673	1	49	MLR
79	970	1	49	GSSL
80	1132.732889	0	49	MLR
81	976.1907577	1	49	MLR
82	976.1907577	1	49	MLR
83	930.5792673	1	49	MLR
84	1067.197898	1	49	MLR
85	1132.732889	1	49	MLR
86	976.1907577	1	49	MLR
87	856	0	66	GSSL
88	875	0	66	GSSL
89	1132.732889	1	49	MLR
90	910.655766	0	49	MLR
91	1087.121399	1	49	MLR
92	1299.5	1	0	Known date

93	1067.197898	0	49	MLR
94	858	0	66	GSSL
95	910.655766	1	49	MLR
96	839	0	66	GSSL
97	1132.732889	0	49	MLR
98	1114	1	49	GSSL
99	976.1907577	1	49	MLR
100	856	0	66	GSSL
101	976.1907577	1	49	MLR
102	1132.732889	1	49	MLR
103	1132.732889	1	49	MLR
104	976.1907577	1	49	MLR
105	1041	1	49	GSSL
106	1132.732889	1	49	MLR
107	1087.121399	0	49	MLR
108	856	0	66	GSSL
109	1111	1	49	GSSL
110	1087.121399	1	49	MLR
111	976.1907577	1	49	MLR
112	1308	1	0	Known date
113	976.1907577	0	49	MLR
114	979	1	49	GSSL
115	976.1907577	1	49	MLR
116	976.1907577	0	49	MLR
117	976.1907577	1	49	MLR
118	1132.732889	1	49	MLR
120	1004	1	49	GSSL
121	839	0	66	GSSL
122	976.1907577	1	49	MLR
123	976.1907577	1	49	MLR
124	1067.197898	1	49	MLR
125	839	0	66	GSSL
126	976.1907577	1	49	MLR
127	976.1907577	1	49	MLR
128	986	1	49	GSSL
129	910.655766	1	49	MLR
130	970	1	49	GSSL
131	1067.197898	1	49	MLR
132	1021.586407	1	49	MLR
133	1132.732889	1	49	MLR
134	1021.586407	0	49	MLR
135	980	1	49	GSSL
136	865.0442756	1	49	MLR
137	865.0442756	1	49	MLR
138	865.0442756	1	49	MLR

139	875	0	66	GSSL
140	976.1907577	0	49	MLR
141	976.1907577	0	49	MLR
142	930.5792673	1	49	MLR
143	1111	1	49	GSSL
144	1067.197898	1	49	MLR
145	985	1	49	GSSL
146	929	1	49	GSSL
147	929	1	49	GSSL
148	976.1907577	1	49	MLR
149	1109	1	49	GSSL
151	930.5792673	1	49	MLR
152	986	1	49	GSSL
153	960	1	49	GSSL
154	910.655766	1	49	MLR
155	856	0	66	GSSL
156	1090	1	0	Known date
157	930.5792673	0	49	MLR
158	910.655766	0	49	MLR
159	1067.197898	1	49	MLR
160	1132.732889	1	49	MLR
161	976.1907577	0	49	MLR
162	978	1	49	GSSL
163	1132.732889	1	49	MLR
164	1132.732889	0	49	MLR
165	976.1907577	1	49	MLR
166	1132.732889	0	49	MLR
167	1035	1	49	GSSL
168	1021.586407	1	49	MLR
169	1067.197898	1	49	MLR
170	976.1907577	1	49	MLR
171	976.1907577	1	49	MLR
172	1021.586407	1	49	MLR
173	899.5	1	0	Known date
174	1132.732889	1	49	MLR
175	976.1907577	0	49	MLR
176	790	0	0	Known date
177	865.0442756	1	49	MLR
178	1071	1	0	Known date
179	1132.732889	0	49	MLR
180	977	1	49	GSSL
181	986	1	66	GSSL
183	930.5792673	1	57	MLR
184	976.1907577	1	57	MLR
185	976.1907577	1	57	MLR

186	970	1	0	Known date
187	976.1907577	1	57	MLR
188	980	1	0	Known date
189	985	1	66	GSSL
190	976.1907577	0	57	MLR
191	983	1	0	Known date
192	1067.197898	1	57	MLR
193	910.655766	1	57	MLR
196	976.1907577	1	57	MLR
197	930.5792673	1	57	MLR
198	1132.732889	0	57	MLR
199	976.1907577	1	57	MLR
200	976.1907577	1	57	MLR
201	930.5792673	1	57	MLR
202	976.1907577	1	57	MLR
203	976.1907577	1	57	MLR
204	1035	1	66	GSSL
205	976.1907577	1	57	MLR
206	1132.732889	0	57	MLR
207	1132.732889	0	57	MLR
208	976.1907577	1	57	MLR
209	976.1907577	1	57	MLR
210	910.655766	1	57	MLR
211	976.1907577	1	57	MLR
212	1132.732889	0	57	MLR
213	986	1	66	GSSL
214	1067.197898	0	57	MLR
215	976.1907577	1	57	MLR
216	976.1907577	0	57	MLR
217	976.1907577	1	57	MLR
218	1067.197898	0	57	MLR
219	1087.121399	1	57	MLR
220	976.1907577	1	57	MLR
221	976.1907577	1	57	MLR
222	930.5792673	1	57	MLR
223	1087.121399	0	57	MLR
224	1132.732889	1	57	MLR
225	963	1	66	GSSL
226	1132.732889	0	57	MLR
227	1132.732889	0	57	MLR
228	976.1907577	1	57	MLR
229	1132.732889	1	57	MLR
230	1087.121399	1	57	MLR
231	976.1907577	0	57	MLR
232	910.655766	1	57	MLR

233	1087.121399	1	57	MLR
234	1087.121399	1	57	MLR
235	1132.732889	1	57	MLR
236	976.1907577	0	57	MLR
237	1132.732889	0	57	MLR
238	1087.121399	1	57	MLR
239	1087.121399	1	57	MLR
240	1087.121399	1	57	MLR
241	1087.121399	0	57	MLR
242	976.1907577	1	57	MLR
243	976.1907577	1	57	MLR
244	1067.197898	0	57	MLR
245	1132.732889	1	57	MLR
246	1132.732889	0	57	MLR
247	992	1	66	GSSL
248	1021.586407	1	57	MLR
249	1021.586407	1	57	MLR
250	1132.732889	1	57	MLR
252	1132.732889	0	57	MLR
253	930.5792673	1	57	MLR
254	976.1907577	1	57	MLR
255	810	1	130	GSSL
256	856	0	66	GSSL
257	880	1	0	Known date
257	1075	1	0	Known date
258	985	1	66	GSSL
259	1067.197898	1	57	MLR
260	956	1	0	Known date
262	960	1	0	Known date
263	969	1	0	Known date
264	1002	1	0	Known date
265	983	1	0	Known date
265	865	1	0	Known date
266	930.5792673	1	57	MLR
267	930.5792673	0	57	MLR
268	1132.732889	1	57	MLR
269	976.1907577	0	57	MLR
270	1087.121399	0	57	MLR
271	856	0	66	GSSL
272	976.1907577	1	57	MLR
274	1132.732889	0	57	MLR
275	976.1907577	1	57	MLR
276	930.5792673	0	57	MLR
277	1132.732889	0	57	MLR
278	930.5792673	1	57	MLR

279	976.1907577	0	57	MLR
280	1087.121399	1	57	MLR
281	976.1907577	1	57	MLR
282	976.1907577	1	57	MLR
283	1111	1	66	GSSL
284	976.1907577	1	57	MLR
285	976.1907577	0	57	MLR
287	976.1907577	1	57	MLR
288	1199	1	0	Known Date
289	976.1907577	1	57	MLR
290	976.1907577	1	57	MLR
291	976.1907577	1	57	MLR
292	1087.121399	0	57	MLR
293	1087.121399	1	57	MLR
294	976.1907577	1	57	MLR
295	1087.121399	1	57	MLR
296	960	1	0	Known date
297	970	1	66	GSSL
298	895	1	0	Known date
299	895	0	0	Known date
300	905	1	0	Known date
302	976.1907577	1	57	MLR
303	1111	1	66	GSSL
304	1075	1	0	Known date
305	976.1907577	1	57	MLR
306	976.1907577	1	57	MLR
307	978	1	0	Known date
307	1050	1	0	Known date
308	976.1907577	1	57	MLR
309	910.655766	0	57	MLR
310	1132.732889	0	57	MLR
311	976.1907577	1	57	MLR
312	930.5792673	1	57	MLR
313	930.5792673	1	57	MLR
314	930.5792673	1	57	MLR
315	976.1907577	0	57	MLR
316	976.1907577	1	57	MLR
317	976.1907577	1	57	MLR
318	1132.732889	1	57	MLR
319	976.1907577	1	57	MLR
320	899.5	1	0	Known date
321	1004	1	66	GSSL
322	976.1907577	1	57	MLR
323	976.1907577	1	57	MLR
324	1132.732889	1	57	MLR

325	1067.197898	1	57	MLR
326	910.655766	1	57	MLR
328	1021.586407	1	57	MLR
329	976.1907577	0	57	MLR
330	985	1	66	GSSL
331	1132.732889	1	57	MLR
332	976.1907577	1	57	MLR
333	865.0442756	1	57	MLR
334	930.5792673	1	57	MLR
335	905	1	0	Known date
336	930.5792673	1	57	MLR
337	976.1907577	1	57	MLR
338	915	1	0	Known date
339	930.5792673	1	57	MLR
340	839	0	66	GSSL
341	992	1	66	GSSL
342	980	1	66	GSSL
343	970	1	66	GSSL
344	865.0442756	1	57	MLR
345	976.1907577	1	57	MLR
346	1059	1	66	GSSL
347	976.1907577	1	57	MLR
348	910.655766	1	57	MLR
349	930.5792673	1	57	MLR
350	910.655766	1	57	MLR
351	865.0442756	1	57	MLR
352	1067.197898	0	57	MLR
353	1021.586407	1	57	MLR
354	1067.197898	1	57	MLR
355	910.655766	1	57	MLR
356	1021.586407	1	57	MLR
357	976.1907577	1	57	MLR
358	1087.121399	1	57	MLR
359	976.1907577	1	57	MLR
360	963	1	66	GSSL
361	1041	1	66	GSSL
362	976.1907577	1	57	MLR
363	930.5792673	1	57	MLR
364	1111	1	66	GSSL
365	930.5792673	1	57	MLR
366	977	1	66	GSSL
367	1132.732889	1	57	MLR
368	976.1907577	1	57	MLR
369	865.0442756	1	57	MLR
370	976.1907577	1	57	MLR

371	976.1907577	1	57	MLR
372	1067.197898	1	57	MLR
374	1111	1	66	GSSL
375	930.5792673	0	57	MLR
376	999	1	66	GSSL
377	1067.197898	1	57	MLR
378	1087.121399	1	57	MLR
379	976.1907577	1	57	MLR
380	1132.732889	1	57	MLR
381	1087.121399	1	57	MLR
382	1087.121399	0	57	MLR
383	930.5792673	0	57	MLR
384	1132.732889	0	57	MLR
386	970	1	0	Known date
387	1021.586407	1	57	MLR
388	930.5792673	1	57	MLR
389	1087.121399	1	57	MLR
390	1087.121399	1	57	MLR
391	1067.197898	1	57	MLR
392	1132.732889	1	57	MLR
393	1149.5	1	0	Known date
395	910.655766	0	57	MLR
396	930.5792673	1	57	MLR
397	1087.121399	1	57	MLR
398	1087.121399	1	57	MLR
399	910.655766	1	57	MLR
400	976.1907577	1	57	MLR
401	1007.5	1	0	Known date
402	923	1	66	GSSL
403	1132.732889	1	57	MLR
404	1021.586407	1	57	MLR
405	910.655766	1	57	MLR
406	985	1	66	GSSL
407	976.1907577	0	57	MLR
408	1132.732889	1	57	MLR
409	970	1	66	GSSL
410	929	1	66	GSSL
412	930.5792673	1	57	MLR
413	865.0442756	1	57	MLR
414	1087.121399	1	57	MLR
415	1087.121399	1	57	MLR
416	1041	1	66	GSSL
417	976.1907577	1	57	MLR
418	865.0442756	1	57	MLR
419	839	0	66	GSSL

420	1132.732889	1	57	MLR
421	875	0	66	GSSL
422	1067.197898	1	57	MLR
423	1087.121399	1	57	MLR
424	1087.121399	1	57	MLR
425	976.1907577	1	57	MLR
426	976.1907577	1	57	MLR
427	985	1	66	GSSL
428	1067.197898	1	57	MLR
429	1132.732889	1	57	MLR
430	930.5792673	1	57	MLR
431	985	1	66	GSSL
432	1021.586407	1	57	MLR
433	1132.732889	1	57	MLR
434	910.655766	1	57	MLR
435	1021.586407	1	57	MLR
436	910.655766	0	57	MLR
437	1087.121399	1	57	MLR
438	1132.732889	1	57	MLR
439	1067.197898	1	57	MLR
440	976.1907577	1	57	MLR
441	976	1	66	GSSL
442	1132.732889	1	57	MLR
443	1132.732889	1	57	MLR
444	865.0442756	0	57	MLR
445	1087.121399	1	57	MLR
446	976.1907577	1	57	MLR
447	963	1	0	Known date
448	1067.197898	1	57	MLR
469	1087.121399	1	57	MLR
470	930.5792673	1	57	MLR
471	976.1907577	1	57	MLR
472	893	0	0	Known date
472	949.5	0	0	Known date
473	882.5	1	0	Known date
474	949.5	1	0	Known date
475	710	0	0	Known date
475	883	0	0	Known date
476	1132.732889	1	57	MLR
477	1132.732889	0	57	MLR
478	1021.586407	0	57	MLR
479	879	0	0	Known date
480	1067.197898	0	57	MLR
481	1132.732889	0	57	MLR
482	1021.586407	1	57	MLR

483	1132.732889	1	57	MLR
484	947	1	0	Known date
485	1132.732889	0	57	MLR
486	930.5792673	1	57	MLR
487	1067.197898	1	57	MLR
488	1067.197898	1	57	MLR
489	1132.732889	1	57	MLR
490	1132.732889	1	57	MLR
491	985	1	66	GSSL
492	1067.197898	1	57	MLR
493	976.1907577	1	57	MLR
494	1021.586407	1	57	MLR
495	982	1	66	GSSL
496	1132.732889	1	57	MLR
497	1041	1	66	GSSL
498	1087.121399	1	57	MLR
499	1087.121399	0	57	MLR
500	976.1907577	0	57	MLR
501	1132.732889	1	57	MLR
502	890	1	0	Known date
503	976.1907577	1	57	MLR
504	856	0	66	GSSL
506	976.1907577	1	57	MLR
507	865.0442756	0	57	MLR
508	1087.121399	1	57	MLR
509	930.5792673	1	57	MLR
510	930.5792673	1	57	MLR
511	1132.732889	1	57	MLR
512	865.0442756	1	57	MLR
513	1021.586407	1	57	MLR
514	910.655766	0	57	MLR
515	996	1	66	GSSL
516	1021.586407	0	57	MLR
517	1067.197898	1	57	MLR
518	1021.586407	1	57	MLR
519	1087.121399	1	57	MLR
520	1067.197898	1	57	MLR
521	1152	1	139	GSSL
522	910.655766	1	57	MLR
523	1200	1	0	Known Date
524	1021.586407	1	57	MLR
525	849.5	0	0	Known date
525	880	0	0	Known date
526	865.0442756	1	57	MLR
527	910.655766	1	57	MLR

528	865.0442756	1	57	MLR
529	865.0442756	1	57	MLR
530	976.1907577	1	57	MLR
531	1132.732889	1	57	MLR
532	1087.121399	1	57	MLR
533	1087.121399	1	57	MLR
534	930.5792673	1	57	MLR
535	1087.121399	1	57	MLR
536	976.1907577	1	57	MLR
537	976.1907577	1	57	MLR
538	1067.197898	1	57	MLR
539	930.5792673	0	57	MLR
540	1111	1	66	GSSL
541	976.1907577	1	57	MLR
542	1132.732889	1	57	MLR
543	1049.5	1	0	Known date
544	976.1907577	0	57	MLR
546	976.1907577	1	57	MLR
547	1043	1	66	GSSL
548	865.0442756	0	57	MLR
549	949.9	1	0	Known date
549	1002	1	0	Known date
550	1132.732889	0	57	MLR
551	1021.586407	1	57	MLR
552	1087.121399	1	57	MLR
553	1132.732889	1	57	MLR
554	865.0442756	1	57	MLR
555	1067.197898	0	57	MLR
556	976.1907577	1	57	MLR
557	1021.586407	1	57	MLR
558	976.1907577	0	57	MLR
559	1087.121399	1	57	MLR
560	1067.197898	0	57	MLR
561	992	1	66	GSSL
562	976.1907577	1	57	MLR
563	930.5792673	0	57	MLR
564	1132.732889	1	57	MLR
565	936	1	66	GSSL
566	856	0	66	GSSL
567	976.1907577	1	57	MLR
569	1132.732889	1	57	MLR
570	976.1907577	1	57	MLR
571	856	0	66	GSSL
572	1021.586407	1	57	MLR
573	1035	1	66	GSSL

574	865.0442756	1	57	MLR
575	1016	1	66	GSSL
576	930.5792673	1	57	MLR
577	1041	1	66	GSSL
578	1132.732889	1	57	MLR
579	1087.121399	1	57	MLR
580	910.655766	1	57	MLR
581	1021.586407	0	57	MLR
582	1021.586407	1	57	MLR
583	1087.121399	1	57	MLR
584	976.1907577	1	57	MLR
585	1087.121399	1	57	MLR
586	985	1	66	GSSL
587	1067.197898	1	57	MLR
588	1087.121399	1	57	MLR
589	930.5792673	1	57	MLR
590	865.0442756	1	57	MLR
591	1035	1	66	GSSL
592	1135	1	66	GSSL
593	999	1	66	GSSL
594	930.5792673	1	57	MLR
595	910.655766	1	57	MLR
596	976.1907577	1	57	MLR
597	1087.121399	1	57	MLR
598	1087.121399	1	57	MLR
599	1087.121399	1	57	MLR
600	1021.586407	1	57	MLR
601	992	1	66	GSSL
602	976.1907577	1	57	MLR
603	1016	1	0	Known date
604	1021.586407	1	57	MLR
605	1067.197898	0	57	MLR
606	995	1	66	GSSL
607	970	1	66	GSSL
608	999	1	66	GSSL
609	901	0	66	GSSL
610	930.5792673	1	57	MLR
611	976.1907577	0	57	MLR
612	1152	1	139	GSSL
613	1041	1	66	GSSL
614	985	1	66	GSSL
615	1111	1	66	GSSL
616	976.1907577	1	57	MLR
617	856	0	66	GSSL
618	985	1	66	GSSL

619	1132.732889	1	57	MLR
620	1067.197898	1	57	MLR
621	994	1	66	GSSL
622	1005	1	66	GSSL
623	976.1907577	1	57	MLR
624	865.0442756	1	57	MLR
625	927	1	66	GSSL
626	930.5792673	1	57	MLR
627	930.5792673	0	57	MLR
628	983	1	66	GSSL
629	985	1	66	GSSL
630	927	1	66	GSSL
631	930.5792673	1	57	MLR
632	985	1	66	GSSL
633	839	0	66	GSSL
634	865.0442756	1	57	MLR
635	982	1	66	GSSL
636	1132.732889	1	57	MLR
637	1067.197898	1	57	MLR
638	1087.121399	1	57	MLR
639	1021.586407	1	57	MLR
640	1087.121399	1	57	MLR
641	1021.586407	1	57	MLR
642	865.0442756	1	57	MLR
643	1040	1	0	Known date
644	1041	1	66	GSSL
645	976.1907577	1	57	MLR
646	1079	1	66	GSSL
647	985	1	66	GSSL
648	930.5792673	1	57	MLR
649	865.0442756	1	57	MLR
650	1087.121399	1	57	MLR
651	865.0442756	0	57	MLR
652	1021.586407	1	57	MLR
653	865.0442756	1	57	MLR
654	930.5792673	1	57	MLR
655	910.655766	1	57	MLR
656	976.1907577	0	57	MLR
657	986	1	66	GSSL
658	1035	1	66	GSSL
659	930.5792673	1	57	MLR
660	976.1907577	1	57	MLR
661	1021.586407	1	57	MLR
662	1132.732889	1	57	MLR
663	976.1907577	1	57	MLR

664	976.1907577	1	57	MLR
665	1132.732889	1	57	MLR
666	930.5792673	1	57	MLR
668	976.1907577	1	57	MLR
669	1189	1	0	Known date
670	865.0442756	1	57	MLR
671	976.1907577	0	57	MLR
672	976.1907577	1	57	MLR
673	925	1	66	GSSL
675	1087.121399	1	57	MLR
676	930.5792673	0	57	MLR
677	880	0	66	GSSL
678	985	1	66	GSSL
681	930.5792673	1	57	MLR
683	1132.732889	1	57	MLR
684	856	0	66	GSSL
685	865.0442756	1	57	MLR
686	930	1	0	Known date
687	930.5792673	0	57	MLR
688	910.655766	1	57	MLR
689	1021.586407	0	57	MLR
690	1067.197898	1	57	MLR
691	976.1907577	0	57	MLR
692	1067.197898	1	57	MLR
695	930.5792673	1	57	MLR
696	1021.586407	1	57	MLR
697	999	1	66	GSSL
701	1021.586407	1	57	MLR
702	930.5792673	1	57	MLR
703	930.5792673	1	57	MLR
704	1087.121399	1	57	MLR
705	1021.586407	0	57	MLR
706	930.5792673	1	57	MLR
707	865.0442756	1	57	MLR
708	865.0442756	1	57	MLR
709	865.0442756	1	57	MLR
710	865.0442756	0	57	MLR
711	1087.121399	0	57	MLR
713	1087.121399	0	57	MLR
716	1021.586407	1	57	MLR
717	899	1	66	GSSL
718	970	1	66	GSSL
719	1087.121399	1	57	MLR
720	1087.121399	1	57	MLR
721	1200.5	1	0	Known date

721	1249.5	1	0	Known date
722	1041	1	66	GSSL
723	1087.121399	1	57	MLR
724	1087.121399	0	57	MLR
725	1021.586407	1	57	MLR
726	1021.586407	1	57	MLR
728	995	1	66	GSSL
729	1087.121399	0	57	MLR
730	1087.121399	0	57	MLR
731	1021.586407	0	57	MLR
733	1021.586407	0	57	MLR
734	1021.586407	1	57	MLR
735	1021.586407	1	57	MLR
736	1021.586407	0	57	MLR
743	1021.586407	0	57	MLR
746	1021.586407	0	57	MLR
747	839	0	66	GSSL
749	1021.586407	1	57	MLR
750	1021.586407	0	57	MLR
752	901	0	66	GSSL
753	1162	1	139	GSSL
754	1021.586407	1	57	MLR
756	1021.586407	0	57	MLR
759	967	0	0	Known date
759	1301	0	0	Known date
761	893	1	0	Known date
762	875	0	66	GSSL
763	1022	1	66	GSSL
765	986	1	66	GSSL
766	839	0	66	GSSL
768	973	1	66	GSSL
769	970	1	66	GSSL
770	856	0	66	GSSL
771	875	0	66	GSSL
773	873	0	66	GSSL
774	1004	1	66	GSSL
776	856	0	66	GSSL
778	980	0	0	Known date
778	1030	0	0	Known date
781	1021.586407	0	57	MLR
782	810	1	130	GSSL
788	1200.5	1	0	Known date
788	1249.5	1	0	Known date
788	1327	1	0	Known date
789	690	1	0	Known date

790	926	0	66	GSSL
791	865.0442756	1	57	MLR
792	1021.586407	1	57	MLR
793	980	1	66	GSSL
794	1021.586407	1	57	MLR
795	1100	1	0	Known Date
796	893	1	0	Known date
796	1004.5	1	0	Known date
796	1043.5	1	0	Known date
797	1079	1	66	GSSL
799	999	1	66	GSSL
800	1100	0	0	Known Date
806	955	1	0	Known date
807	954	1	0	Known date
809	1021.586407	1	57	MLR
810	1021.586407	0	57	MLR
811	1067.197898	1	57	MLR
812	1021.586407	0	57	MLR
813	1087.121399	0	57	MLR
814	921	1	0	Known date
815	954	1	0	Known date
816	865.0442756	1	57	MLR
817	1067.197898	1	57	MLR
818	961	0	0	Known date
818	1080	0	0	Known date
819	910.655766	1	57	MLR
820	865.0442756	1	57	MLR
821	1150	1	0	Known Date
822	865.0442756	1	57	MLR
823	953	1	0	Known date
824	1067.197898	1	57	MLR
825	930.5792673	1	57	MLR
826	865.0442756	1	57	MLR
827	713	1	0	Known date
828	1021.586407	1	57	MLR
829	1021.586407	0	57	MLR
830	865.0442756	0	57	MLR
831	650	1	0	Known date
831	924.5	1	0	Known date
832	690	1	0	Known date
832	1001	1	0	Known date
833	865.0442756	1	57	MLR
834	910.655766	1	57	MLR
835	1021.586407	1	57	MLR
836	1021.586407	1	57	MLR

837	1021.586407	1	57	MLR
838	1021.586407	1	57	MLR
839	1021.586407	1	57	MLR
840	865.0442756	1	57	MLR
841	1021.586407	1	57	MLR
842	1067.197898	1	57	MLR
843	865.0442756	1	57	MLR
844	865.0442756	1	57	MLR
845	1021.586407	0	57	MLR
846	1021.586407	1	57	MLR
847	910.655766	1	57	MLR
848	865.0442756	0	57	MLR
849	910.655766	0	57	MLR
850	910.655766	1	57	MLR
851	865.0442756	1	57	MLR
852	865.0442756	0	57	MLR
853	865.0442756	1	57	MLR
854	865.0442756	1	57	MLR
855	865.0442756	1	57	MLR
859	910.655766	1	57	MLR
860	865.0442756	1	57	MLR
861	865.0442756	1	57	MLR
862	968	0	0	Known date
864	865.0442756	1	57	MLR
865	865.0442756	1	57	MLR
866	930.5792673	1	57	MLR
867	865.0442756	0	57	MLR
868	865.0442756	1	57	MLR
869	865.0442756	1	57	MLR
870	1021.586407	1	57	MLR
871	865.0442756	1	57	MLR
872	865.0442756	1	57	MLR
873	910.655766	1	57	MLR
874	865.0442756	1	57	MLR
875	900	1	0	Known date
876	995	1	0	Known date
877	865.0442756	1	57	MLR
878	865.0442756	1	57	MLR
883	865.0442756	1	57	MLR
884	865.0442756	1	57	MLR
885	649.5	1	0	Known date
885	895	1	0	Known date
885	968	1	0	Known date
885	999.5	1	0	Known date
885	1049.5	1	0	Known date

886	865.0442756	1	57	MLR
887	865.0442756	1	57	MLR
888	926	0	66	GSSL
889	865.0442756	0	57	MLR
890	1011	1	66	GSSL
891	865.0442756	1	57	MLR
902	1021.586407	0	57	MLR
904	976.1907577	1	57	MLR
905	977	1	66	GSSL
906	982	1	66	GSSL
907	982	1	66	GSSL
908	926	0	66	GSSL
909	977	1	66	GSSL
910	980	1	66	GSSL
912	982	1	66	GSSL
913	977	1	66	GSSL
914	999	1	66	GSSL
927	1005	1	0	Known date
930	999	1	66	GSSL
932	999	1	66	GSSL
933	982	1	66	GSSL
935	999	1	66	GSSL
936	926	0	66	GSSL
937	999	1	66	GSSL
938	982	1	66	GSSL
939	926	0	66	GSSL
940	926	0	66	GSSL
941	970	1	0	Known date
942	999	1	66	GSSL
944	999	1	66	GSSL
948	926	0	66	GSSL
949	926	0	66	GSSL
952	926	0	66	GSSL
955	926	0	66	GSSL
956	926	0	66	GSSL
957	926	0	66	GSSL
958	926	0	66	GSSL
960	926	0	66	GSSL
965	926	0	66	GSSL
968	926	0	66	GSSL
969	926	0	66	GSSL
970	977	1	66	GSSL
973	926	0	66	GSSL
976	901	0	66	GSSL
977	929	1	66	GSSL

978	982	1	66	GSSL
983	901	0	66	GSSL
985	999	1	66	GSSL
987	926	0	66	GSSL
989	926	0	66	GSSL
990	999	1	66	GSSL
996	999	1	66	GSSL
997	995	1	66	GSSL
998	999	1	66	GSSL
999	963	1	66	GSSL
1000	933	1	66	GSSL
1001	985	1	66	GSSL
1002	902	0	0	Known date
1003	899.5	1	0	Known date
1004	960	1	0	Known date
1005	899	1	66	GSSL
1006	986	1	66	GSSL
1007	986	1	66	GSSL
1008	995	1	66	GSSL
1009	996	1	66	GSSL
1010	992	1	66	GSSL
1011	999	1	66	GSSL
1012	999	1	66	GSSL
1013	929	1	66	GSSL
1014	986	1	66	GSSL
1015	958	1	66	GSSL
1016	977	1	66	GSSL
1017	975	1	66	GSSL
1018	976	1	66	GSSL
1019	999	1	66	GSSL
1020	987	1	66	GSSL
1021	986	1	66	GSSL
1022	982	1	66	GSSL
1023	992	1	66	GSSL
1024	929	1	66	GSSL
1025	929	1	66	GSSL
1026	987	1	66	GSSL
1027	982	1	66	GSSL
1028	983	1	66	GSSL
1029	929	1	66	GSSL
1030	983	1	66	GSSL
1031	970	1	0	Known date
1032	983	1	66	GSSL
1033	992	1	66	GSSL
1034	904	1	66	GSSL

1035	933	1	66	GSSL
1036	810	1	130	GSSL
1037	810	1	130	GSSL
1038	983	1	66	GSSL
1039	933	1	66	GSSL
1040	933	1	66	GSSL
1041	986	1	66	GSSL
1042	810	1	130	GSSL
1043	1004	1	66	GSSL
1044	986	1	66	GSSL
1045	933	1	66	GSSL
1046	933	1	66	GSSL
1047	710	1	0	Known date
1047	851	1	0	Known date
1048	977	1	66	GSSL
1049	929	1	66	GSSL
1050	933	1	66	GSSL
1051	949	1	66	GSSL
1052	986	1	66	GSSL
1053	986	1	66	GSSL
1054	929	1	66	GSSL
1055	929	1	66	GSSL
1056	986	1	66	GSSL
1057	986	1	66	GSSL
1058	887	0	0	Known date
1058	949.5	0	0	Known date
1060	933	1	66	GSSL
1061	830	1	0	Known date
1063	815	0	0	Known date
1063	1149.5	0	0	Known date
1065	820	0	0	Known date
1067	926	0	66	GSSL
1069	986	1	66	GSSL
1070	926	0	66	GSSL
1071	929	1	66	GSSL
1072	982	1	66	GSSL
1073	958	1	66	GSSL
1075	875	0	66	GSSL
1076	926	0	66	GSSL
1077	926	0	66	GSSL
1078	875	0	66	GSSL
1079	901	0	66	GSSL
1082	926	0	66	GSSL
1083	840	1	0	Known date
1087	903	0	66	GSSL

1088	903	0	66	GSSL
1090	903	0	66	GSSL
1091	922	0	66	GSSL
1093	885	0	66	GSSL
1094	964	0	66	GSSL
1095	926	0	66	GSSL
1100	885	0	66	GSSL
1102	894	0	66	GSSL
1103	885	0	66	GSSL
1105	991	0	66	GSSL
1106	988	0	66	GSSL
1107	848	0	66	GSSL
1108	1002	0	0	Known date
1109	988	0	66	GSSL
1110	988	0	66	GSSL
1111	848	0	66	GSSL
1112	930	0	66	GSSL
1113	988	0	66	GSSL
1115	988	0	66	GSSL
1116	964	0	66	GSSL
1119	1021	0	66	GSSL
1120	1021	0	66	GSSL
1121	825	0	0	Known date
1122	810	0	0	Known date
1123	1021	0	66	GSSL
1124	1021	0	66	GSSL
1125	880	0	0	Known date
1125	949.5	0	0	Known date
1126	1021	0	66	GSSL
1127	934	0	66	GSSL
1128	1009	1	66	GSSL
1129	1021	0	66	GSSL
1130	1164	0	139	GSSL
1131	1158	0	139	GSSL
1132	1166	0	139	GSSL
1133	1021	0	66	GSSL
1134	1021	0	66	GSSL
1135	1021	0	66	GSSL
1136	1021	0	66	GSSL
1137	938	0	66	GSSL
1138	1166	0	139	GSSL
1139	848	0	66	GSSL
1140	1021	0	66	GSSL
1141	1021	0	66	GSSL
1142	1249.5	0	0	Known Date

1143	1249.5	0	0	Known date
1144	1249.5	0	0	Known date
1145	934	0	66	GSSL
1146	835	0	0	Known date
1147	825	0	0	Known date
1148	825	0	0	Known date
1149	1166	0	139	GSSL
1150	934	0	66	GSSL
1151	934	0	66	GSSL
1152	989	0	0	Known date
1153	1200.5	0	0	Known date
1154	934	0	66	GSSL
1155	899.5	0	0	Known date
1156	1021.586407	0	57	MLR
1158	1021	0	66	GSSL
1159	1021	0	66	GSSL
1160	1021	0	66	GSSL
1161	934	0	66	GSSL
1162	1021	0	66	GSSL
1163	1021	0	66	GSSL
1164	934	0	66	GSSL
1165	1067.197898	0	57	MLR
1166	983	1	66	GSSL
1167	1021.586407	0	57	MLR
1168	1166	0	139	GSSL
1169	1166	0	139	GSSL
1170	1163	0	139	GSSL
1171	1163	0	139	GSSL
1172	930.5792673	0	57	MLR
1173	1166	0	139	GSSL
1174	1163	0	139	GSSL
1175	1009	0	66	GSSL
1176	1021.586407	0	57	MLR
1177	1166	0	139	GSSL
1178	1163	0	139	GSSL
1179	1087.121399	0	57	MLR
1180	1021	0	66	GSSL
1181	865.0442756	0	57	MLR
1182	1166	0	139	GSSL
1183	1021.586407	0	57	MLR
1184	1166	0	139	GSSL
1185	1166	0	139	GSSL
1186	1166	1	139	GSSL
1187	1166	0	139	GSSL
1188	1166	0	139	GSSL

1189	1087.121399	0	57	MLR
1190	1166	0	139	GSSL
1191	1021	0	66	GSSL
1192	1087.121399	0	57	MLR
1193	1166	0	139	GSSL
1194	1021	0	66	GSSL
1195	1166	0	139	GSSL
1196	976.1907577	0	57	MLR
1197	1135	0	66	GSSL
1198	1132.732889	0	57	MLR
1199	1087.121399	0	57	MLR
1200	1166	0	139	GSSL
1201	1166	0	139	GSSL
1202	976.1907577	0	57	MLR
1203	865.0442756	0	57	MLR
1204	1163	0	139	GSSL
1205	865.0442756	0	57	MLR
1206	1163	0	139	GSSL
1207	1163	0	139	GSSL
1208	1163	0	139	GSSL
1209	1135	0	66	GSSL
1210	1132.732889	0	57	MLR
1211	930.5792673	0	57	MLR
1212	1009	0	66	GSSL
1213	1021.586407	0	57	MLR
1214	1166	0	139	GSSL
1215	1021.586407	0	57	MLR
1216	1166	0	139	GSSL
1217	1021.586407	0	57	MLR
1218	1087.121399	0	57	MLR
1219	840	0	0	Known date
1220	1087.121399	0	57	MLR
1221	848	0	66	GSSL
1226	1021.586407	0	57	MLR
1254	848	0	66	GSSL
1256	885	0	66	GSSL
1258	885	0	66	GSSL
1260	830	0	0	Known date
1262	825	0	0	Known date
1277	865	0	66	GSSL
1284	1087.121399	0	57	MLR
1289	921	0	66	GSSL
1303	885	0	66	GSSL
1305	1087.121399	0	57	MLR
1306	976.1907577	0	57	MLR

1307	885	0	66	GSSL
1312	1021.586407	0	57	MLR
1313	930	0	66	GSSL
1314	964	0	66	GSSL
1315	1087.121399	0	57	MLR
1316	988	0	66	GSSL
1318	930	0	66	GSSL
1319	930	0	66	GSSL
1320	930	0	66	GSSL
1321	991	0	66	GSSL
1322	988	0	66	GSSL
1323	991	0	66	GSSL
1324	848	0	66	GSSL
1327	930.5792673	0	57	MLR
1328	964	0	66	GSSL
1329	884	0	66	GSSL
1330	848	0	66	GSSL
1331	884	0	66	GSSL
1332	930	0	66	GSSL
1334	1087.121399	0	57	MLR
1336	964	0	66	GSSL
1337	930	0	66	GSSL
1339	930	0	66	GSSL
1340	930.5792673	0	57	MLR
1349	991	0	66	GSSL
1350	848	0	66	GSSL
1351	885	0	66	GSSL
1356	926	0	66	GSSL
1357	1021.586407	0	57	MLR
1358	908	0	66	GSSL
1359	901	0	66	GSSL
1360	1087.121399	1	57	MLR
1362	869	0	66	GSSL
1363	880	0	66	GSSL
1364	908	0	66	GSSL
1388	901	0	66	GSSL
1389	901	0	66	GSSL
1390	901	0	66	GSSL
1427	918	0	66	GSSL
1428	995	1	66	GSSL
1429	901	0	66	GSSL
1430	977	1	66	GSSL
1431	921	0	66	GSSL
1432	908	0	66	GSSL
1433	908	0	66	GSSL

1434	1132.732889	0	57	MLR
1435	825	1	49	GSSL
1436	1021.586407	0	57	MLR
1437	926	0	66	GSSL
1438	901	0	66	GSSL
1439	958	1	66	GSSL
1440	926	0	66	GSSL
1441	921	0	66	GSSL
1442	977	1	66	GSSL
1443	908	0	66	GSSL
1445	810	1	130	GSSL
1447	921	0	66	GSSL
1448	927	1	66	GSSL
1449	926	0	66	GSSL
1450	880	0	66	GSSL
1451	901	0	66	GSSL
1452	926	0	66	GSSL
1453	926	0	66	GSSL
1454	980	1	66	GSSL
1455	901	0	66	GSSL
1456	921	0	66	GSSL
1457	975	1	66	GSSL
1458	875	0	66	GSSL
1459	869	0	66	GSSL
1460	901	0	66	GSSL
1461	959	1	66	GSSL
1462	921	0	66	GSSL
1463	987	1	66	GSSL
1464	980	1	66	GSSL
1465	921	0	66	GSSL
1466	921	0	66	GSSL
1467	961	1	66	GSSL
1468	810	1	130	GSSL
1469	921	0	66	GSSL
1470	810	1	130	GSSL
1471	901	0	66	GSSL
1472	921	0	66	GSSL
1473	921	0	66	GSSL
1474	901	0	66	GSSL
1475	995	1	66	GSSL
1476	930	1	66	GSSL
1477	901	0	66	GSSL
1478	977	1	66	GSSL
1479	926	0	66	GSSL
1480	977	1	66	GSSL

1481	921	0	66	GSSL
1482	982	1	66	GSSL
1483	980	1	66	GSSL
1484	980	1	66	GSSL
1485	921	0	66	GSSL
1486	999	1	66	GSSL
1487	839	0	66	GSSL
1488	916	0	66	GSSL
1489	875	0	66	GSSL
1490	921	0	66	GSSL
1491	1087.121399	1	57	MLR
1492	1087.121399	0	57	MLR
1493	926	0	66	GSSL
1494	958	0	66	GSSL
1495	970	1	66	GSSL
1496	839	0	66	GSSL
1497	982	1	66	GSSL
1498	926	0	66	GSSL
1499	1087.121399	0	57	MLR
1500	977	1	66	GSSL
1501	921	0	66	GSSL
1502	921	0	66	GSSL
1503	926	0	66	GSSL
1504	908	0	66	GSSL
1505	926	0	66	GSSL
1506	901	0	66	GSSL
1507	869	0	66	GSSL
1508	908	0	66	GSSL
1509	981	1	66	GSSL
1510	901	0	66	GSSL
1511	908	0	66	GSSL
1512	908	0	66	GSSL
1513	1132.732889	0	57	MLR
1514	870	0	66	GSSL
1515	975	1	66	GSSL
1516	908	0	66	GSSL
1517	926	0	66	GSSL
1518	921	0	66	GSSL
1519	825	1	49	GSSL
1520	921	0	66	GSSL
1521	921	0	66	GSSL
1522	921	0	66	GSSL
1523	921	0	66	GSSL
1524	958	1	66	GSSL
1525	926	0	66	GSSL

1526	921	0	66	GSSL
1527	975	1	66	GSSL
1534	1163	0	139	GSSL
1555	884	0	66	GSSL
1560	976.1907577	0	57	MLR
1562	1163	0	139	GSSL
1568	991	0	66	GSSL
1569	1166	0	139	GSSL
1571	1164	0	139	GSSL
1572	1163	0	139	GSSL
1574	1166	0	139	GSSL
1575	1163	0	139	GSSL
1579	1163	0	139	GSSL
1588	1163	0	139	GSSL
1590	934	0	66	GSSL
1613	1193	1	0	Known date
1618	1250	1	0	Known date
1632	1117	1	66	GSSL
1633	1163	0	139	GSSL
1640	1011	0	0	Known date
1642	930.5792673	1	57	MLR
1659	1001	1	66	GSSL
1661	930	1	66	GSSL
1662	1149.5	1	0	Known date
1662	1249.5	1	0	Known date
1665	1151	0	0	Known Date
1668	1025.5	0	0	Known date
1669	991	0	66	GSSL
1672	848	0	66	GSSL
1677	1163	1	139	GSSL
1681	1021.586407	0	57	MLR
1682	904	0	66	GSSL
1683	1021.586407	1	57	MLR
1684	1163	1	139	GSSL
1685	1021.586407	0	57	MLR
1686	904	0	66	GSSL
1688	904	0	66	GSSL
1689	904	0	66	GSSL
1692	904	0	66	GSSL
1696	991	1	66	GSSL
1704	904	0	66	GSSL
1705	904	0	66	GSSL
1706	904	0	66	GSSL
1707	1031	0	66	GSSL
1708	904	0	66	GSSL

1710	934	0	66	GSSL
1713	1166	0	139	GSSL
1715	991	0	66	GSSL
1716	1166	0	139	GSSL
1717	1163	0	139	GSSL
1718	1067.197898	0	57	MLR
1719	903	0	66	GSSL
1720	991	0	66	GSSL
1721	880	1	0	Known date
5729	924	0	66	GSSL
5730	924	0	66	GSSL
5932	991	0	66	GSSL
5934	1166	0	139	GSSL
5945	991	0	66	GSSL
5949	1166	0	139	GSSL
5950	1087.121399	0	57	MLR
5964	1021	0	66	GSSL
5968	1087.121399	0	57	MLR
5971	1087.121399	0	57	MLR
5973	991	0	66	GSSL
5974	991	0	66	GSSL
5976	1166	0	139	GSSL
5977	1166	0	139	GSSL
5978	1087.121399	0	57	MLR
5979	1087.121399	0	57	MLR
5980	1166	0	139	GSSL
5985	1163	0	139	GSSL
5988	1163	0	139	GSSL
5989	991	0	66	GSSL
5991	991	0	66	GSSL
5997	1163	0	139	GSSL
5999	848	0	66	GSSL
6004	839	0	66	GSSL
6005	1053	1	66	GSSL
6006	1079	1	66	GSSL
6007	991	0	66	GSSL
6010	991	0	66	GSSL
6011	1087.121399	0	57	MLR
6012	1021.586407	0	57	MLR
6013	926	0	66	GSSL
6014	1166	0	139	GSSL
6015	1079	1	66	GSSL
6016	991	0	66	GSSL
6018	991	0	66	GSSL
6019	991	0	66	GSSL

6020	991	0	66	GSSL
6029	926	0	66	GSSL
6031	1163	0	139	GSSL
6047	1021	0	66	GSSL
6048	1163	0	139	GSSL
6050	991	0	66	GSSL
6074	991	0	66	GSSL
6077	991	0	66	GSSL
6080	1163	0	139	GSSL
6082	991	0	66	GSSL
6084	1043	1	66	GSSL
6090	1021	0	66	GSSL
6092	1166	0	139	GSSL
6093	991	0	66	GSSL
6094	991	0	66	GSSL
6114	991	0	66	GSSL
6413	1163	0	139	GSSL
6416	991	0	66	GSSL
6425	1163	0	139	GSSL
6432	839	0	66	GSSL
6444	991	0	66	GSSL
6466	991	0	66	GSSL
6469	1166	0	139	GSSL
6473	934	0	66	GSSL
6554	901	0	66	GSSL
6571	1163	0	139	GSSL
6573	901	0	66	GSSL
6579	970	1	66	GSSL
6580	991	0	66	GSSL
6585	1166	0	139	GSSL
6588	1163	0	139	GSSL
6590	934	0	66	GSSL
6591	848	0	66	GSSL
6601	901	0	66	GSSL
6607	991	0	66	GSSL
6620	1163	0	139	GSSL
6621	1163	0	139	GSSL
6633	991	0	66	GSSL
6642	991	0	66	GSSL
6644	991	0	66	GSSL
6656	943	1	66	GSSL
6664	1163	0	139	GSSL
6667	1166	0	139	GSSL
6668	1166	0	139	GSSL
6669	1163	0	139	GSSL

6675	1163	0	139	GSSL
6677	982	1	66	GSSL
6678	839	0	66	GSSL
6685	982	1	66	GSSL
6687	848	0	66	GSSL
6688	927	1	66	GSSL
6694	991	0	66	GSSL
6696	991	0	66	GSSL
6737	934	0	66	GSSL
6741	991	0	66	GSSL
6742	885	0	66	GSSL
6749	848	0	66	GSSL
6751	1021	0	66	GSSL
6755	901	0	66	GSSL
6760	991	0	66	GSSL
6765	991	0	66	GSSL
6768	991	0	66	GSSL
6774	856	0	66	GSSL
6779	1163	0	139	GSSL
6780	901	0	66	GSSL
6786	1166	0	139	GSSL
6791	961	1	66	GSSL
6811	926	0	66	GSSL
6846	1163	0	139	GSSL
6847	902	0	66	GSSL
6901	991	0	66	GSSL
6902	991	0	66	GSSL
6903	991	0	66	GSSL
6904	991	0	66	GSSL
6908	921	0	66	GSSL
6909	977	1	66	GSSL
6911	921	0	66	GSSL
6912	960	1	66	GSSL
6913	960	1	66	GSSL
6914	921	0	66	GSSL
6915	921	0	66	GSSL
6916	1158	0	139	GSSL
6917	921	0	66	GSSL
6918	960	1	66	GSSL
6919	960	1	66	GSSL
6920	921	0	66	GSSL
6948	991	0	66	GSSL
6949	991	0	66	GSSL
6950	991	0	66	GSSL
6951	991	0	66	GSSL

6956	921	0	66	GSSL
6958	991	0	66	GSSL
6959	1166	0	139	GSSL
6960	1166	0	139	GSSL
6961	991	0	66	GSSL
6962	921	0	66	GSSL
6963	991	0	66	GSSL
6964	960	1	66	GSSL
6965	961	1	66	GSSL
6970	1163	0	139	GSSL
6971	885	0	66	GSSL
6972	991	0	66	GSSL
6973	1163	0	139	GSSL
6975	1166	0	139	GSSL
6980	991	0	66	GSSL
6981	991	0	66	GSSL
6982	991	0	66	GSSL
6986	991	0	66	GSSL
6990	1166	0	139	GSSL
6991	848	0	66	GSSL
6994	1163	0	139	GSSL
6996	1166	0	139	GSSL
6999	905	0	66	GSSL
7001	848	0	66	GSSL
7002	934	0	66	GSSL
7004	991	0	66	GSSL
7005	1166	0	139	GSSL
7011	997	0	66	GSSL
7021	921	0	66	GSSL
7034	991	0	66	GSSL
7038	991	0	66	GSSL
7040	926	0	66	GSSL
7045	1166	0	139	GSSL
7047	1163	0	139	GSSL
7049	839	0	66	GSSL
7050	934	0	66	GSSL
7054	1163	0	139	GSSL
7057	1163	0	139	GSSL
7058	1026	0	66	GSSL
7059	848	0	66	GSSL
7060	848	0	66	GSSL
7061	934	0	66	GSSL
7062	934	0	66	GSSL
7074	1163	0	139	GSSL
7075	1163	0	139	GSSL

7076	1163	0	139	GSSL
7081	1163	0	139	GSSL
7082	991	0	66	GSSL
7083	991	0	66	GSSL
7084	1163	0	139	GSSL
7085	991	0	66	GSSL
7091	1166	0	139	GSSL
7092	988	0	66	GSSL
7094	1163	0	139	GSSL
7096	991	0	66	GSSL
7098	1163	0	139	GSSL
7099	991	0	66	GSSL
7100	991	0	66	GSSL
7101	1163	0	139	GSSL
7102	1163	0	139	GSSL
7103	1163	0	139	GSSL
7104	1163	0	139	GSSL
7113	1166	0	139	GSSL
7114	1163	0	139	GSSL
7115	1163	0	139	GSSL
7116	1163	0	139	GSSL
7117	1163	0	139	GSSL
7118	1163	0	139	GSSL
7121	991	0	66	GSSL
